# Multiomics approach identifies SERPINB1 as candidate biomarker for spinocerebellar ataxia type 2

**DOI:** 10.1038/s41598-025-29070-7

**Published:** 2025-11-26

**Authors:** Luis E. Almaguer-Mederos, Jana Key, Nesli-Ece Sen, Julia Canet-Pons, Claudia Döring, David Meierhofer, Suzana Gispert-Sánchez, Dany Cuello-Almarales, Dennis Almaguer-Gotay, Lidia M. Osorio-González, Raúl Aguilera-Rodríguez, Jacqueline Medrano-Montero, Georg Auburger

**Affiliations:** 1 University Hospital, Clinic of Neurology, Exp. Neurology, Goethe University, Frankfurt am Main, Germany; 2 Medical Faculty, Dr. Senckenberg Institute of Pathology, Goethe University, Frankfurt am Main, Germany; 3 Max Planck Institute for Molecular Genetics, Berlin, Germany; 4 Center for the Investigation and Rehabilitation of Hereditary Ataxias, Holguín, Cuba

**Keywords:** Biomarkers, Polyglutamine, Proteostasis, SERPINB1, Spinocerebellar ataxias, Molecular biology, Neuroscience, Biomarkers, Diseases, Molecular medicine, Pathogenesis

## Abstract

Spinocerebellar ataxia type 2 (SCA2) is a polyglutamine disorder, and variants in its disease protein Ataxin-2 act as modifiers in the progression of Amyotrophic Lateral Sclerosis. There are no reliable molecular biomarkers for SCA2. The aim of this study was to define novel molecular biomarker candidates for SCA2. Using cerebellar and cervicothoracic spinal cord RNA/protein from *Atxn2*-CAG100-KnockIn (KIN) and wildtype mice, a multi-omics study was conducted based on the integration of global transcriptomic, proteomic, and phosphoproteomic data, followed by validation in mice and humans. Venn diagram comparisons across all OMICS datasets indicated that only *Serpinb1a*-transcript, SERPINB1A-protein and -phosphopeptides were consistently downregulated at terminal stage in 14-month-old KIN mice. Expression studies in cerebellum and spinal cord from 10 weeks (pre-manifest), 6-month-old (early ataxic), and 14-month-old (late ataxic stage) mice confirmed this progressive decrease at mRNA and protein level. SERPINB1 plasma levels were significantly lower in early-stage SCA2 patients, and displayed a significant association with the CAG repeat length at expanded *ATXN2* alleles, the age at onset and INAS count. However, these human data from this SCA2 founder population were not robust, so reappraisal in large international studies and at later disease stages of SCA2 is needed. SERPINB1 was identified as novel candidate progression biomarker for SCA2 pathomechanisms.

**Supplementary Information:**

The online version contains supplementary material available at 10.1038/s41598-025-29070-7.

## Introduction

Spinocerebellar ataxia type 2 (SCA2) (MIM: 183090) is a progressive neurodegenerative disorder for which there are no disease-modifying treatments. It is caused by a CAG repeat expansion mutation in the coding region of the *ATXN2* gene (cytogenetic band: 12q24.12)^1–3^. CAG repeat expansions beyond 31 CAG repeats cause the typical SCA2 cerebellar syndrome, which presents with gait ataxia, dysdiadochokinesia, dysmetria, and dysarthria. This presentation is frequently accompanied by kinetic or postural tremor, leg cramps, decreased tendon reflexes, abnormal eye movements with slowed saccades progressing to nuclear ophthalmoplegia, with reductions of body mass index and survival^4–8^. In contrast, *ATXN2* intermediate-length repeat expansions in the range of 27 to 33 units act within oligo- or polygenic backgrounds to increase the disease risk or progression of amyotrophic lateral sclerosis (ALS)^9–11^, frontotemporal dementia (FTD)^12,13^, progressive supranuclear palsy (PSP)^14^, and idiopathic levodopa-responsive Parkinson’s disease^15^.

The *ATXN2* gene encodes Ataxin-2, a cytoplasmic RNA binding factor involved in the regulation of cytoskeletal dynamics, endocytosis, growth and cellular stress responses, mRNA translation, and the inhibition of the mechanistic target of rapamycin complex 1 (mTORC1) signaling^16–23^. Upon the unstable expansion of its polyQ domain, the Ataxin-2 protein acquires a toxic gain of function leading to a multi-level atrophy, prominently of neurons but also central nervous system oligodendrocytes and presumably peripheral adipocytes^7,24–32^.

Though there is no curative treatment for SCA2 yet, Ataxin-2 abundance-lowering strategies, based on small chemical compounds^33–35^, antisense oligonucleotides^36,37^ or Cas13 CRISPR effectors^38^ against *Atxn2* mRNA, and also on pharmacological autophagy enhancement^39^, proved successful in experiments conducted in murine SCA2/ALS models. Clinical trials based on those findings have been initiated. Even so, the precise assessment of experimental therapeutic interventions relies upon informative biomarkers of disease severity and progression, with a strong involvement in the pathophysiology of SCA2. Consequently, the identification of molecular events that serve as reliable biomarker surrogate endpoints is of chief significance for the development and validation of effective treatments against SCA2.

The size of the CAG repeat expansion in Ataxin-2 predicts the disease severity and progression, but its mosaicism between cells limits the correlation between cerebellar pathology and blood genotypes. PolyQ-expanded Ataxin-2 abundance in biofluids would be an obvious candidate biomarker for SCA2, similar to what was described for huntingtin or Ataxin-3 proteins in Huntington´s disease (HD) or SCA3, respectively^40–42^. However, Ataxin-2 levels in the blood and cerebrospinal fluid are very low (https://gtexportal.org/home/gene/ ENSG00000204842.14)^43^, and no routine assay sensitive enough to consistently detect it in these biofluids has been developed yet^44^. Nonetheless, the constitutive and ubiquitous expression pattern of Ataxin-2 increases the chance of finding easily detectable peripheral molecular biomarkers, resulting from downstream effects of the gain/loss of polyQ-expanded Ataxin-2 functions. Preliminary evidence based on studies conducted in SCA2 patients suggested the levels of the mitochondrial serine/threonine protein kinase PINK1^45^, and the aggrephagy marker WDFY3^46^, as candidate blood molecular biomarkers for SCA2. Besides, trace elements (Mn, Cu, Zn, and V)^47^, oxidative stress markers^48^, and the serum testosterone levels^29^, were proposed as candidate peripheral biomarkers for SCA2. However, most of these studies focused on biological indicators of disease progression that are far downstream and irrelevant for causal therapy. Furthermore, they were conducted on small cohorts or lack independent validations, so further efforts are needed to determine or verify their clinical validity and utility.

More recently, the neurofilament light chain (NfL) levels in cerebrospinal fluid (CSF) or blood were proposed as a candidate biomarker in SCA2. In presymptomatic mutation carriers and SCA2 patients there is a substantial increase of NfL, which correlates with markers of disease severity^49–53^. Likewise, elevated NfL levels resulting from neuroaxonal damage were recognized in several neurologic disorders including ALS^54,55^, HD^56^, and SCA1, 3 and 7^52,57^, among others. Based on those findings, NfL levels have been used as a biomarker endpoint in clinical trials assessing the effectiveness of antisense oligonucleotides targeting *SOD1* mRNA (Tofersen) in ALS^58,59^, or targeting *HTT* mRNA (Tominersen) in HD^60,61^. Even though the levels of NfL in CSF/blood seem to be a promising biomarker in neurodegenerative disorders including SCA2^49,52,62,63^, its low abundance can be detected only in few centers worldwide by costly equipment, and its low specificity cannot distinguish confounders such as brain trauma / infarcts, while issues regarding assay standardization among different techniques and lab procedures still complicate its use as surrogate endpoint^64–68^.

Progress in high-throughput “omics” technologies provides new opportunities to understand the pathogenesis of neurodegenerative disorders^69,70^. Moreover, integrating several omics profiles into multi-omics platforms offers further possibilities to obtain useful insights into the mechanisms underlying the pathophysiology of these disorders, identify potential therapeutic targets, and describe relevant peripheral disease biomarkers. The use of multi-omics proved useful in biomarker research for common neurodegenerative disorders such as Alzheimer´s^71^ and Parkinson´s diseases^72^, and for rare disorders, including ALS^73^, and HD^74^.

In the present study, a multi-omics approach was applied to an authentic SCA2 mouse model, the *Atxn2*-CAG100-KnockIn (KIN)^25,28,44,75–81^, which is exceptional in capturing both the initial partial loss-of-function (with impaired transcription and translation of Ataxin-2, leading to weight gain) and the subsequent progressive toxic gain-of-function (with polyQ-expanded Ataxin-2 accumulation in aggregates, leading to weight loss and neural atrophy) that characterize all human polyQ diseases. Remarkably, previous publications on this mouse model^4,6,25,28,32,75,76,82,83^ show that it closely mirrors the selective vulnerability of neurons and neural projections, the aggregating proteins, the neurochemical profiles of lipids and N-acetylaspartate, the demyelination, and the time course of weight anomalies as they are documented in SCA2 patients (Table S1). Given that primate models of SCA2 or frozen brain samples from SCA2 patients, particularly at early stages of disease, are unavailable, our approach is expected to yield faithful insights, albeit at a high cost in finances and time.

This study revealed key pathways and molecular events linked to SCA2 pathophysiology and defined SERPINB1, a member of the SERPIN superfamily of protease inhibitors and a prime regulator of cellular proteostasis, as a candidate biomarker for SCA2. This finding was followed by validation studies in the *Atxn2*-CAG100-KIN mouse model, and in SCA2 patients from the largest and genetically homogenous SCA2 population worldwide.

## Results

**Fig. 1:**
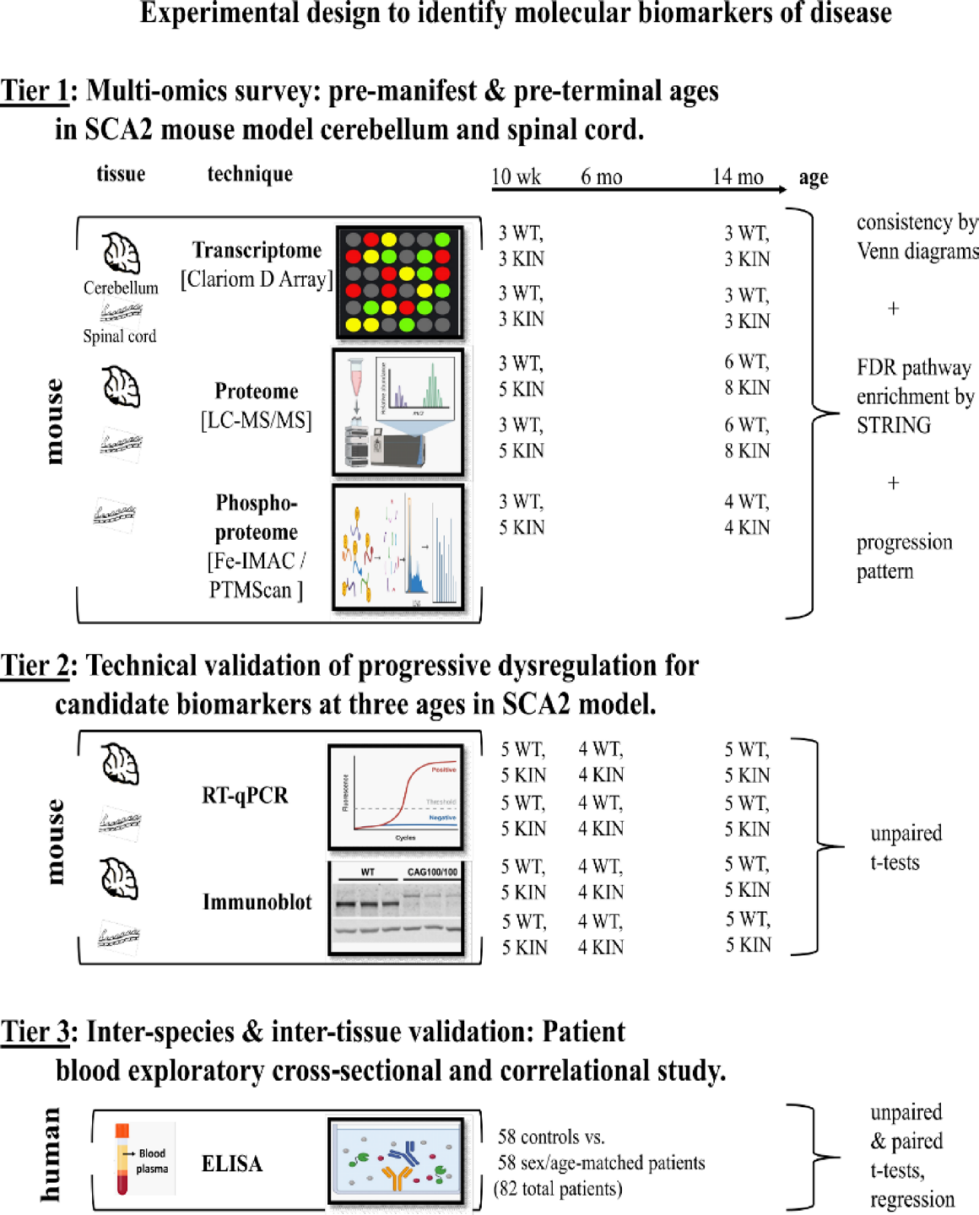
Diagram on the experimental design to identify SCA2 molecular biomarkers. Created in Biorender (https://BioRender.com).

The experimental design of this project to identify biomarkers of SCA2, consisting of an exploratory tier in our mouse model with three levels of global omics surveys (at two ages in order to assess the progression of dysregulation in the two prominently affected areas of the nervous system), followed by a validation tier in mouse with independent techniques to assess longitudinal changes, and a final tier in patient blood to assess the usefulness of candidate biomarkers as a cross-section exploratory analysis, is shown in Fig. 1.

### Transcripts encoding translation factors are predominantly upregulated since early age

In light of the relevance of Ataxin-2 as a translation regulation factor^17,18,84,85^, we first explored the cerebellar and spinal cord transcriptomes of pre-manifest and end-stage KIN mice looking for substantial dysregulations in translation factors. A list of 720 factors was obtained by filtering the transcriptome dataset in the Transcriptome Analysis Console for protein translation-related factors. In addition, a list of 505 factors that defines an up-to-date manually curated catalog of factors involved in translation was obtained from the Proteostasis Consortium (https://www.proteostasisconsortium.com/pn-annotation; last accessed on February 4, 2025).

Only *Eif5a2* transcript downregulation was consistent among the four panels of Figure S1, but reflects progression only in spinal cord and poorly (Table S2, Table S3). The upregulation of ribosomal factors is reminiscent of the *Atxn2*-KO effect^84^.

Major progressive increases in transcript expression levels involved several pseudogenes for ribosomal proteins (*Rps25-ps1*,* Rpl27-ps3*,* Rpl30-ps2*,* Rps12-ps23*,* Rpl10a-ps1*) and for the Eukaryotic Translation Elongation Factor 1 Alpha 1 (*predicted gene 6548; Gm6548*), with FC diff. ranging from 0.53 for *Rps25-ps1* to 0.79 for *Rpl10a-ps1*. Furthermore, transcripts encoding the Poly(A)-Binding Protein Cytoplasmic 1 (*Pabpc1*, FC diff. 0.65), and the Eukaryotic Translation Initiation factors 2 Alpha Kinase 2 (*Eif2ak2*, FC diff. 0.64), and 2 subunit 3, Y-linked *(Eif2s3y*, FC diff. 0.69), showed progressive upregulations. Conversely, mRNAs encoding the Eukaryotic Translation Initiation factors 5A2 (*Eif5a2*, FC diff. -0.59), and the UTP14B Small Subunit Processome Component (*Utp14b*, FC diff. -0.88), showed major progressive decreases in their expression levels (Table S3).

### Stress granule and RNA processing transcript dysregulations are prominent since early age

Given the relevance of Ataxin-2 as an RNA processing factor and as a component of stress granules^22,86–88^, we next surveyed additional stress granule and RNA processing factors as a strategy to define the relative contributions of these cellular processes to SCA2 pathophysiology. A comprehensive list of 967 RNA processing (GO: 0006396) factors was obtained from the Mouse Genome Informatics (MGI) resource (https://www.informatics.jax.org; last accessed on February 4, 2025)^89^. In addition, a list of 407 factors that defines an up-to-date manually curated catalog of stress granules proteins was obtained from the RNAgranule database (version 2.0) (http://rnagranuledb.lunenfeld.ca; last accessed on February 4, 2025)^90^.

Altogether, progressive induction of RNA processing factors was prominent in end-stage spinal cord, but no consistently altered biomarker was identified (Figure S2; Table S4).

### Transcripts encoding factors involved in the Autophagy-Lysosome pathway are induced

Because of the involvement of polyQ-expanded Ataxin-2 with the receptor-endocytosis apparatus and macroautophagy^20,32,39,91^, we examined the transcriptomes looking for dysregulated factors with prominent roles in the Autophagy-Lysosome pathway (ALP). A list of 870 factors that defines an up-to-date manually curated catalog of ALP factors was obtained from the Proteostasis Consortium (https://www.proteostasisconsortium.com/pn-annotation; last accessed on February 4, 2025).

Among all ALP factors examined, transcripts encoding lysosomal cathepsins S, Z, and L were prominently upregulated (Figure S3, Table S5). Validation efforts by quantitative reverse-transcriptase real-time PCR confirmed major upregulations of cathepsin-encoding transcripts in the cerebellum and spinal cord of end-stage KIN mice (Figure S4). Only the *Kif5b* transcript showed considerably downregulated expression levels in cerebellar and spinal cord tissues (Figure S3).

Overall, the autophagy-lysosome pathway is progressively induced in both affected tissues, but *Kif5b* as single consistently altered component did not show very strong decreases.

### Kallikrein 6 (Klk6) is prominently downregulated since early age

Interestingly, inspection of factors involved in cellular proteolytic breakdown in addition to cathepsins allowed identification of *Klk6* transcripts as progressively downregulated since the pre-manifest stage in cerebellar and spinal cord tissue of KIN mice. Cerebellar transcriptomes revealed a -2.11 and − 3.33−fold reduced *Klk6* expression levels in the pre-manifest and end-stage mice, respectively. Even more pronounced *Klk6* downregulations were found in the spinal cord, with fold changes of -4.03 and − 23.08 in the pre-manifest and end-stage mice, respectively.

Since *Klk6* was the only member of the kallikrein-related serine proteases family showing consistent and massive dysregulations across nervous tissues and disease stages, validation studies were conducted at the transcript level by RT-qPCR and at the protein level by quantitative immunoblots in five WT vs. five KIN mice (Figure S5). This confirmed the progressive significant downregulation of *Klk6* transcript, with reductions to 58.4% in the pre-manifest, 26.6% in the early ataxic stage, and 12.1% in the end-stage KIN mice cerebellum. Similarly, reductions of *Klk6* transcript expression levels to 38.3% in the pre-manifest, 22.3% in the early ataxic stage, and 21.2% in the end-stage KIN mice spinal cord were observed. Quantitative immunoblots produced similar results at the protein level in end-stage KIN mice, with reduced KLK6 abundance to 27.5% in the cerebellum and 32.1% in the spinal cord (Figures S5, S14). Altogether, these results supported KLK6/*Klk6* as a candidate progression biomarker for SCA2.

### Multi-omics data analysis define commonly dysregulated factors

After transcriptome profiling, we proceeded to integrate analyses of our transcriptomic, proteomic, and phosphoproteomic datasets. Given that cells use signal amplification cascades, events that are far downstream may appear prominent in omics fold-changes and significance levels, so we avoided such rankings. Instead, we attempted to identify upstream events that occur early-on, based on consistency criteria. We initially conducted Venn diagram analyses to define the factors in common, with nominally significant dysregulations, among the cerebellar and spinal cord transcriptomes and cerebellar proteome of pre-manifest KIN mice. As a result, no upregulated factor with nominal significance and a ≥1.5−fold change was shared across all three datasets. There were three factors commonly downregulated across all omics datasets: *Serpinb1a* coding for the proteinase inhibitor Serpin Family B Member 1 (also known as Leukocyte Elastase Inhibitor), *Mobp* encoding the Myelin Associated Oligodendrocyte Basic Protein, and *Sema7a* which codes for a member of the Semaphorin family of proteins involved in dendrite growth regulation and recently implicated in ataxia^92^ (Figure S6A/B, Table S6).

No upregulated factor was shared across all five datasets of end-stage KIN mice (Figure S6C, Table S6). STRING analysis of the 17 factors commonly upregulated in at least three out of five datasets produced significant enrichments for “Positive regulation of neuron projection development” (GO: 0010976, FDR 0.0119) and “Lysosome” (GOCC: 0005764, FDR 0.0083). On the other hand, only SERPINB1A/*Serpinb1a* was commonly downregulated across all spinal cord omics datasets (Figure S6D, Table S6). STRING analysis of the 26 factors commonly downregulated in at least three of five datasets produced significant enrichments for “Synapse” (GO: 0045202, FDR 0.009), among others.

Jointly, consistency analyses across transcriptome, proteome and phosphoproteome profiles with progression from pre-manifest to end-stage identified SERPINB1A as single most promising progression biomarker candidate, with massive downregulations.

### Omics datasets provide evidence for substantial dysregulations among several members of the serpin superfamily of protease inhibitors

SERPINB1A belongs to the serpin superfamily of protease inhibitors that target cathepsins and kallikrein-related peptidases. Given that cathepsins showed prominent upregulations in the cerebellar and spinal cord transcriptomes of end-stage KIN mice and that there is a progressive downregulation of *Klk6* since early age, we wondered if additional serpin family members showed dysregulations that may explain protease expression level/abundance changes resulting from Ataxin-2 polyQ-expansion.

Detailed examination of the cerebellar and spinal cord transcriptomes of 10-week-old and 14-month-old KIN mice revealed several dysregulated serpins reaching nominal significance in addition to *Serpinb1a*. Most of these serpins were downregulated in every tissue and age group examined (Figure S7; Table S7).

In experiments involving five mutant *versus* five WT samples, major serpin transcriptional dysregulations, apart from *Serpinb1a*, were validated by RT-qPCR in cerebellar and spinal cord tissues from pre-manifest and end-stage KIN mice (Figure S8).

Overall, the multi-omics data are compatible with the hypothesis that the dysregulations of serpins (prominently *Serpinb1a*) reflect severe, early and progressive proteostasis issues, which may be revealing compensatory cellular efforts to promote the efficient degradation of polyQ-expanded Ataxin-2 aggregates by suppressing inhibitory actions of serpins on the activity of relevant proteases.

### SERPINB1A protein and Serpinb1a transcript are consistently downregulated

*Serpinb1a* transcript showed − 2.20 and − 3.47−fold reduced expression levels in the cerebellar and spinal cord transcriptomes of pre-manifest 10-week-old KIN mice, and expression level fold changes of -2.36 and − 3.03 in the cerebellar and spinal cord transcriptomes of end-stage KIN mice. Notably, results from the cerebellar proteome of pre-manifest KIN mice showed a significantly 1.96−fold (*p* = 0.0002) reduced abundance for SERPINB1A, and similar decreases were apparent in the cerebellar (FC 3.73, *p* = 0.0003) and spinal cord (FC 2.06, *p* = 0.0001) proteomes of end-stage mice. Besides, two significant hypophosphorylations emerged in the spinal cord phosphoproteome (4.1−fold at Ser62, 5.1−fold at Ser300), probably corresponding to the reduced protein abundance of SERPINB1A.

**Fig. 2:**
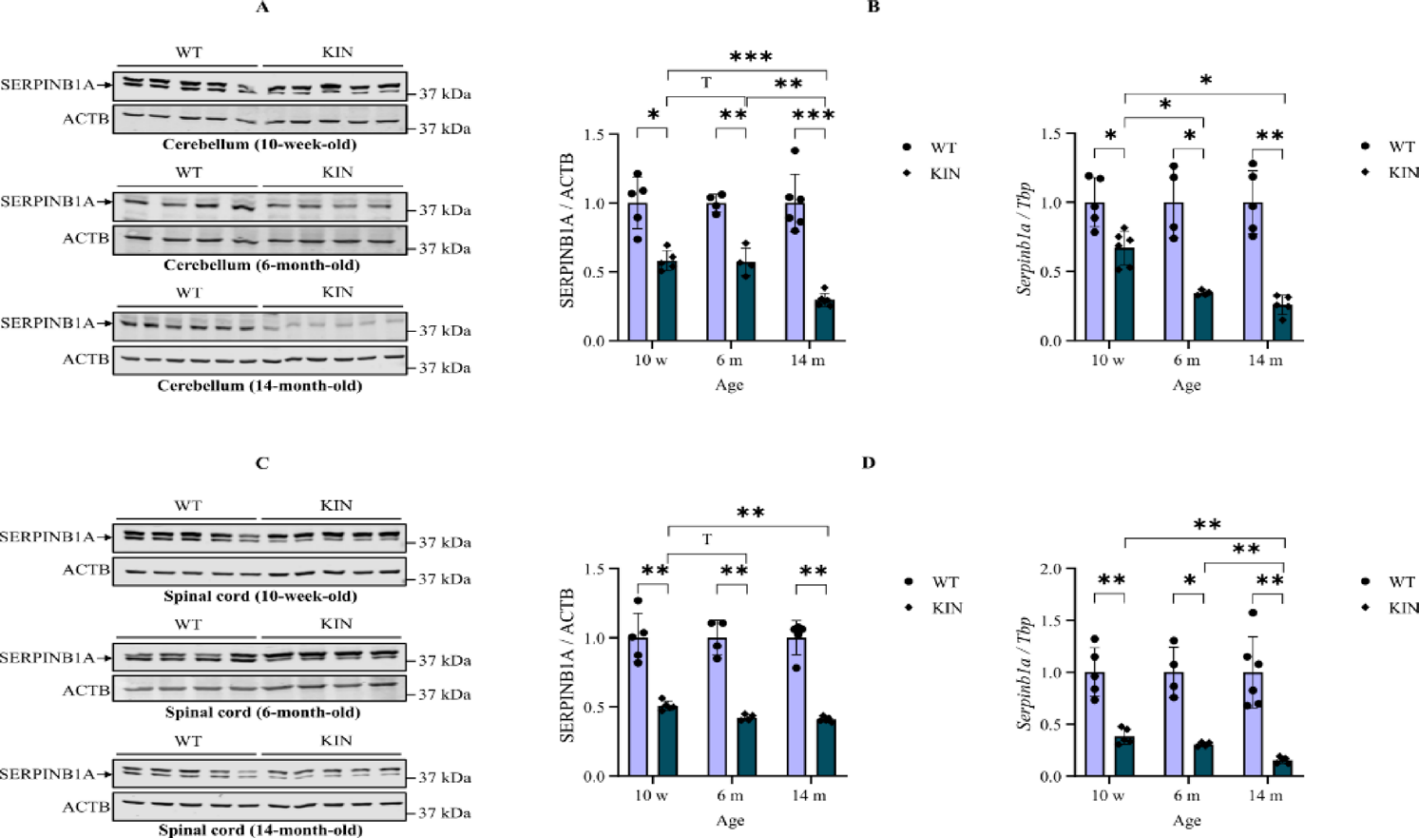
Quantitative immunoblots (**A**,** C**) and quantitative reverse-transcriptase real-time PCR (**B**,** D**) showing consistent downregulation of SERPINB1A/*Serpinb1a* in the cerebellum (**A**,** B**) and spinal cord (**C**,** D**) of 10-week-old (pre-manifest), six-month-old (early ataxic stage), and 14-month-old (late ataxic stage) *Atxn2*-CAG100-KIN mice. w = week, m = month. The blots were cropped and full-length raw blots for all data in this study are provided in Figure S14 and Figure S15. Error bars are depicted as ± SEM. T < 0.10; *p < 0.05; ** *p* < 0.01, *** *p* < 0.001.

Validation efforts at the transcript level by RT-qPCR and the protein level by quantitative immunoblots confirmed the progressive reduction in *Serpinb1a* transcript and SERPINB1A protein abundance in the cerebellum and spinal cord from 10-week-old (pre-manifest), six-month-old (early ataxic stage), and 14-month-old (late ataxic stage) KIN mice (Fig. 2). Quantitative immunoblots in nervous tissue detected isoform bands migrating around molecular weights of 42, 39 and 25 kDa, in excellent agreement with UniProt database entries P30740-1 / B4E3A8 / P30740-2. In the cerebellum, SERPINB1A protein abundance reductions to 58.2, 57.2, and 29.7% were apparent in the pre-manifest, early, and late ataxic disease stages, respectively. Corresponding numbers at the transcript level were 66.9, 34.5, and 25.8%, respectively. Similarly, reductions of SERPINB1A protein abundance in the spinal cord of pre-manifest, early, and late ataxic stage mice were 50.8, 42.3, and 40.9%, respectively. Likewise, *Serpinb1a* transcript assessed at the 3’ end exon 6–7 junction was reduced in the spinal cord to 38.2% in the pre-manifest, 30.6% in the early ataxic stage, and 15.4% in the late ataxic stage KIN mice.

### SERPINB1 plasma levels were reduced, correlating with severity markers, in patients from the SCA2 founder population in Cuba

Further validation efforts were conducted in 58 patients (male/female: 25/33) with clinical and molecular diagnosis of SCA2 from the founder population in Holguín/Cuba, and in an equal number of healthy individuals from the same geographic area (male/female: 19/39). In the SCA2 patients group, age varied between 26 and 76 years, with a mean (SD) of 51.12 (13.09) years, and the expansion ranged between CAG34 and CAG48 (Figure S9), with most patients in the mild stage and only very few in the severe stage (Figure S10). Meanwhile, among healthy control individuals, the age ranged between 26 and 76 years, with a mean (SD) of 49.48 (12.53) years. No significant differences were observed between SCA2 patients and controls regarding sex (χ^2^ = 1.318; *p* = 0.251) and age distributions (t=-0.688; *p* = 0.493) (Table S8). Most patients were in disease stage 1 (mild ataxia) (62.07%), followed in frequency by patients with moderate ataxia (disease stage 2) (29.31%), so any molecular progression biomarker changes would be expected to be relatively mild at this stage.

**Fig. 3:**
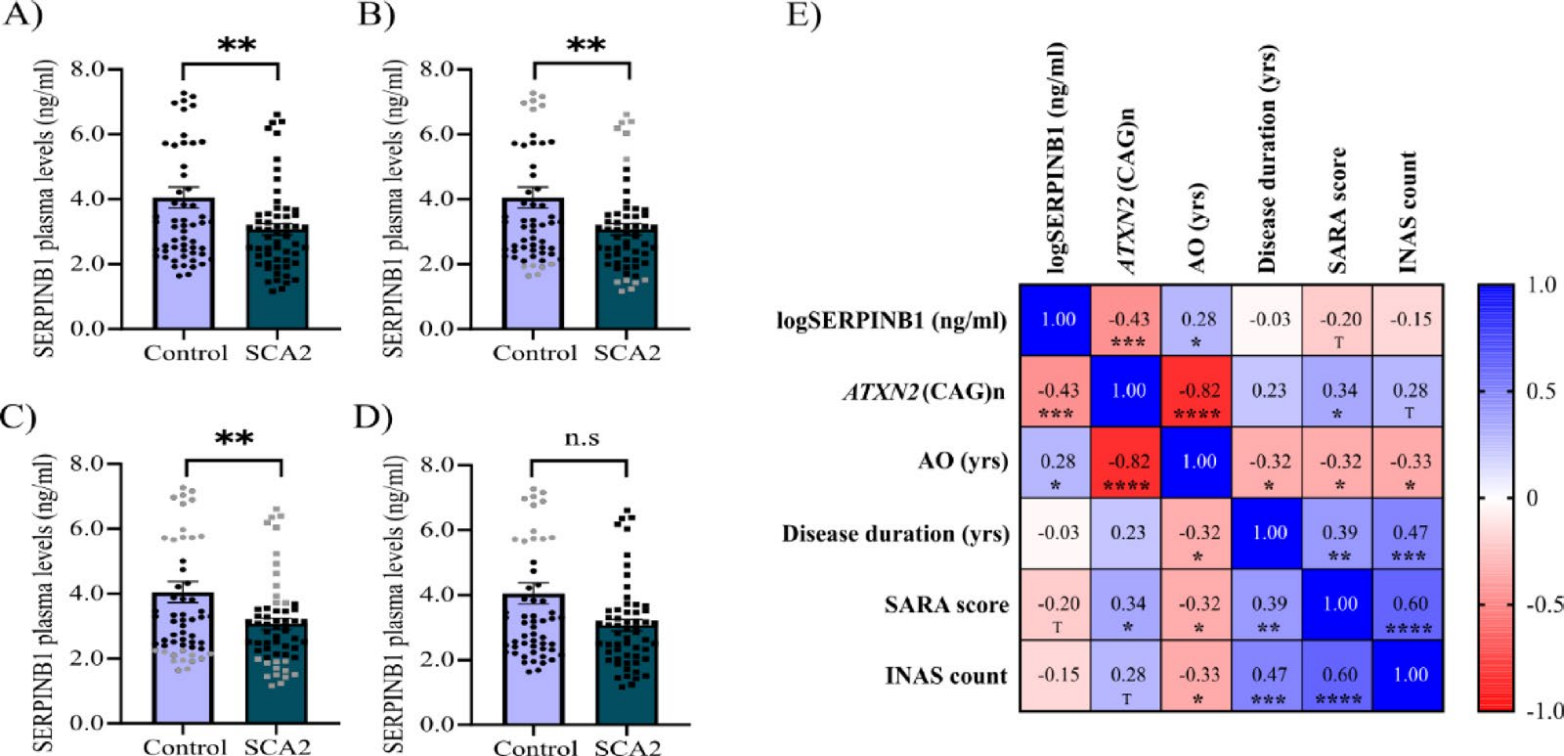
SERPINB1 plasma levels in SCA2 patients and healthy control individuals. (**A**) Mean comparisons for SERPINB1 plasma levels between patients (*n* = 58) and age and sex matched (*n* = 58) control individuals. (**B-D**) Trimmed mean analyses for SERPINB1 plasma levels in SCA2 patients and control individuals for symmetric 10% (**B**), 20% (**C**), and asymmetric (**D**) trimmed means in the data set of sex- and age-matched 58 patients with SCA2 and 58 control individuals. (**E**) Correlation matrix for the log-transformed SERPINB1 plasma levels and disease severity markers in SCA2 patients (*n* = 82). Error bars are depicted as ± SEM. *p < 0.05; ** *p* < 0.01, *** *p* < 0.001, **** *p* < 0.0001; T− trend (0.10 > *p* > 0.05).

SERPINB1 blood plasma levels varied between 1.163 and 7.273 ng/ml in the studied sample, with a mean (SD) of 3.561 (2.032). Since SERPINB1 did not follow a normal distribution (K-S = 0.140; *p* < 0.001), it was log-transformed for hypothesis testing, then rendering an approximately normal distribution (K-S = 0.077; *p* = 0.097). Based on this, in unpaired t-tests no significant differences were found for SERPINB1 plasma levels across sexes (t=-0.091; *p* = 0.927), and it showed a modest correlation with age (*r* = 0.189; *p* = 0.042). As a major finding, a nominally significant 17.88% reduction in SERPINB1 plasma levels was obtained for SCA2 patients relative to control individuals (t = 2.618; *p* = 0.010), with mean (SD) values of 3.070 (1.342) *versus* 4.051 (2.459) for patients and controls, respectively (Fig. 3A).

An additional analysis was performed by paired t-test of perfectly sex- and age-matched (± 2 years) 49 patients (male/female: 19/30) and 49 healthy controls (male/female: 19/30). Patient age ranged between 26 and 76 years, with a mean (SD) of 50.55 (12.69) years, while control age ranged between 26 and 76 years, with a mean (SD) of 50.55 (12.61) years. Again a significant downregulation of SERPINB1 was found (t = 2.609; *p* = 0.012), with mean (SD) values of 3.116 (1.407) versus 4.152 (2.462) for patients and controls, respectively. Analyses with symmetric trimmed means and non-parametric unpaired/paired statistics also supported this finding. However, statistical significance was lost after asymmetric trimming of the high-value subcluster (> 5.5 ng/mL) in the control group. Additional analyses using a receiver operating characteristic curve in each cohort revealed significant differences in the area under the curve (*p* = 0.0413), with no cutoff values for SERPINB1 plasma levels demonstrating high sensitivity and specificity simultaneously. ANCOVA results confirmed a significant difference between SCA2 patients and controls, which disappeared after controlling for the high-value subcluster in the control group (Table S9). Visual inspection of connected dot plots for the 58 vs. 58 and 49 vs. 49 datasets indicates a similar overall variability between patients and control individuals, with predominance of decreasing slopes from controls to patients, and clusters of lines with the more pronounced slopes and diverging patterns involving control individuals with more than 5.5 ng/mL (Figure S11).

The relevance of SERPINB1 plasma levels for disease severity was assessed in an extended sample of 82 SCA2 patients by linear correlation and regression analyses. Baseline characteristics for this extended cohort are shown in Table S10. Similar to the case-control cohort, most patients in the correlational study were in disease stage 1 (72.86%), followed in frequency by patients in disease stage 2 (17.14%), and there were no significant effects for sex (t=-1.339; *p* = 0.185) or age (*r* = 0.175; *p* = 0.115) on SERPINB1 levels. As expected, significant correlations were found between the CAG repeat length at expanded *ATXN2* alleles and clinical severity markers, including the age at onset and the SARA score (Figure S12A, showing also corresponding scatter plots with regression lines and Q-Q plots for the SCA2 patients). Remarkably, SERPINB1 plasma levels were significantly correlated with the CAG repeat length and the age at onset (Fig. 3B).

Case-wise diagnostics, for residuals after simple linear regression of SERPINB1 plasma levels on markers of disease severity, allowed us to identify two outliers for the age at onset and INAS count, three for the SARA score, and one for disease duration (Figure S12B, including corresponding scatter plots with regression lines and Q-Q plots). Correlation analyses after outliers removal produced significant results for the age at onset (*r* = 0.393; *p* = 0.004), and INAS count (*r*=-0.307; *p* = 0.013). The influence of SERPINB1 plasma levels on the age at onset remained significant after adjusting it to the CAG repeat length by linear regression (β(SE) = -1.553 (0.710); t=-2.187; *p* = 0.034; R^2^_adj_ = 0.667). Linear regression analyses indicated that 2.6% of the total variability in the age at onset might be attributable to the variations in SERPINB1 plasma levels. Thus, beyond the CAG expansion effect as main confounder, some 7.24% of the unaccounted variation in age at onset could be reflected by variation at SERPINB1 plasma levels. While such an impact on onset variance is bigger than most effects of genetic modifier polymorphisms in genome-wide association studies of neurodegenerative diseases, the finding should be treated with caution until confirmed independently.

Besides, a trend was observed for decreasing SERPINB1 plasma levels along with advancing disease stages, from mild (mean: 4.179 ng/ml), to moderate (mean: 3.886 ng/ml), to severe ataxia (mean: 2.593 ng/ml), though not statistically significant (F = 1.544; *p* = 0.221) (Figure S13). Further attempts to stratify patients by disease stage versus the expansion size generated subgroups of insufficient statistical power both in parametric and non-parametric tests.

Efforts to detect KLK6 in these blood samples by the EKLK6 ELISA kit (Invitrogen) were not successful.

## Discussion

The search for reliable and informative biomarkers is crucial for assessing the clinical utility of experimental disease-modifying treatments for SCA2. Current clinical trials use ataxia scores, cerebellar imaging, nerve conductance, perhaps the quantitation of NfL as axonal damage marker, and ATXN2 to assess efficacy of disease protein elimination, but molecular biomarkers in the affected pathways are lacking. Recent transcriptome/proteome/lipidome profiling in nervous tissues from SCA2 mouse models, which mirror disease hallmarks, has revealed the crucial pathomechanisms^25,28,32,44,75–81,93–95^. In particular, the prominent cerebellar and spinal cord pathology in our KIN SCA2 mouse model closely resembles findings in patients with SCA2 in terms of atrophy and neuronal loss/disconnection, neuroinflammation with microgliosis and astrogliosis, TDP-43 mislocalization and aggregation, demyelination and early sensory axonal neuropathy^9,25,28,32,76,96–101^, stressing its relevance for translational research. Perhaps most importantly, it revealed how the toxic gain-of-function may be mediated by accumulation of N-terminal fragments from the first and second translation start codon (including the polyQ domain) and a conjoint activation of the selective aggrephagy receptor Sequestosome-1, while the partial loss-of-function of ATXN2 may be mediated by the several-fold decrease in abundance from the third start codon before LSM until the C-terminus^23^.

As the novel contribution of the present study, we now explored the temporal evolution from pre-manifest to end-stage KIN mice for transcripts involved in these processes, to define mechanistic disease progression biomarkers. Integrating multi-dimensional transcriptomic profiles from cerebellar and spinal cord tissue at two ages with proteomic and phosphoproteomic surveys, we looked for consistency across datasets. As the main finding, the mouse data define SERPINB1 downregulation in transcript, protein and phosphorylation assays as the most promising candidate progression biomarker for SCA2. Although SERPINB1 is mainly expressed in peripheral immune cells rather than in the nervous system under physiological conditions, it is unlikely that SERPINB1 downregulation responds to peripheral effects of ATXN2 polyQ expansion, given that the affection of peripheral tissues in SCA2 is relatively scarce and fails to progress over time (with exception of the loss of weight and fat in particular, features that are unspecific and occur in most neurodegenerative disorders). Thus, a cross-compartmental mechanism should be taken into consideration.

Changes in *Serpinb1a* transcript levels and SERPINB1A protein abundance were verified by RT-qPCR and immunoblots in the relevant mouse tissues, confirming their gradual decrease along with disease progression. Furthermore, using a sandwich enzyme-linked immunosorbent assay as an additional technique, a significant but non-robust overall reduction in blood plasma SERPINB1 abundance was observed in SCA2 patients already at early stages, though data heterogeneity reinforces the need for larger datasets. Remarkably, blood plasma SERPINB1 abundance was significantly associated with the CAG repeat length at expanded *ATXN2* alleles, the age at disease onset and the INAS count, so lower SERPINB1 levels appeared to correlate with longer repeats, earlier onset, and higher INAS count as an indication of increased disease severity. However, lack of significant correlations with the disease duration and the SARA score indicated that further studies in larger and longitudinal cohorts from different SCA2 populations, and particularly follow-up studies including late disease stages, are crucial to sort out uncertain progression trajectories.

SERPINB1 belongs to the clade B or ov-serpin subfamily of the SERPIN (serine or cysteine protease inhibitor) superfamily^102^. It is needed to limit the excessive tissue damage during inflammatory responses to microbial infections by repressing the activity of elastase-like and chymotrypsin-like serine proteases and restricting the activity of inflammatory caspases^103–105^. Besides, SERPINB1 may influence lysosomal pathways during immune responses^106^. Although the functions of SERPINB1 in the nervous system have not been extensively studied, *SERPINB1* transcripts were found dysregulated in sporadic Creutzfeldt-Jakob disease and Alzheimer’s disease (AD) patients^107^. According to genome-wide association studies of AD risk, genetic variants of SERPINB1 act as progression modifiers, and higher transcript levels of SERPINB1 in prefrontal cortex show direct correlation with increased amyloid Abeta 42 in cerebrospinal fluid^108^. Furthermore, SERPINB1 was identified as a novel therapeutic target in a recent study using Mendelian randomization to repurpose licensed drugs for AD^109^. These observations indicate that SERPINB1 exerts roles that are relevant for nervous system proteostasis and that its alteration is associated with neurodegenerative processes.

Although its overall expression levels in healthy brain are relatively low compared to other tissues, SERPINB1 is enriched in microglia, which is consistent with its role in inhibiting protease activity during inflammatory responses. Based on these observations, we speculate that the progressive decreased abundance of SERPINB1 in the cerebellum and spinal cord of KIN mice may indicate a state of dysregulated proteostasis, with de-repression of phagocytosis and lysosomal pathways in microglia as part of an innate immune response to neuron−derived polyQ-expanded Ataxin-2 aggregates, intended to limit cell-to-cell transfer of the aggregates and the spreading of pathology.

In line with this hypothesis, transcriptome profiling produced evidence of profuse downregulations in various *Serpins*, in parallel to upregulations of lysosomal cathepsin, in the cerebellum and spinal cord of KIN mice, partially already at early-stage disease. Given that the serpin superfamily of protease inhibitors primarily evolved as a buffering system to avoid excessive protein degradation by cathepsins and kallikrein-related peptidases^104,110–113^, these expression results suggest a simultaneous induction of cathepsins and suppression of serpins is taking place. *Serpinb1a* and *Klk6* mRNAs in rat brain tissue are both distributed to the neuropil with scores similar to microglia markers *Aif1* and *Tyrobp*, according to datamining (https://public.brain.mpg.de/dashapps/localseq/explore). The human protein atlas database (https://www.proteinatlas.org/) indicates *Serpinb1* to localize to microglia, macrophages, monocytes and neutrophils, while *Klk6* was assigned to oligodendrocytes. Single-cell RNAseq data from several human and mouse brain surveys compiled in a public database (https://brainrnaseq.org/) indicate that human *Serpinb1* was present mainly in microglia, while murine *Serpinb1a* was enriched in myelinating oligodendrocytes, and both human and murine *Klk6* was found in oligodendrocytes. In keeping with the important role of the immune system in triplet repeat expansion diseases^114^, also single-nucleus RNA sequencing and spatial transcriptomics of adult human spinal cord^115^, compiled in the “Human Spinal Cord snRNAseq Viewer” (https://vmenon.shinyapps.io/humanspinalcord/), showed *Serpinb1* as enriched in homeostatic, primed, interferon-responsive, proliferating, and perivascular subpopulations of microglia and macrophages, while *Klk6* was mainly allocated to oligodendrocytes. According to the SeqSeek tool (https://seqseek.ninds.nih.gov/genes), based on single-cell RNA sequencing data from mouse spinal cord^115,116^, murine *Serpinb1a* and *Klk6* were most abundant in myelinating oligodendrocytes. These preliminary data highlight cell-type-specific abundance for both factors, which should be experimentally reexamined in the future. The precise role of both factors for neural ATXN2 degradation therefore remains to be understood. It is known already that microglia are crucial for the elimination of ATXN2 polyQ-expansion aggregates and Huntingtin inclusion bodies^76,114^. Remarkably, the transcriptional adaptation of *Serpinb1a* and *Klk6* is substantial and strong already at pre-manifest stages of our SCA2 mouse model, suggesting early dysregulation of proteostasis control, long before the ATXN2 protein aggregates become microscopically detectable in spinal cord and cerebellar neurons^25,76^. Thus, we reasoned that the progressive lowering of SERPINB1 in brain areas might become a clinical useful surrogate marker of microglial activation to prevent the formation of neuronal inclusion bodies, although the SERPINB1 amounts derived from the nervous system are small in comparison to its peripheral abundance. Similar compensatory cellular efforts may be a mechanism common to neurodegenerative proteinopathies, since dysregulations of diverse serpins and cathepsins were implicated in the pathology of several neurodegenerative disorders, including AD, PD, HD, ALS, and prion diseases^107,117–122^.

Interestingly, *Serpini1* transcript (encoding neuroserpin) showed consistent downregulations in the cerebellar and spinal cord KIN mice transcriptomes since early age, although to a lesser extent than *Serpinb1a*. Given the association of neuroserpin with neurodegenerative conditions^123,124^, and its involvement in the modulation of neurogenesis, neuronal axon growth and density of dendritic spines at synapses^125–128^, it may become another relevant candidate biomarker for SCA2.

On the other hand, transcripts encoding cathepsins D, B, and L were among the most prominently upregulated in the nervous tissue of KIN mice. These cathepsins are particularly abundant lysosomal proteases, ubiquitously expressed, and enriched in microglia^129,130^. Remarkably, the cathepsins D, B, and L, in addition to cathepsin Z, were implicated in the partial or total degradation of polyQ-expanded huntingtin^131–136^. Interestingly, the *Ctsl* transcript showed the strongest progressive upregulations among all factors examined in the *Atxn2*-CAG100-KnockIn mouse transcriptomes. Based on these observations, lysosomal cathepsins might play a role in the N-terminal proteolysis^137^ or total degradation of polyQ-expanded Ataxin-2 monomers or aggregates, with potential relevance as SCA2 biomarkers and therapeutic targets. N-terminal fragments of polyQ-expanded ATXN2 were recently shown to stand out, conjointly with the selective aggrephagy receptor Sequestosome-1, by exceptional > 100-fold hyperphosphorylations in our SCA2 KIN mouse model spinal cord at advanced age, which may simply reflect accumulation of these peptides or may serve to modulate liquid-liquid phase separation^23^. It is crucial to note that these observations indicate all RNA-processing domains in polyQ-expanded ATXN2 to be affected by a partial loss-of-function, whereas the N-terminal polyQ-expansion and its adjacent proline-rich motifs appear to mediate many neurotoxic gain-of-functions in this SCA2 model, a situation with close similarity to the pathomechanisms of polyQ-expansion triggered Huntington’s disease^138–141^.

Besides cathepsin proteases, kallikrein-related peptidases (KLK) as another family of cellular proteases under control of serpins may play a prominent role in SCA2 pathology. In particular, the *Klk6* transcript was progressively downregulated since early age. Validation studies provided additional evidence supporting a prominent reduced abundance of KLK6 protein in the cerebellum and spinal cord of end-stage *Atxn2*-CAG100-KnockIn mice. Given that KLK6 promotes the degradation of myelin basic protein and plays a role in demyelinating conditions^142–144^, apart from targeting protease−activated receptors PAR-1 and PAR-2 in response to neural damage^145^, its downregulation in our KIN mice where demyelination is prominent^32^, seems to be protective. Similar to its proposed usefulness as a biomarker for AD^146–148^, it is conceivable that KLK6 levels will be found to correlate with the widespread white matter damage in SCA2^149,150^.

Further evidence for lysosome activation in KIN mice due to polyQ-triggered protein proteostasis problems comes from the observed strong upregulation of the microglia−enriched factors *Grn*,* Trem2*, *Laptm5*, and *Nfe2l2*, along with the prominent downregulation of *Itpr1*, *Arsg*, *Lamp5*, and *Kif5b*^151–158^.

Conversely, significant upregulations of several translation factors involved in ribosome biogenesis and protein translation are reminiscent of observations in the Ataxin-2 Knock-Out mouse, thus suggesting cellular compensatory efforts in the face of an Ataxin-2 partial loss-of-function due to its mutation. Of particular interest among translation factors was the progressive induction of *Eif2ak2* in the spinal cord of KIN mice. *Eif2ak2* codes for the Eukaryotic Translation Initiation Factor 2 Alpha Kinase 2 (aka Protein Kinase R, PKR), which is a serine/threonine-specific, interferon-inducible protein kinase activated by viral or endogenous double-stranded RNA (dsRNA) and a crucial component of stress granules and the Integrated Stress Response (ISR)^159–161^. In this scenario, the induction of *Eif2ak2* in the spinal cord of our KIN mouse may reflect a cellular innate immune system response elicited by double-stranded hairpin structures in the CAG-repeat expanded *Atxn2* mRNA, as a toxic RNA-mediated gain-of-function mechanism with a persistent downstream ISR dysregulation leading to impaired synaptic function and neural atrophy.

The present study has relevant limitations, with murine data but particularly the human findings being exploratory, and with a high need of independent validation before claims about the usefulness of these candidate biomarkers can be made:

Firstly, mouse models can never completely recapitulate the processes in human diseases, but at present the desirable validation of nervous system anomalies is impossible due to the unavailability of frozen patient brain tissue in this rare disorder, and because blood samples from our SCA2 mouse model were also unavailable to us. The latter may be studied in the future, and given the fact that the polyQ-expansion in the *Atxn2*-CAG100-KnockIn can be removed in adult life via Cre/LoxP/Tamoxifen technology it will be possible to test until what stages of disease the reversibility of proteostasis issues and *Serpinb1a* dysregulation can be achieved. Sperm from our KIN mice is deposited at the European Mouse Mutant Archive (EMMA), so these experiments can be done by expert teams in the future. It is acknowledged that the small sample number whose profiling by multi-OMICS could be financed, and the lack of mathematical corrections e.g. for unbalanced group numbers, or for multiple testing before generating Venn diagrams, carry risks of false positive and false negative findings. We also concede that the physiological levels of ATXN2 protein are high in neurons but low in blood, while SERPINB1 levels are low in neurons but high in blood, with apparently unrelated functions, so any correlation between these factors is correlative at present. A causal relationship with mechanistic insights, how the ATXN2-polyQ expansion causes proteostasis problems that may trigger a downregulation of specific SERPINB1 isoforms as protease inhibitors, remains to be established in the future. It is conceivable that the SERPINB1 downregulation in SCA2 reflects a secondary inflammatory or metabolic reaction or an indirect crosstalk between the nervous system and microglial responses plus peripheral macrophage infiltration, but it is also possible that SERPINB1 represents a selective regulator of the elimination of toxic ATXN2 that serves a crucial role remarkably early in the disease course, of potential value for neuroprotective therapies. Of course, it will be desirable to validate our findings also in the nervous system of other SCA2 models and of SCA2 patients in the future, but the available samples usually represent late stages of disease with microscopically detectable neuronal inclusion bodies, whereas the most intriguing observation in our work is the extremely early occurrence of proteostasis dysregulation before aggregation becomes detectable.

Secondly, preliminary analyses of patient blood samples were limited to early stages of disease, where only minor biomarker changes can be expected. The logistics of overcoming patient immobility at late disease stages for study purposes went beyond the time-frame and budget of this project. It will be crucial in the future to assess patients at severe and pre-final stages (as in our SCA2 mouse model), and to conduct intra-individual longitudinal assessment over several years, across large patient cohorts, always with perfectly matched controls and subgroup heterogeneity tests. Our current human data are cross-sectional, with small sample size, suffer from subgroup heterogeneity, have no replication cohort, and therefore constitute non-robust exploratory findings. All association findings at present are descriptive and correlative, future mechanistic analyses remain to be carried out. The fact that a SERPINB1 high-value subgroup was disproportionally more frequent in controls than patients was at the basis of the apparently significant difference between patients and controls, and may be due to pre-analytical variability (handling, storage, metabolic or inflammatory state) reflecting biological variability unrelated to SCA2. The validation of our preliminary findings should be performed by independent researchers, and instead of the Cuban SCA2 population as genetic isolate, representative patient and control groups from across the world with extensive characterization should be used. Only such approaches will permit in depth-evaluations, where the effect of CAG-expansions on disease onset / severity / duration can be eliminated and where the added value of proteostasis markers such as SERPINB1 can be assessed, clarifying to what degree they account for the currently unexplained variability of phenotype. It is already known that ATXN2 has low abundance in various blood cell types, playing a relevant role for the development of megakaryocytes^162^. Furthermore, SERPINB1 should be quantified in the cerebro-spinal fluid in dependence on SCA2 genotypes and disease stages.

Altogether, in this study of temporal evolution in an unbiased multi-omics approach, with further validation studies in mouse nervous tissues and human blood sera, SERPINB1 was identified as the most promising novel candidate, which should be evaluated for its utility as progression biomarker for SCA2. Current exploratory data suggest that its decreased levels correlate with polyQ expansion size, age at onset, INAS count, and show progressive reduction over time. However, mechanistic assessments in additional experimental models for SCA2 and replication of main findings in different SCA2 populations remain to be done in the future.

## Methods

### Study design

An initial transcriptome profiling of translation, stress granules, RNA processing, and autophago-lysosome pathway factors was followed by an unbiased multi-omics study, based on the integration of transcriptomic, proteomic and phosphoproteomic data obtained in cerebellar and spinal cord tissue from pre-manifest (10-week-old) and end-stage (14-month-old) *Atxn2*-CAG100-KnockIn (KIN), and age and sex-matched wild type (WT) mice. The KIN mouse is an authentic model for SCA2, faithfully mirroring hallmarks of SCA2 pathology^25,28,75,76^. Validation studies were performed at transcript and protein levels in cerebellar and spinal cord tissue from pre-manifest, early- (6-month-old), and end-stage KIN mice. Further validation studies were conducted in 58 patients with SCA2 and equal number of age and sex-matched healthy individuals from the same geographical region as the patients. The SCA2 patients’ cohort was enlarged to 82 individuals for correlational analyses considering SERPINB1 blood plasma levels and core markers of disease severity.

### Experimental animals

All mouse experiments were in conformity with the German Animal Welfare Act, the Council Directive of 24th November 1986 (86/609/EWG) with Annex II, and the ETS123 (European Convention for the Protection of Vertebrate Animals). The Regierungspräsidium Darmstadt, with approval number V54-19c20/15-FK/1083, ethically supported the study. All procedures were carried out in strict compliance with all applicable guidelines and regulations, and reported in accordance with ARRIVE guidelines.

All mice were obtained from Charles River Germany, housed at the Central Animal Facility (ZFE) of Goethe University Medical School, kept in individually ventilated cages with nesting material at a 12 h light/12 h dark cycle, with appropriate temperature and humidity conditions, and provided with food and water *ad libitum*. Among offspring littermates, the homozygous KIN and WT animals of the same sex were selected and aged together in the same cages, for subsequent experimental group comparisons regarding gene expression, protein, and phospho-peptides abundance. WT and KIN mice were genotyped as previously described^25^. For experimental purposes, mice were euthanized by carbon dioxide exposure, followed by cervical dislocation. Both female and male mice were used during all experiments.

### SCA2 patients and healthy control individuals

The Ethics Committee of the Center for the Investigation and Rehabilitation of Hereditary Ataxias (CIRAH) approved the study protocol, and written informed consent was obtained from patients according to the Declaration of Helsinki. All research was performed in accordance with relevant guidelines and regulations.

All samples were obtained in fasting state, mostly in field expeditions (where body weight measuring devices were unavailable), using BD Vacutainer EDTA tubes for venous blood extraction and anticoagulation, centrifuging the samples for 15 min at 1000×g at 2–8 °C within 30 min of collection, collecting the supernatant for assays in Eppendorf Safe-Lock polypropylene 1.5 ml tubes without surface treatment, and storing un-diluted samples aliquots at -20 °C until use. The maximal storage time for plasma samples before SERPINB1 quantification was two weeks. Repeated freeze-thaw cycles were avoided. Blood plasma SERPINB1 levels were measured in randomly selected patients with a clinical and molecular diagnosis of SCA2, and in age (± 2 years) and sex-matched healthy control individuals from the same geographical region as the patients. Control individuals with family history of neurodegenerative disorders were excluded from the study. Likewise, individuals with reported personal history of lung disease, Cohen syndrome, alpha 1-antitrypsin deficiency, impetigo, vasculitis, granulomatosis with polyangiitis, plasma protein metabolism disease, psoriasis, cystic fibrosis, systemic lupus erythematosus, or neutropenia, were also excluded based on their reported associations with altered SERPINB1 blood levels (https://www.genecards.org/cgi-bin/carddisp.pl?gene=SERPINB1&keywords=serpinb1# diseases; last accessed on December 20, 2024).

### Transcriptome profiling

Global transcriptomic studies based on total RNA obtained from cerebellar and cervicothoracic spinal cord tissue from three WT and equal number of end-stage (14-month-old) homozygous KIN mice were conducted as previously described (Canet-Pons et al., 2021). Briefly, the total RNA integrity was determined using the 2100 Bioanalyzer with the Nano Assay (Agilent Technologies, Santa Clara, CA) (RIN > 8.5). Single−stranded cDNA (ss−cDNA) was generated using the GeneChipTM WT PLUS Reagent Kit (Applied Biosystems, Foster City, CA) following the manufacturer’s instructions. The ss−cDNA was fragmented and labeled immediately before the hybridization to a Clariom D Array (Thermo Fisher Scientific). The arrays were scanned with the Affymetrix GeneChip Scanner, and the resulting data were processed with the Transcriptome Analysis Console (TAC) 4.0.1 (Applied Biosystems) using default software parameters.

### Proteome profiling

Cerebellar and spinal cord protein extracts from eight WT and six end-stage (14-month-old) homozygous KIN mice, and cerebellar protein extracts from three WT and five pre-manifest (10-week-old) homozygous KIN mice were used for proteome profiling. In short, tissue samples were homogenized under denaturing conditions, and then boiled at 95 °C for 10 min, sonicated for 10 min, and centrifuged at 16,000 rcf for 10 min at 4 °C. Supernatants were transferred into new protein low binding tubes (Eppendorf, Germany). Lysed and trypsin-digested samples were desalted over C18 columns, reconstituted in 2% formic acid in water, and separated by strong cation exchange chromatography (SCX, 3 M Purification, Meriden, CT). Eluates were first dried in a SpeedVac, then dissolved in 5% acetonitrile and 2% formic acid in water, briefly vortexed, and sonicated in a water bath for 30 s previous to injection to nano-LC-MS/MS. The LC-MS/MS was carried out by nanoflow reverse-phase liquid chromatography (Dionex Ultimate 3000, Thermo Scientific) coupled online to a Q-Exactive HF Orbitrap mass spectrometer (Thermo Scientific), as previously reported^163^. Raw MS data were processed with MaxQuant software (v2.1.0.0) and searched against the mouse proteome database UniProtKB with 55,366 entries, released in March 2021. Proteomics data were filtered to include only proteins detected in at least three replicate per genotype. Fold-changes and p-values were computed independently for each tissue.

### IMAC phosphoproteomics

Due to the high cost of this commercial survey, only the most strongly affected nervous system area – i.e., spinal cord – was examined. Cerebellar phosphoproteomics was not performed, on the one hand due to financial constraints, and on the other hand because it is more difficult to standardize the ratio between grey and white matter in a comparison of atrophic versus healthy tissue. Mouse cervicothoracic spinal cord tissue was obtained after cervical dislocation from four WT and equal number of end-stage (14-month-old) homozygous KIN mice, then immediately frozen and transported in liquid nitrogen. Phosphoproteome profiling of spinal cord tissue was performed at Cell Signaling Technology INC. using their PTMScan and Fe-IMAC services, as previously described^32^. Searches were conducted against the most recent update of the Uniprot *Mus musculus* database with a mass accuracy of +/-20 ppm for precursor ions and 0.02 Da for productions. Results were filtered with mass accuracy of +/– 5 ppm on precursor ions and the existence of the intended motif.

### Quantitative reverse transcription polymerase chain reaction in spinal cord, cerebellum, and fibroblasts

Total RNA isolation from cerebellar and spinal cord tissue was performed with TRIzol Reagent (Sigma Aldrich, USA) according to manufacturer’s instructions. Total RNA yield and purity were quantified using a Tecan Spark plate reader (Tecan Group Ltd, Switzerland) at 230, 260, and 280 nm, in a NanoQuant plate. cDNA synthesis was performed from 1 µg of total RNA template using the SuperScript IV VILO kit (Invitrogen, USA) according to the manufacturer’s instructions. Gene expression profiles were assessed by quantitative reverse transcription polymerase chain reaction (RT-qPCR) using a StepOnePlus™ (96 well) Real-Time PCR System (Applied Biosystems, USA). RT-qPCRs were run in technical duplicates on cDNA from 25 ng total RNA, with 1 µl TaqMan^®^ Assay, 10 µl FastStart Universal Probe Master 2× (Rox) Mix (Roche, Switzerland) and ddH_2_O up to 20 µl of total volume. The PCR cycling conditions were 50 °C for 2 min, 95 °C for 10 min, followed by 40 cycles of 95 °C for 15 min and 60 °C for 1 min. The gene expression TaqMan^®^ assays (Thermo Fisher Scientific, Waltham, Massachusetts, USA) used for this study were: *Ctsb* (Mm01310506_m1), *Ctsd* (Mm00515586_m1), *Ctsl* (Mm00515597_m1), *Ctss* (Mm01255859_m1), *Ctsz* (Mm00517697_m1), *Klk6* (Mm00478322_m1), *Serpina3n* (Mm00776439_m1), *Serpinb1a* (Mm01610780_m1), *Serpind1* (Mm00433939_m1), and *Serpini1* (Mm00436740_m1). The data were analyzed via the 2^−ΔΔCt^ method^164^, using *Tbp* (Mm00446973_m1) as housekeeping gene as in all our previous mouse publications in view of its optimal robustness and its moderate expression levels.

### Protein extraction and immunoblots

Tissue samples from mouse cerebellum and cervicothoracic spinal cord sections were homogenized with a motor pestle in 5–10× weight/volume amount of RIPA buffer consisting of 50 mM Tris-HCl (pH 8.0), 150 mM NaCl, 2 mM EDTA, 1% Igepal CA-630 (Sigma Aldrich, USA), 0.5% sodium deoxycholate, 0.1% SDS, cOmplete™ Protease Inhibitor Cocktail (Roche, Switzerland), and Halt™ Phosphatase Inhibitor Cocktail (Thermo Fisher Scientific, Inc., USA). The resulting protein suspensions were sonicated, and protein concentration was determined in a Tecan Spark plate reader (Tecan Group Ltd, Switzerland) using a Pierce™ BCA protein assay kit (Thermo Fisher Scientific, Inc., USA). 15 to 25 µg of total proteins were mixed with 2× loading buffer consisting of 250 mM Tris-HCl pH7.4, 20% glycerol, 4% SDS, 10% 2-mercaptoethanol, and 0.005% bromophenol blue, incubated at 90 °C for 5 min, separated on 8–15% polyacrylamide gels at 120 Volts, and transferred to nitrocellulose membranes (0.2 μm) (Bio-Rad Laboratories, Inc., USA). The nitrocellulose membranes were blocked in 5% BSA/TBS-T, and incubated overnight at 4 °C with primary antibodies. Afterwards, the nitrocellulose membranes were incubated for 1 h at room temperature, with fluorescently labeled secondary IRDye^®^ 800CW goat anti-mouse (LI-COR 926-32210, 1:10,000), IRDye^®^ 800CW goat anti-rabbit (LI-COR 926-32211, 1:10,000), IRDye^®^ 680RD goat anti-mouse (LI-COR 926-68070, 1:10,000) or IRDye^®^ 680RD goat anti-rabbit (LI-COR 926-68071, 1:10,000). Membranes were scanned using an Odyssey^®^ Classic Imager. Image visualization and quantification of signal intensities was performed using Image Studio™ software (version 5.2) (LI-COR Biosciences, Ltd., UK). The following primary antibodies were used: KLK6 (Invitrogen PA5−86829, 1:1000), and SERPINB1A (Invitrogen PA5−76875, 1:1000). ACTB (Sigma A5441, 1:10000) or GAPDH (Calbiochem CB1001, 1:10000) served as loading controls.

### Quantification of SERPINB1 plasma levels

The quantification of SERPINB1 plasma levels was done in duplicates using the Human SERPINB1 (Sandwich ELISA) ELISA Kit - LS-F13273 (LS Bio; Seattle, Washington, USA), following manufacturer’s instructions. A four-parameter logistic fitted standard curve for calculating the SERPINB1 plasma levels was generated from the Arigo Biolaboratories website (https://www.arigobio.cn/ELISA-calculator; last accessed on February 17, 2025).

### Clinical and genetic assessments in SCA2 patients

The clinical diagnosis of SCA2 was based on the presence of gait ataxia, dysarthria, dysmetria, dysdiadochokinesis, dysphagia and slow saccades. The age at onset was defined as the onset of motor impairment, and it was ascertained through interviews with patients and close relatives, or the review of patients’ medical records. The Scale for the Assessment and Rating of Ataxia (SARA) score was used as a measure of ataxia severity. This scale ranges from zero to 40 points, increasing with cerebellar disease progression^165^. In addition, the Inventory Non-Ataxia Symptoms (INAS) was applied as a measure of disease severity in terms of extracerebellar involvement^166^. Additional information regarding age, sex, and disease duration in years from disease onset to latest examination, was retrieved. Disease stage was assessed as previously specified^82^.

### Bioinformatics analyses

STRING web-server (https://string-db.org/) (version 11.0, last accessed on March 4, 2025)^167^ was used for pathway enrichment analyses using (i) lists of dysregulated transcripts identified in the KIN cerebellar and spinal cord transcriptome profiling, or (ii) lists of commonly dysregulated factors in the KIN cerebellar and spinal cord transcriptomes and proteomes, and in the spinal cord phosphoproteome, as input. Different functional classification frameworks were used, including Gene Ontology (GO), Kyoto Encyclopedia of Genes and Genomes (KEGG) Pathways, Reactome Pathways, and The Mammalian Phenotype Ontology (Monarch). Venn diagrams created using the Venn diagram web tool from the University of Ghent, Ghent, Belgium (http://bioinformatics.psb.ugent.be/webtools/Venn; last accessed on February 28, 2025), were used to identify factors commonly dysregulated across omic datasets. The PhosphoSitePlus^®^ database was searched for dysregulated phospho-sites identified in the KIN mice spinal cord phospho-proteome profiling^168^ (www.phosphositeplus.org; last accessed on March 10, 2025).

### Statistical analyses

Unpaired Student t-tests with Welch’s correction were used to establish comparisons for continuous variables between homozygous *Atxn2*-CAG100-KIN and WT animals, and between SCA2 patients and control individuals. Additional mean comparisons for SERPINB1 plasma levels between SCA2 patients and control individuals were conducted using paired t-test, Mann-Whitney U, and Wilcoxon W non-parametric tests in the whole datasets and after data trimming. Symmetric data trimming was performed by removing 10% or 20% of the highest and lowest values in both comparison groups, whereas asymmetric data trimming involved removing a subcluster of control individuals with SERPINB1 plasma levels exceeding 5.5 ng/mL. Analysis of covariance (ANCOVA) was conducted to assess the impact of age, sex, group (SCA2 patients *versus* controls), CAG repeat, the interaction between group and CAG repeat, and an indicator variable (CtrlHighSERPINB1) accounting for control-group heterogeneity, on SERPINB1 plasma levels. A receiver operating characteristic (ROC) analysis was conducted to assess the discriminative power of SERPINB1 as a candidate diagnostic biomarker. The term “nominal significance” refers to the threshold of *p* < 0.05, multiple testing corrections were not applied to our multi-omics analyses by Venn diagrams. Multiple testing corrections by false discovery rates (FDR) were at the base of pathway enrichment data by STRING. Chi-square test was applied to look for differences in sex distributions between SCA2 patients and control individuals. Normal distribution was verified for all variables using the Kolmogorov–Smirnov test. Correlations between blood plasma SERPINB1 levels and markers of disease severity in the SCA2 cohort were established by using the Pearson correlation test. The age at onset was corrected for the CAG repeat length at expanded *ATXN2* alleles by simple linear regression. Scatter plots, Q-Q plots and residual diagnostics were run for linear regression analyses. Bar charts depicting the mean and standard error of the mean (SEM) values, and a correlation matrix diagram were used for data visualization. All statistical analyses were conducted using GraphPad Prism software (version 8.4.2) (GraphPad Software Inc., USA). Significance was assumed at *p* < 0.05 and highlighted with asterisks: Trend (T) < 0.01; *p < 0.05, **p < 0.01, ***p < 0.001, ****p < 0.0001.

## Supplementary Information

Below is the link to the electronic supplementary material.

Supplementary Material 1

Supplementary Material 2

Supplementary Material 3

Supplementary Material 4

Supplementary Material 5

Supplementary Material 6

Supplementary Material 7

Supplementary Material 8

Supplementary Material 9

Supplementary Material 10

Supplementary Material 11

Supplementary Material 12

Supplementary Material 13

Supplementary Material 14

Supplementary Material 15

Supplementary Material 16

Supplementary Material 17

Supplementary Material 18

Supplementary Material 19

Supplementary Material 20

Supplementary Material 21

Supplementary Material 22

Supplementary Material 23

Supplementary Material 24

Supplementary Material 25

Supplementary Material 26

## Data Availability

Detailed data on the examined transcriptionally dysregulated factors may be found in supplemental Tables S1-5, available with the online version of this article. Additional datasets used during the current study are available from the corresponding author on reasonable request.
