# Supplementary material for: Multiomics approach identifies SERPINB1 as candidate biomarker for spinocerebellar ataxia type 2
Source: Sci Rep. 2025 Nov 26;15:42559. doi: 10.1038/s41598-025-29070-7 (PMC12663351; doi:10.1038/s41598-025-29070-7)
Supplement: Supplementary file 1 — Supplementary Material 1 [file 41598_2025_29070_MOESM1_ESM.docx]

| **Pathology features shared between disease and model** | **SCA2 patients** | ***Atxn2*-CAG100-KnockIn mouse** |
| --- | --- | --- |
| Corticospinal tract degeneration | 12039668,  **27417041,**  **27730516,**  **29101844,**  **29550649** | **31376479** |
| Spinal motor neuron loss, peripheral neuropathy | **14967775,**  31228263, 38383154 | **31376479** |
| Cytoplasmic polyQ aggregates in  brainstem / spinal cord | **11872620,**  **21134000,**  **27377427** | 22956915,  **31376479,**  **33577922** |
| Cytoplasmic TDP-43 aggregates in  spinal motor neurons | **24718895, 35511239** | **33577922** |
| Deficits of cholesterol, ceramides, sphingolipids | **31766565** | **31766565** |
| Cerebellar Purkinje cell affection / calcium anomaly / spine loss / glutamatergic input deficit | **18418684,**  **23438480,**  30590599 | 32932600 |
| Cerebellar deficit of N-acetylaspartate and glutamate | **20838948,**  **25773989,**  **29575033** | **31376479** |
| Prominent demyelination | **15896478,**  **33029780,**  **33625581** | doi: 10.1101/ 2025.08.08.669189 |
| Initial polyphagia / obesity / loss-of-function, then dysphagia / weight deficit / toxic gain-of-function | **18297329, 33687306** | **31376479** |
