# Supplementary material for: Multiomics approach identifies SERPINB1 as candidate biomarker for spinocerebellar ataxia type 2
Source: Sci Rep. 2025 Nov 26;15:42559. doi: 10.1038/s41598-025-29070-7 (PMC12663351; doi:10.1038/s41598-025-29070-7)
Supplement: Supplementary file 6 — Supplementary Material 6 [file 41598_2025_29070_MOESM6_ESM.docx]

**Table S8:** Clinical and molecular characteristics of the sex- and age-matched SCA2 patients and control individuals.

| Variables | SCA2 patients (N=58) | Controls (N=58) | χ^2^/t-test (p-value) |
| --- | --- | --- | --- |
|  | Mean (SD) | Mean (SD) |  |
| Sex (M/F) | 25/33 | 19/39 | 1.318 (0.251) |
| Age (years) | 51.12 (13.09) | 49.48 (12.53) | -0.688 (0.493) |
| SERPINB1 (ng/ml) | 3.07 (1.342) | 4.05 (2.459) | 2.618 (0.010) |
| Age at Onset (yrs) | 38.97 (11.31) | - | - |
| Disease Duration (yrs) | 12.89 (5.880) | - | - |
| SARA score | 16.08 (8.214) | - | - |
| INAS count | 3.34 (1.428) | - | - |
| *ATXN2* expanded alleles (CAG repeat length) | 38.6 (3.031) | - | - |
