# Supplementary figures and images for: Multiomics approach identifies SERPINB1 as candidate biomarker for spinocerebellar ataxia type 2
Source: Sci Rep. 2025 Nov 26;15:42559. doi: 10.1038/s41598-025-29070-7 (PMC12663351; doi:10.1038/s41598-025-29070-7)

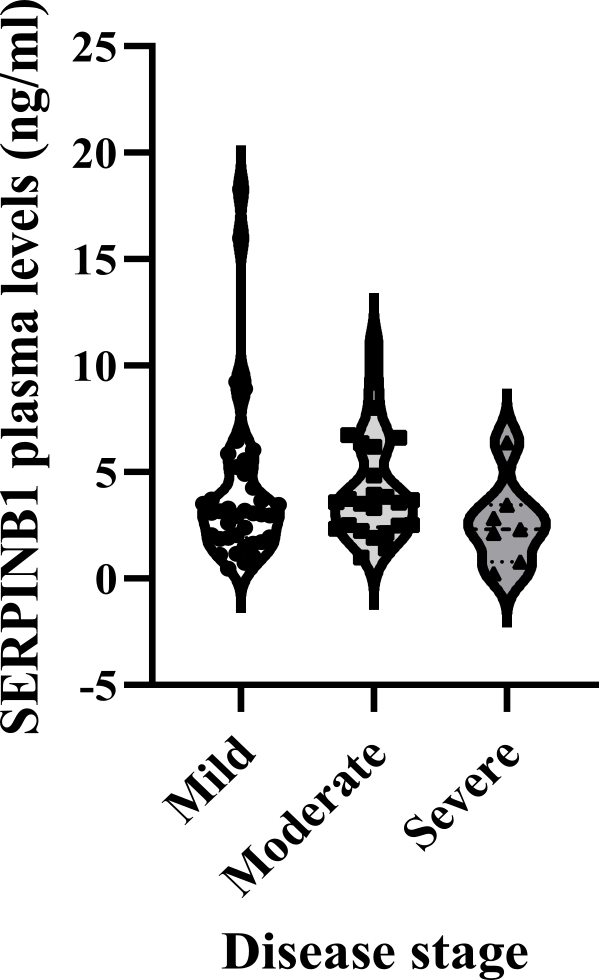

Supplement: Supplementary file 9 — Supplementary Material 9 [file 41598_2025_29070_MOESM9_ESM.tiff]

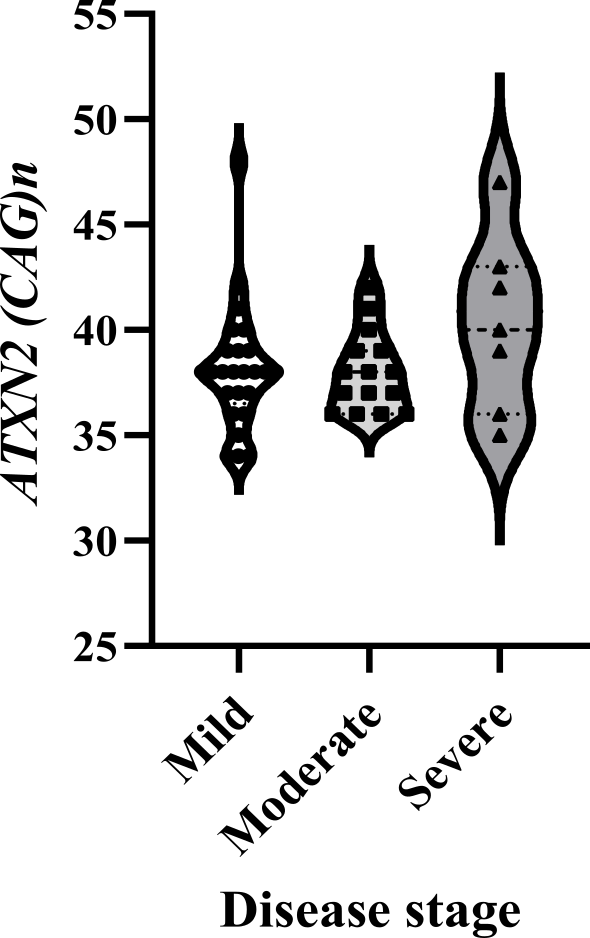

Supplement: Supplementary file 10 — Supplementary Material 10 [file 41598_2025_29070_MOESM10_ESM.tiff]

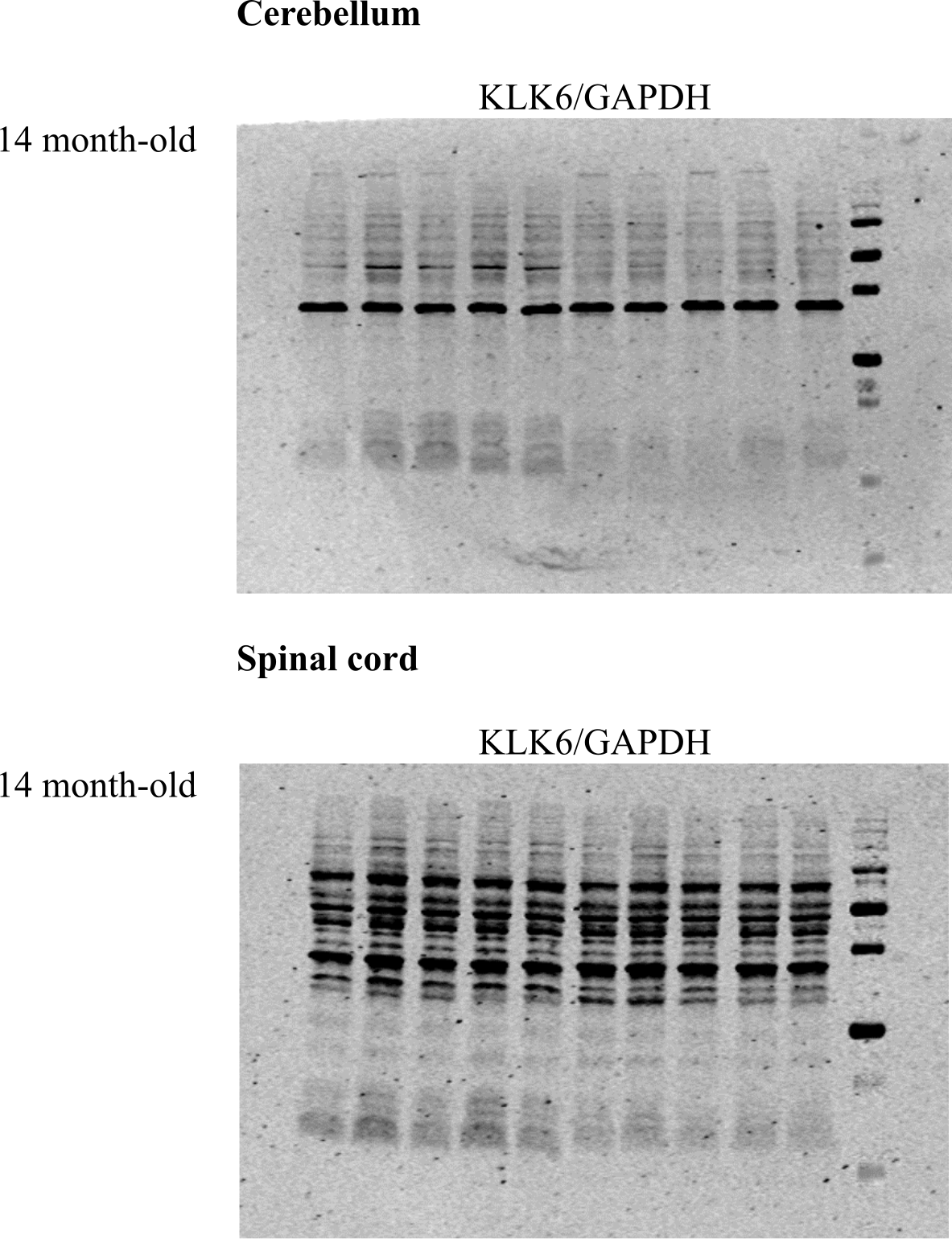

Supplement: Supplementary file 11 — Supplementary Material 11 [file 41598_2025_29070_MOESM11_ESM.tiff]

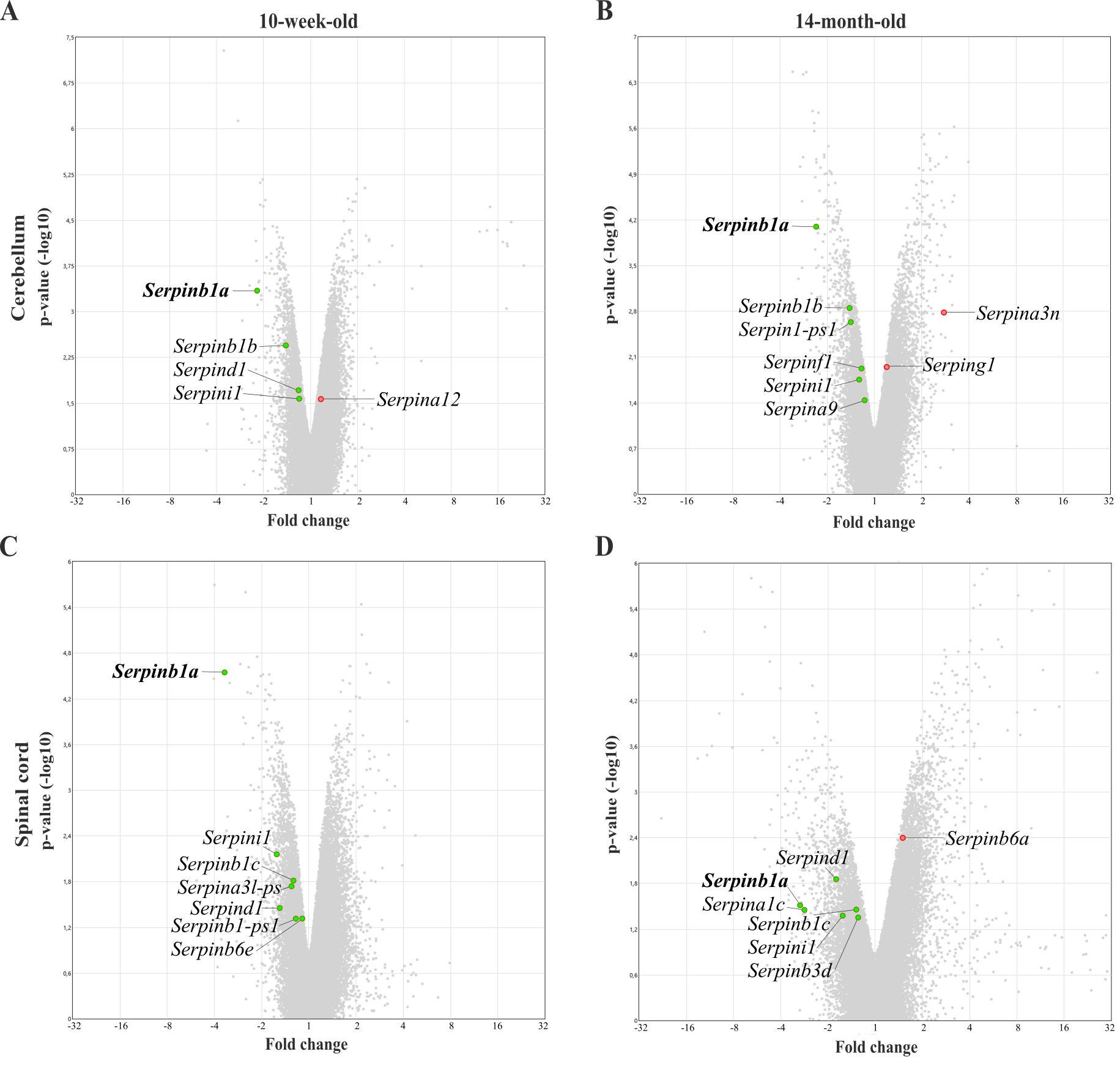

Supplement: Supplementary file 12 — Supplementary Material 12 [file 41598_2025_29070_MOESM12_ESM.tiff]

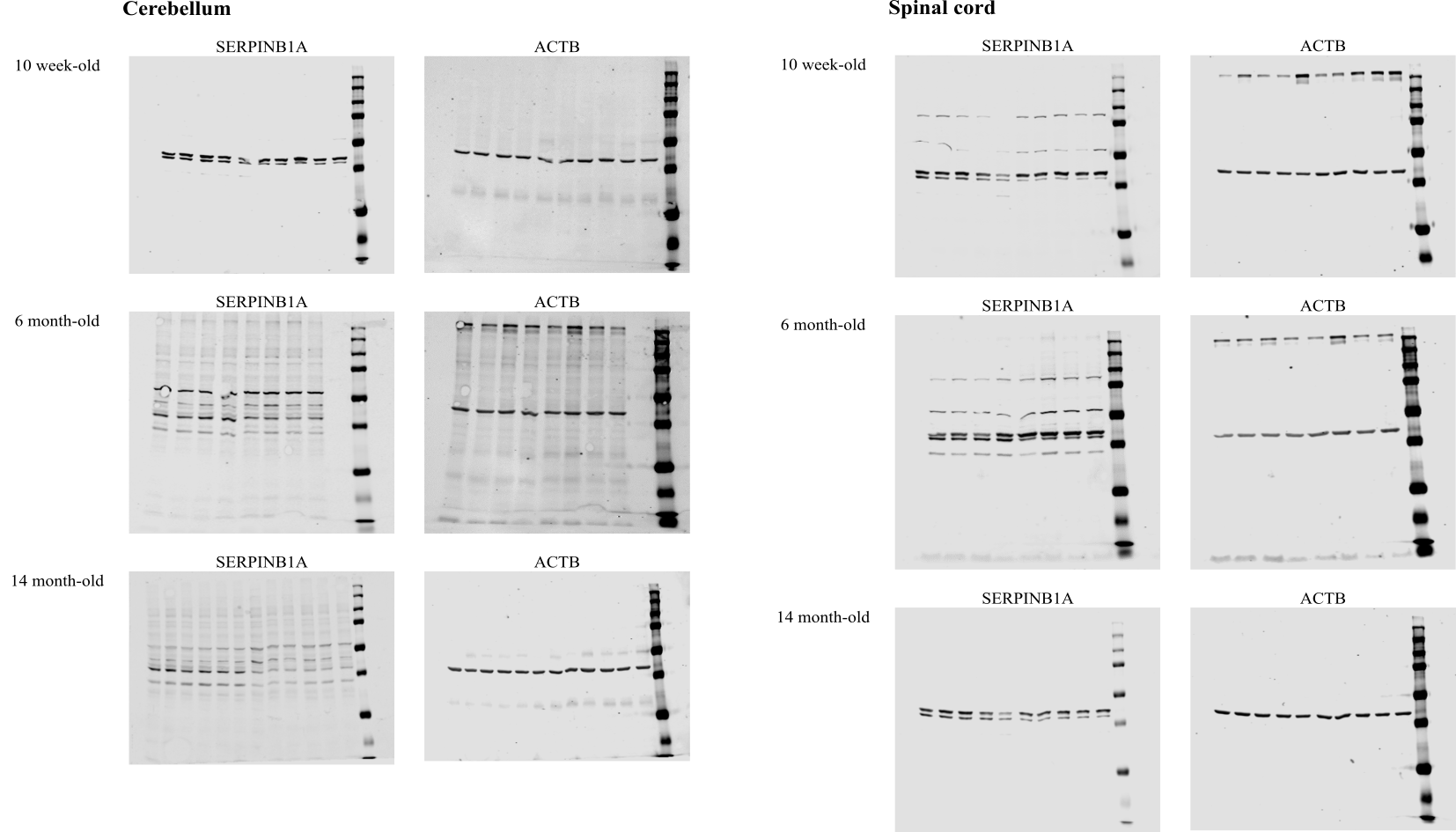

Supplement: Supplementary file 13 — Supplementary Material 13 [file 41598_2025_29070_MOESM13_ESM.tiff]

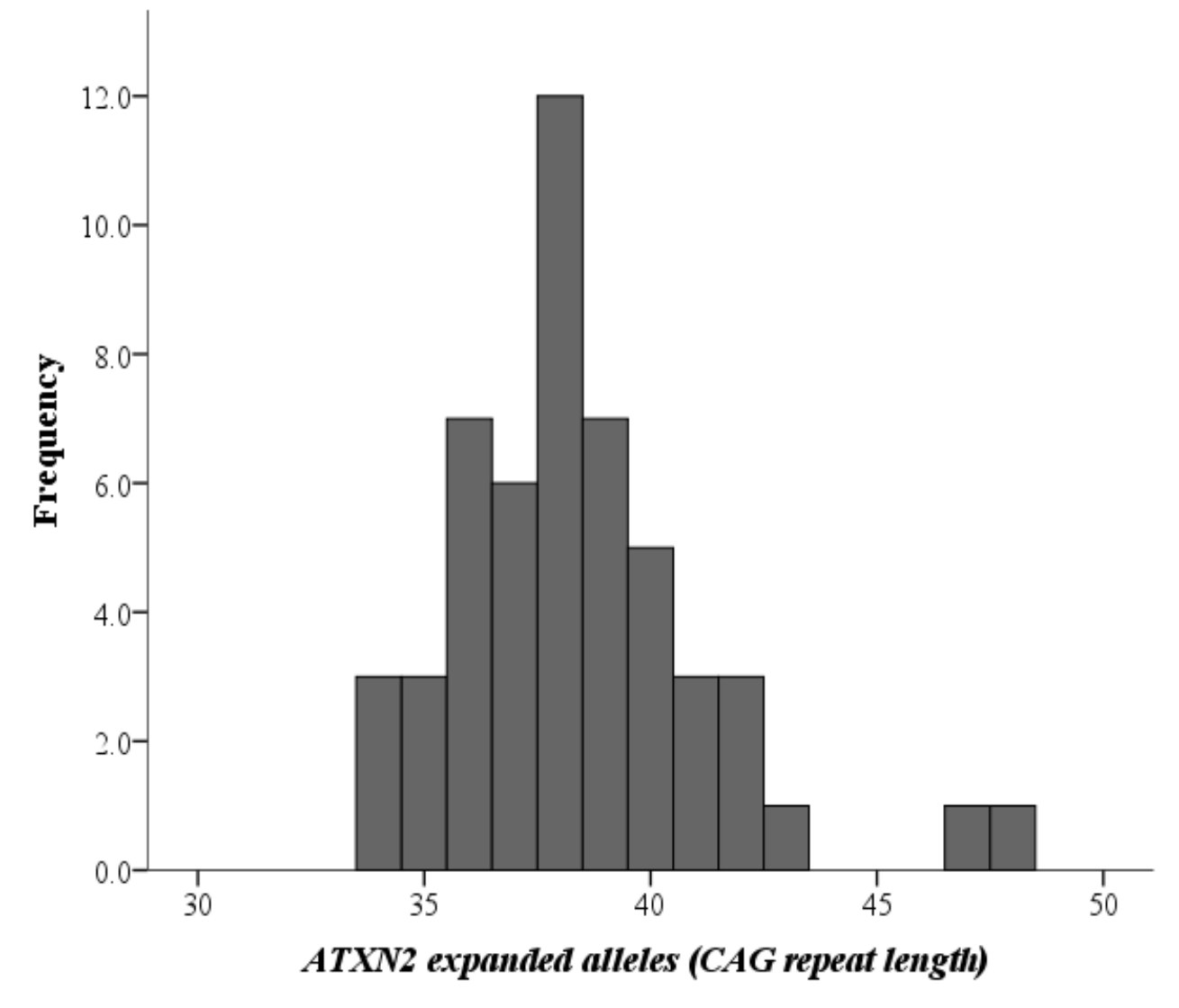

Supplement: Supplementary file 14 — Supplementary Material 14 [file 41598_2025_29070_MOESM14_ESM.tiff]

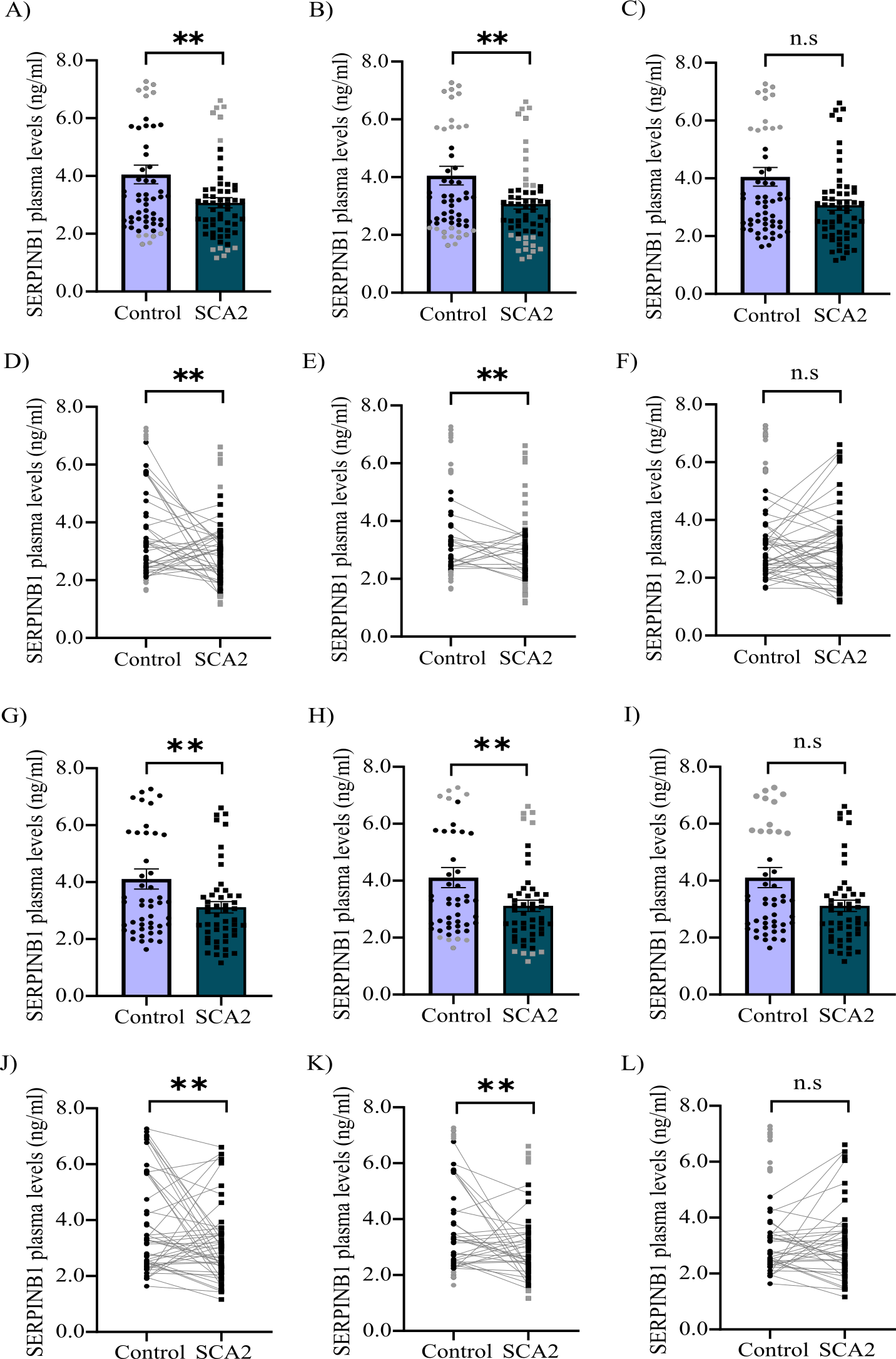

Supplement: Supplementary file 15 — Supplementary Material 15 [file 41598_2025_29070_MOESM15_ESM.tiff]

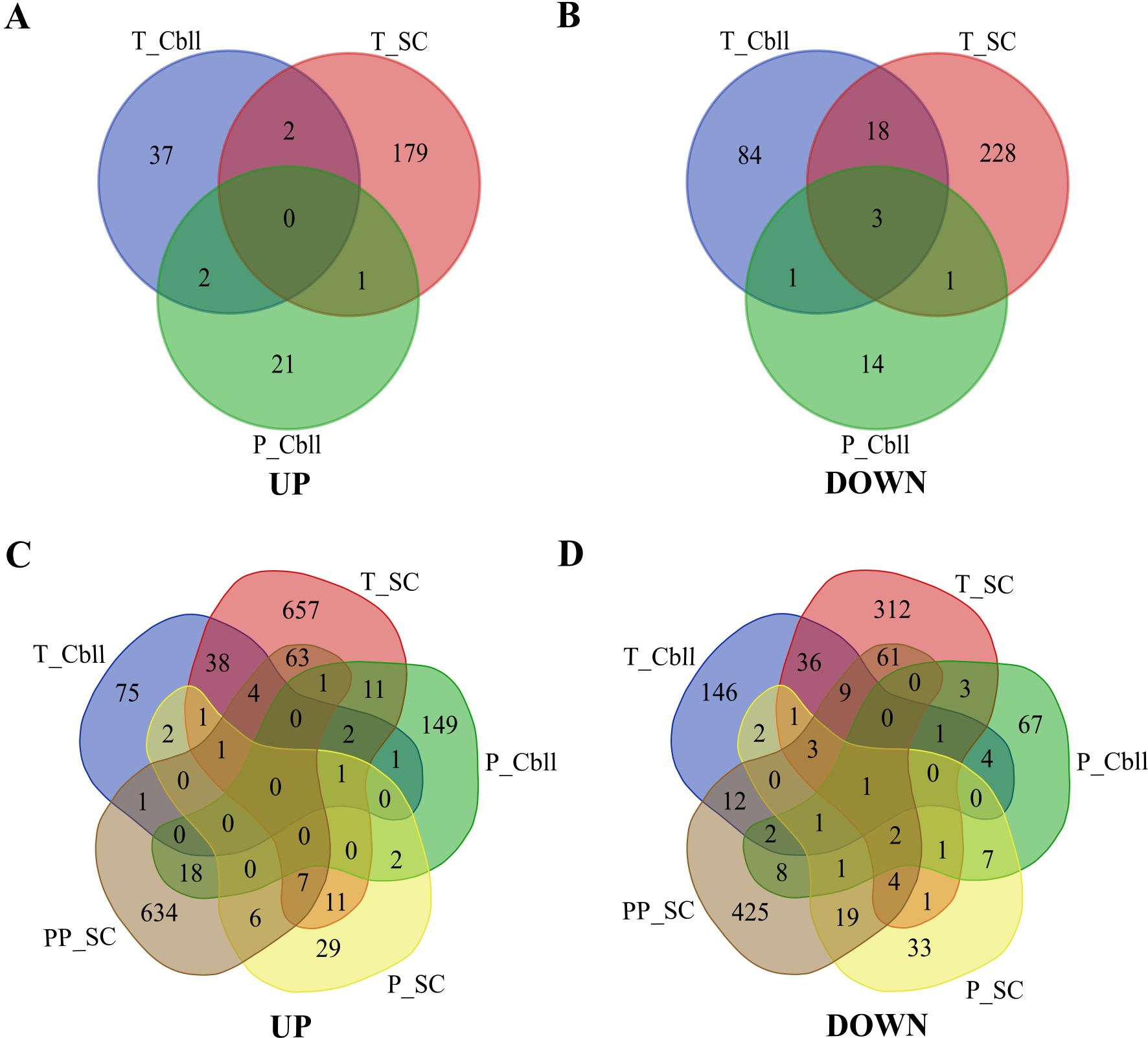

Supplement: Supplementary file 16 — Supplementary Material 16 [file 41598_2025_29070_MOESM16_ESM.tiff]

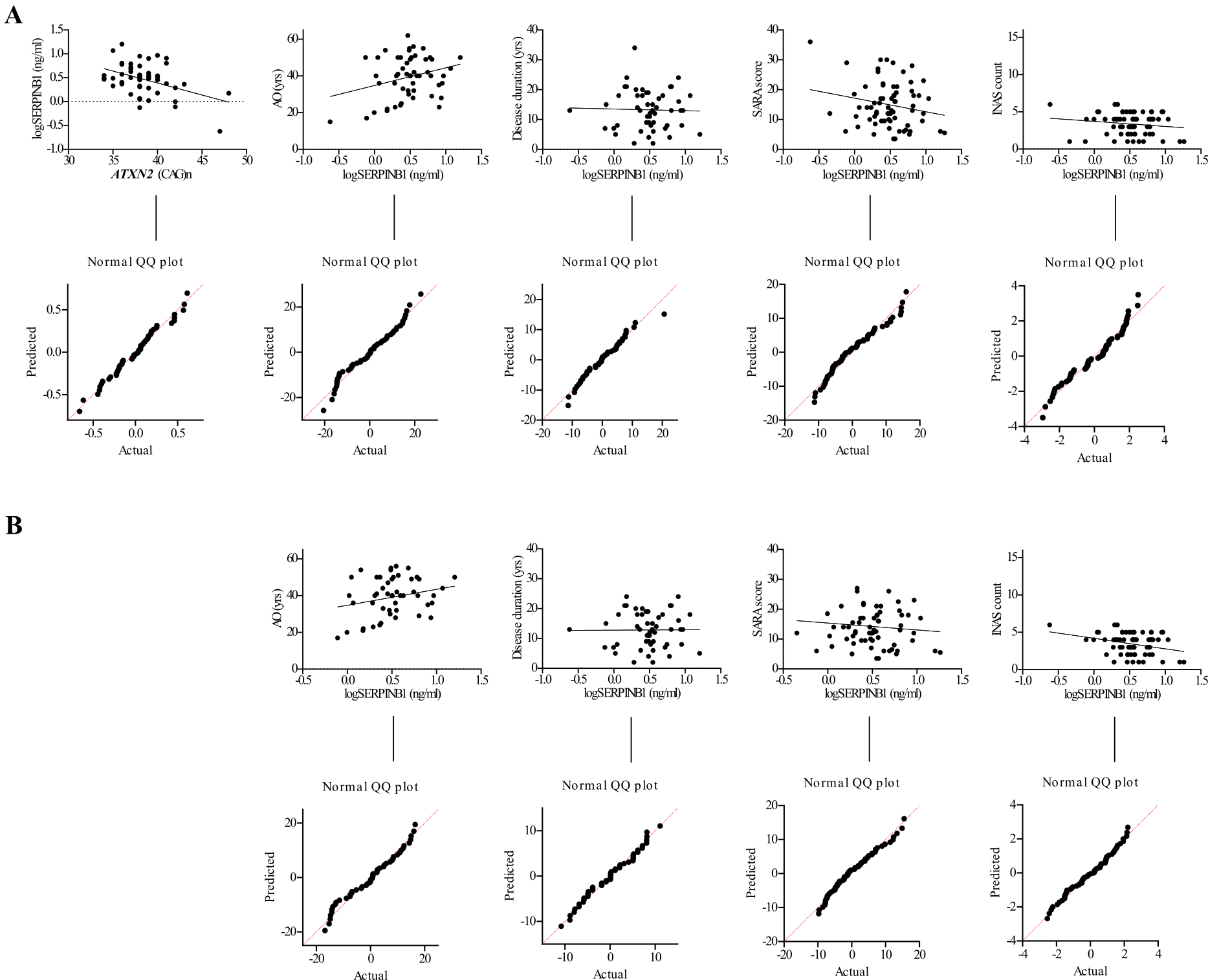

Supplement: Supplementary file 17 — Supplementary Material 17 [file 41598_2025_29070_MOESM17_ESM.tiff]

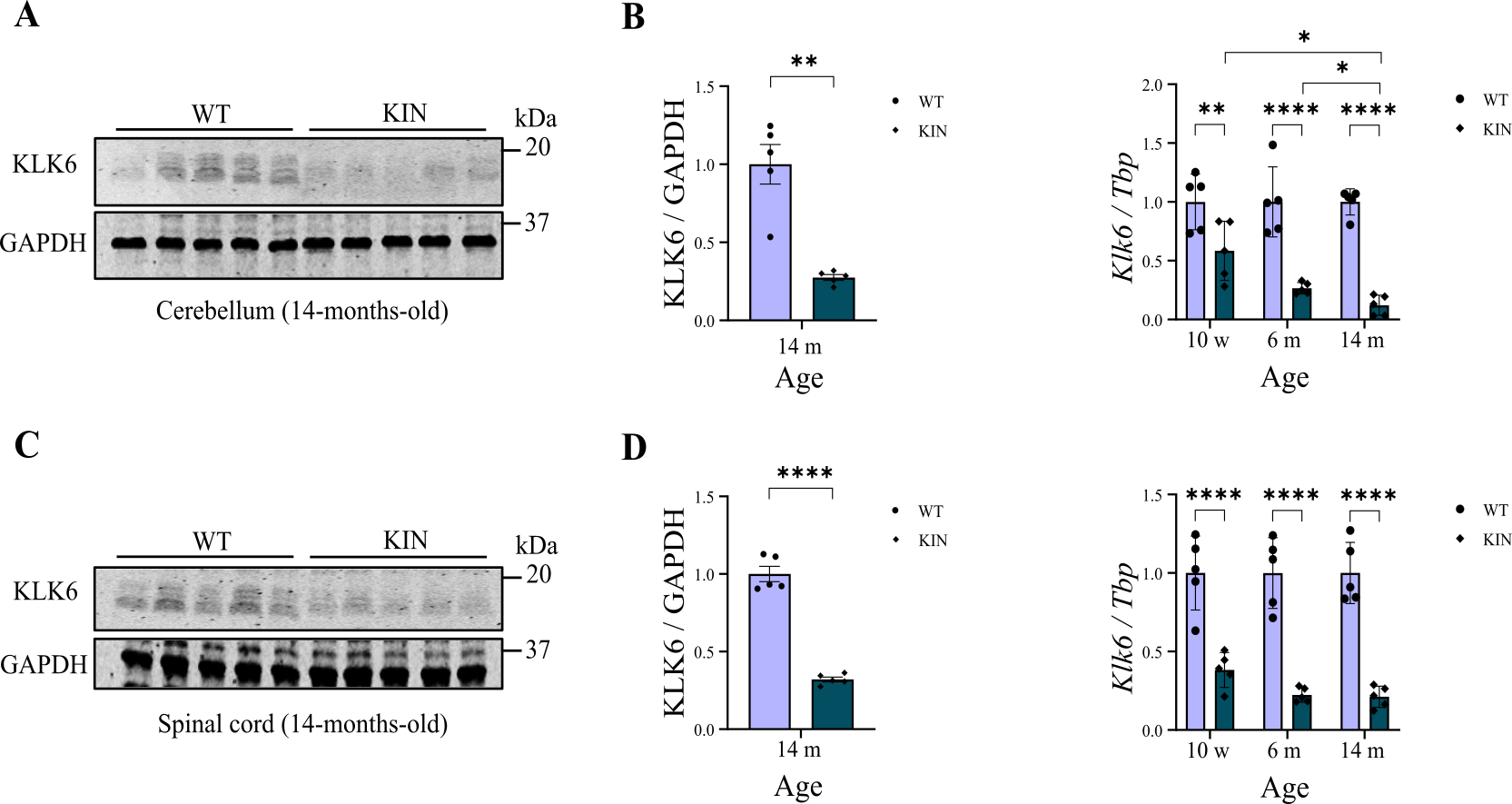

Supplement: Supplementary file 18 — Supplementary Material 18 [file 41598_2025_29070_MOESM18_ESM.tiff]

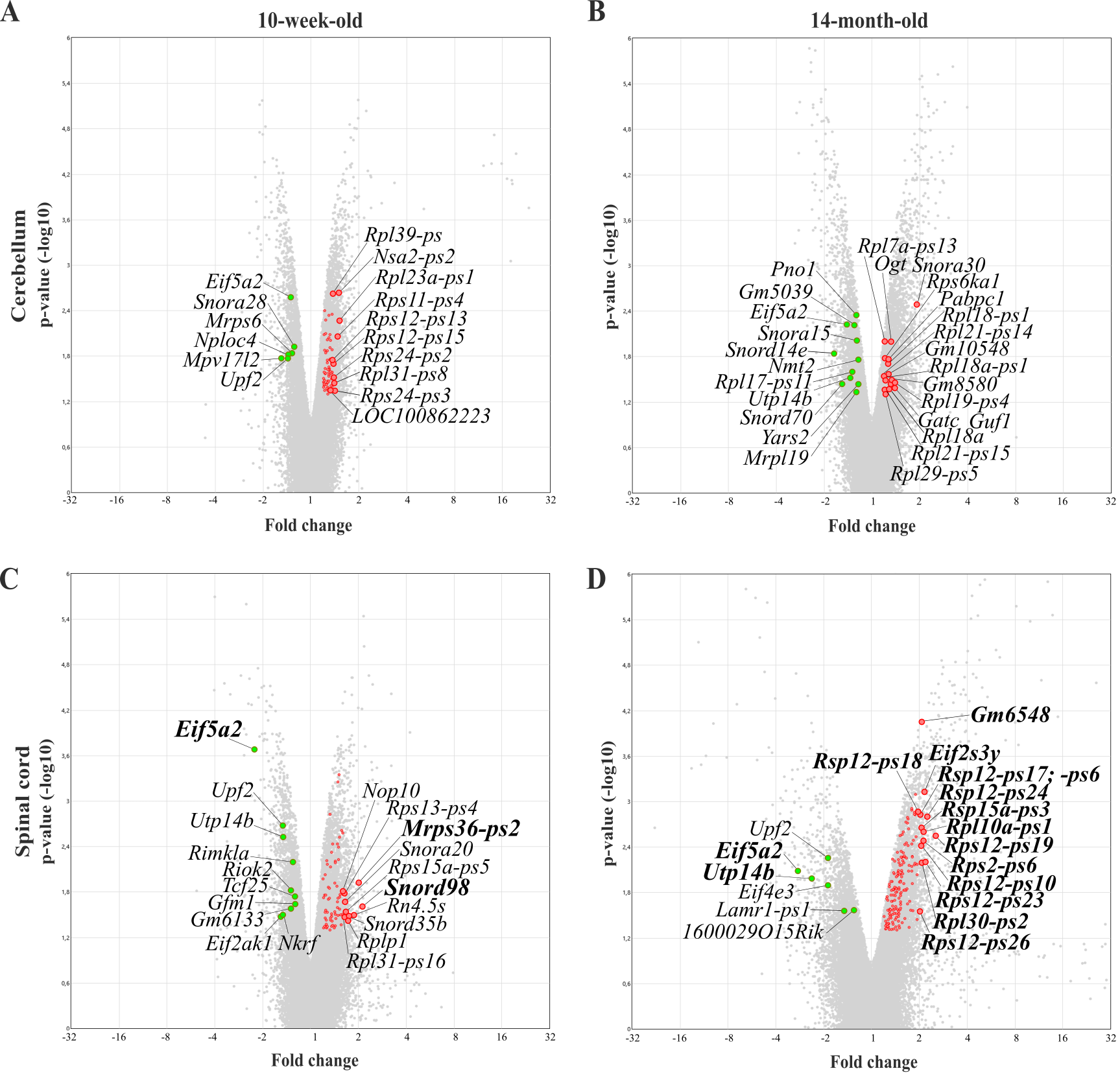

Supplement: Supplementary file 19 — Supplementary Material 19 [file 41598_2025_29070_MOESM19_ESM.tiff]

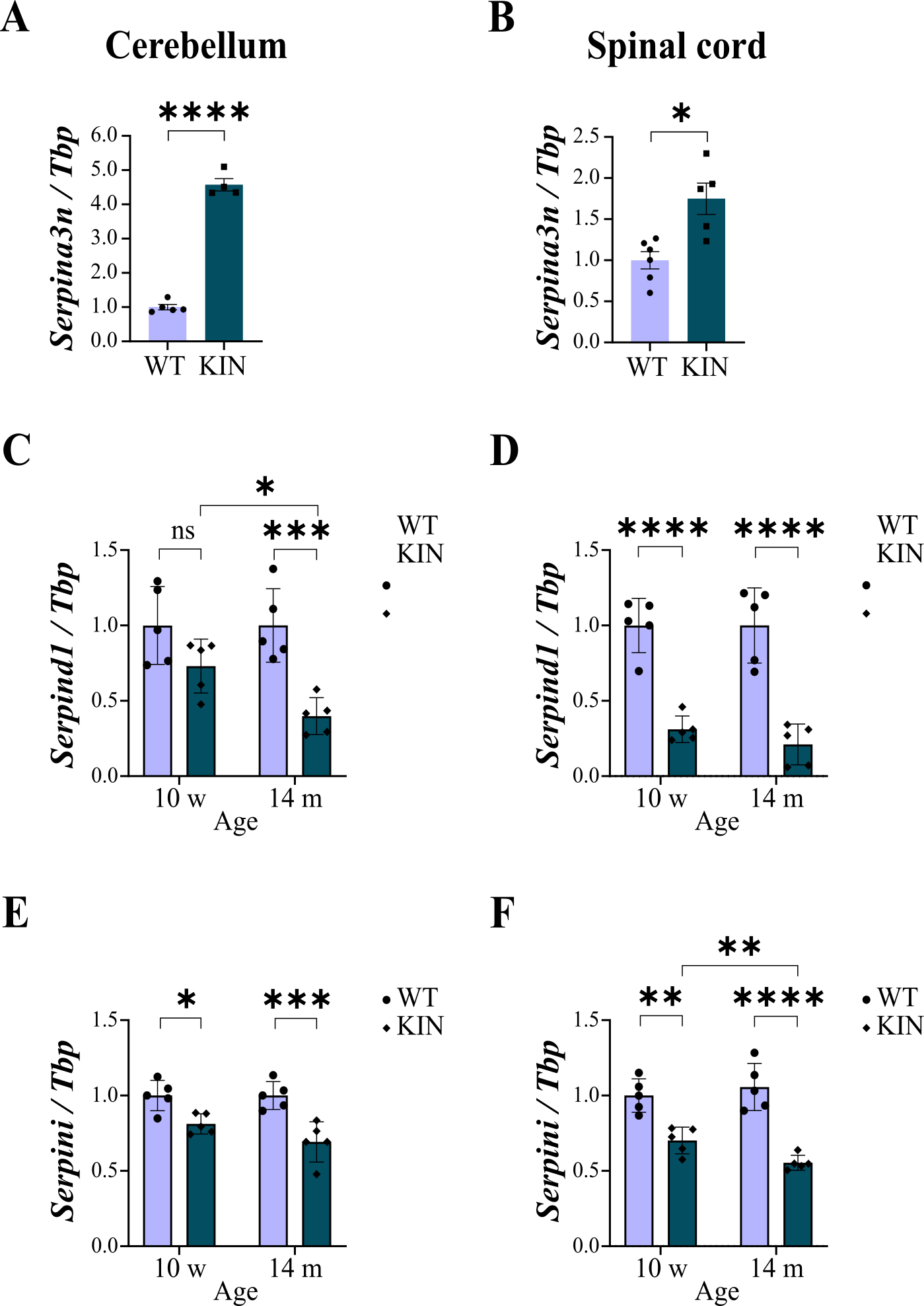

Supplement: Supplementary file 20 — Supplementary Material 20 [file 41598_2025_29070_MOESM20_ESM.tiff]

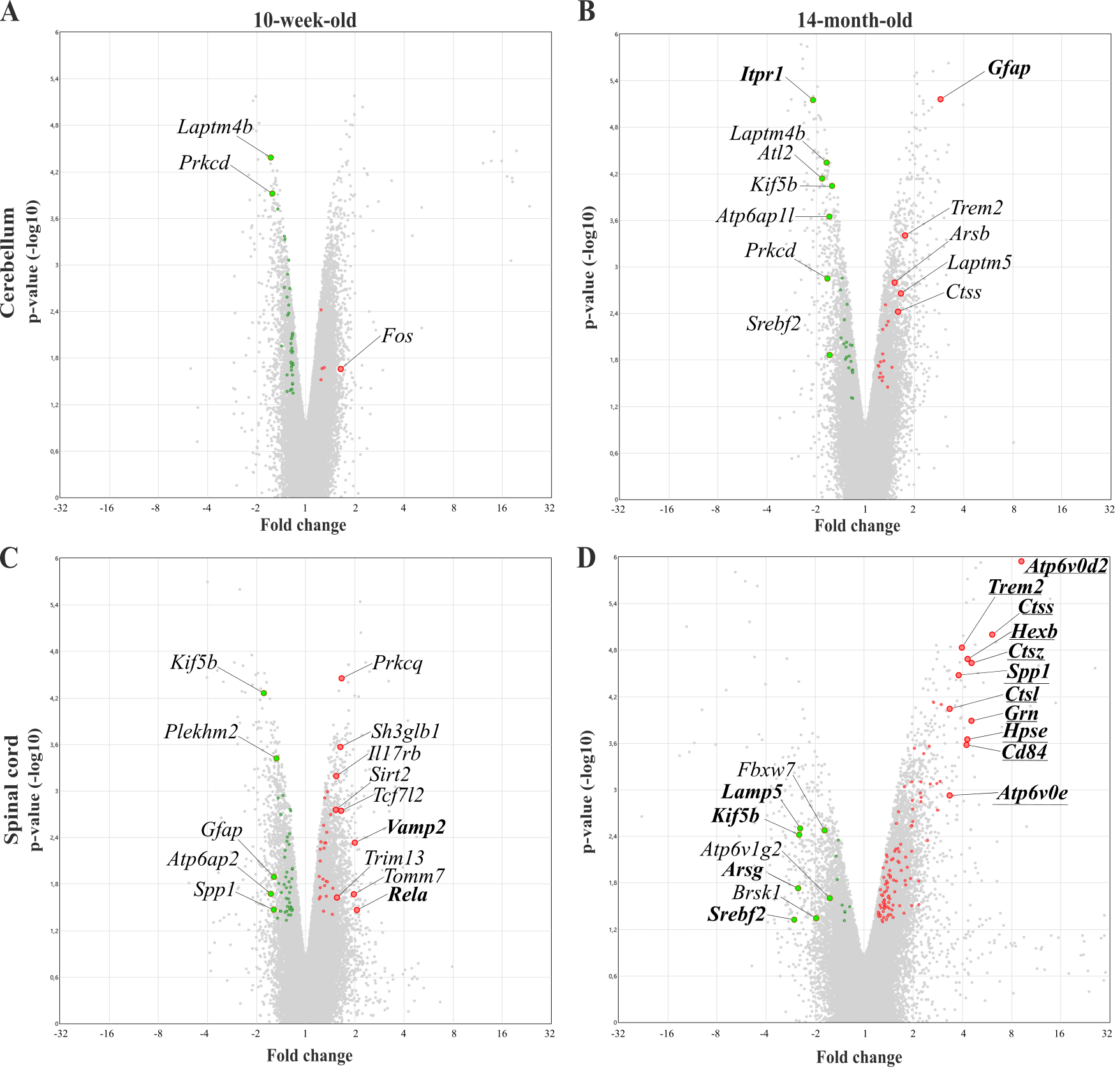

Supplement: Supplementary file 21 — Supplementary Material 21 [file 41598_2025_29070_MOESM21_ESM.tiff]

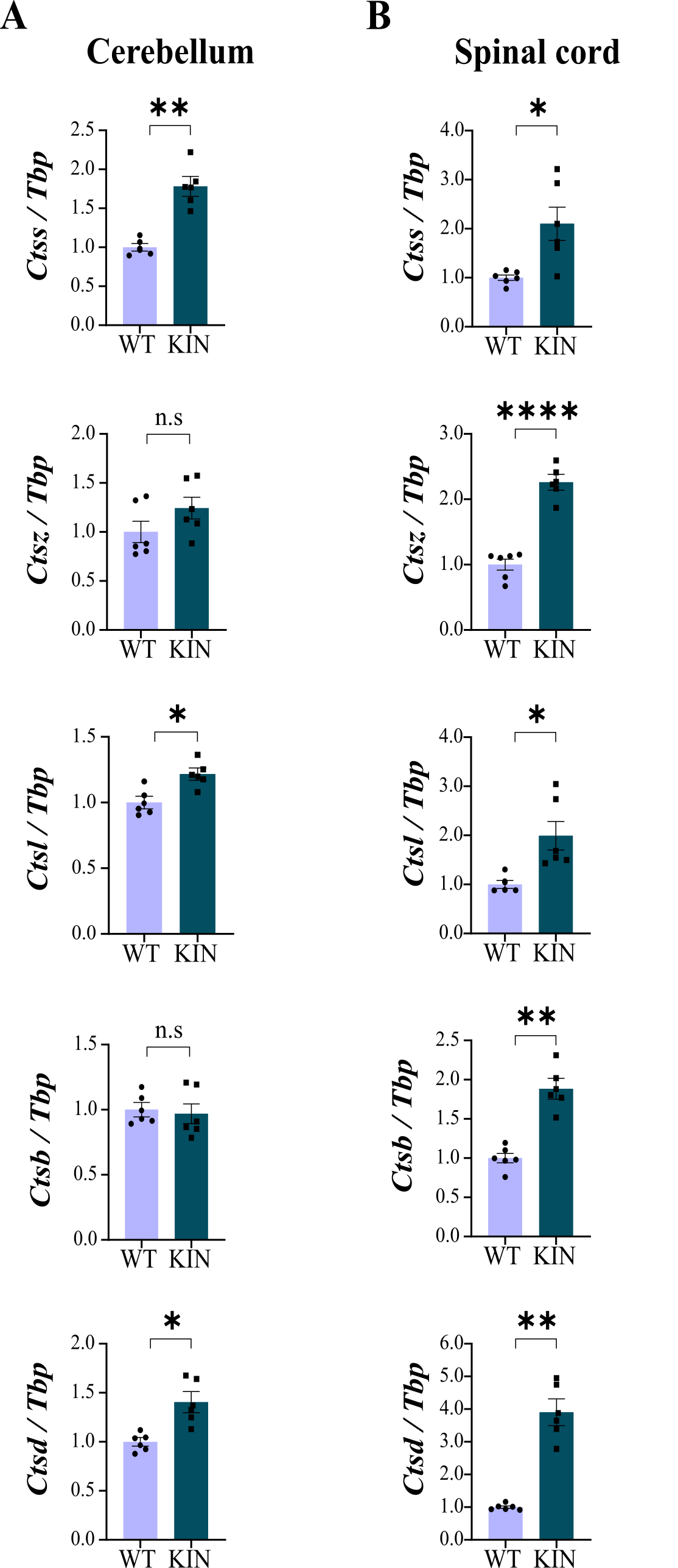

Supplement: Supplementary file 22 — Supplementary Material 22 [file 41598_2025_29070_MOESM22_ESM.tiff]

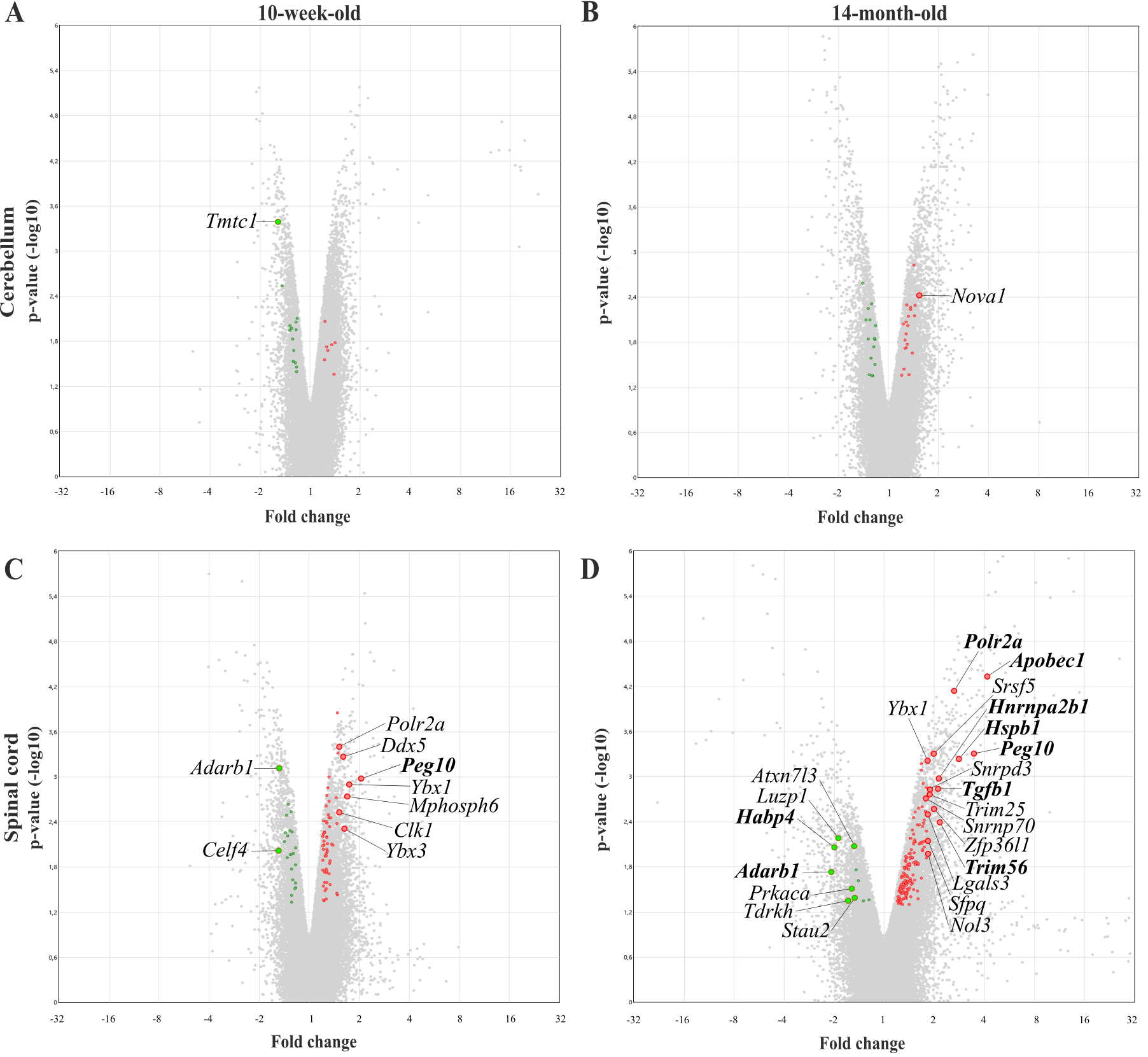

Supplement: Supplementary file 23 — Supplementary Material 23 [file 41598_2025_29070_MOESM23_ESM.tiff]
