# Supplementary material for: Multiomics approach identifies SERPINB1 as candidate biomarker for spinocerebellar ataxia type 2
Source: Sci Rep. 2025 Nov 26;15:42559. doi: 10.1038/s41598-025-29070-7 (PMC12663351; doi:10.1038/s41598-025-29070-7)
Supplement: Supplementary file 24 — Supplementary Material 24 [file 41598_2025_29070_MOESM24_ESM.docx]

**Receiver‑operator characteristic curve analysis for SERPINB1 plasma levels in patients with SCA2 versus control individuals**

| **Area under the ROC curve** | **58 vs 58** | **49 vs 49** |
| --- | --- | --- |
| Area | 0.6098 | 0.6183 |
| Std. Error | 0.05215 | 0.05639 |
| 95% confidence interval | 0.4837 to 0.6934 | 0.4892 to 0.7149 |
| P value | 0.0413 | 0.0436 |


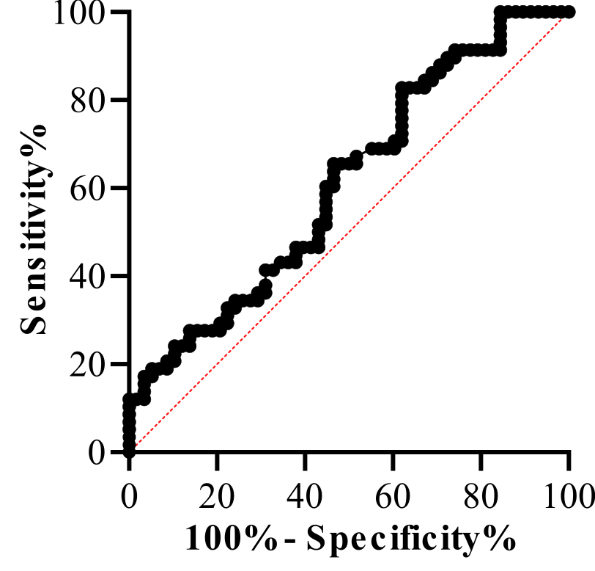


**Sensitivity and specificity analysis from the dataset of 58 vs 58 individuals**

| **Cut-off values** | **Sensitivity (%)** | **95% CI** | **Specificity (%)** | **95% CI** |
| --- | --- | --- | --- | --- |
| < 1.201 | 1.724 | 0.08844% to 9.141% | 100.0 | 93.79% to 100.0% |
| < 1.333 | 3.448 | 0.6127% to 11.73% | 100.0 | 93.79% to 100.0% |
| < 1.439 | 5.172 | 1.410% to 14.14% | 100.0 | 93.79% to 100.0% |
| < 1.473 | 6.897 | 2.714% to 16.43% | 100.0 | 93.79% to 100.0% |
| < 1.502 | 8.621 | 3.739% to 18.64% | 100.0 | 93.79% to 100.0% |
| < 1.571 | 10.34 | 4.828% to 20.79% | 100.0 | 93.79% to 100.0% |
| < 1.635 | 12.07 | 5.971% to 22.88% | 100.0 | 93.79% to 100.0% |
| < 1.661 | 12.07 | 5.971% to 22.88% | 98.28 | 90.86% to 99.91% |
| < 1.698 | 12.07 | 5.971% to 22.88% | 96.55 | 88.27% to 99.39% |
| < 1.791 | 13.79 | 7.158% to 24.93% | 96.55 | 88.27% to 99.39% |
| < 1.887 | 15.52 | 8.384% to 26.93% | 96.55 | 88.27% to 99.39% |
| < 1.908 | 17.24 | 9.644% to 28.91% | 96.55 | 88.27% to 99.39% |
| < 1.916 | 17.24 | 9.644% to 28.91% | 94.83 | 85.86% to 98.59% |
| < 1.921 | 18.97 | 10.93% to 30.85% | 94.83 | 85.86% to 98.59% |
| < 1.937 | 18.97 | 10.93% to 30.85% | 93.10 | 83.57% to 97.29% |
| < 1.970 | 18.97 | 10.93% to 30.85% | 91.38 | 81.36% to 96.26% |
| < 1.997 | 20.69 | 12.25% to 32.77% | 91.38 | 81.36% to 96.26% |
| < 2.018 | 20.69 | 12.25% to 32.77% | 89.66 | 79.21% to 95.17% |
| < 2.035 | 22.41 | 13.59% to 34.66% | 89.66 | 79.21% to 95.17% |
| < 2.066 | 24.14 | 14.96% to 36.53% | 89.66 | 79.21% to 95.17% |
| < 2.098 | 24.14 | 14.96% to 36.53% | 87.93 | 77.12% to 94.03% |
| < 2.108 | 24.14 | 14.96% to 36.53% | 86.21 | 75.07% to 92.84% |
| < 2.125 | 25.86 | 16.35% to 38.38% | 86.21 | 75.07% to 92.84% |
| < 2.142 | 27.59 | 17.75% to 40.20% | 86.21 | 75.07% to 92.84% |
| < 2.179 | 27.59 | 17.75% to 40.20% | 84.48 | 73.07% to 91.62% |
| < 2.221 | 27.59 | 17.75% to 40.20% | 82.76 | 71.09% to 90.36% |
| < 2.239 | 27.59 | 17.75% to 40.20% | 81.03 | 69.15% to 89.07% |
| < 2.246 | 27.59 | 17.75% to 40.20% | 79.31 | 67.23% to 87.75% |
| < 2.278 | 29.31 | 19.18% to 42.01% | 79.31 | 67.23% to 87.75% |
| < 2.309 | 29.31 | 19.18% to 42.01% | 77.59 | 65.34% to 86.41% |
| < 2.320 | 31.03 | 20.62% to 43.80% | 77.59 | 65.34% to 86.41% |
| < 2.340 | 32.76 | 22.08% to 45.58% | 77.59 | 65.34% to 86.41% |
| < 2.367 | 32.76 | 22.08% to 45.58% | 75.86 | 63.47% to 85.04% |
| < 2.393 | 34.48 | 23.56% to 47.33% | 75.86 | 63.47% to 85.04% |
| < 2.412 | 34.48 | 23.56% to 47.33% | 74.14 | 61.62% to 83.65% |
| < 2.439 | 34.48 | 23.56% to 47.33% | 72.41 | 59.80% to 82.25% |
| < 2.470 | 34.48 | 23.56% to 47.33% | 70.69 | 57.99% to 80.82% |
| < 2.485 | 36.21 | 25.05% to 49.07% | 70.69 | 57.99% to 80.82% |
| < 2.490 | 36.21 | 25.05% to 49.07% | 68.97 | 56.20% to 79.38% |
| < 2.501 | 37.93 | 26.56% to 50.80% | 68.97 | 56.20% to 79.38% |
| < 2.511 | 41.38 | 29.63% to 54.20% | 68.97 | 56.20% to 79.38% |
| < 2.514 | 41.38 | 29.63% to 54.20% | 67.24 | 54.42% to 77.92% |
| < 2.538 | 43.10 | 31.18% to 55.88% | 65.52 | 52.67% to 76.44% |
| < 2.576 | 43.10 | 31.18% to 55.88% | 63.79 | 50.93% to 74.95% |
| < 2.607 | 43.10 | 31.18% to 55.88% | 62.07 | 49.20% to 73.44% |
| < 2.651 | 44.83 | 32.75% to 57.55% | 62.07 | 49.20% to 73.44% |
| < 2.688 | 46.55 | 34.33% to 59.20% | 62.07 | 49.20% to 73.44% |
| < 2.717 | 46.55 | 34.33% to 59.20% | 60.34 | 47.49% to 71.91% |
| < 2.770 | 46.55 | 34.33% to 59.20% | 58.62 | 45.80% to 70.37% |
| < 2.817 | 46.55 | 34.33% to 59.20% | 56.90 | 44.12% to 68.82% |
| < 2.841 | 48.28 | 35.93% to 60.84% | 56.90 | 44.12% to 68.82% |
| < 2.906 | 50.00 | 37.54% to 62.46% | 56.90 | 44.12% to 68.82% |
| < 2.988 | 51.72 | 39.16% to 64.07% | 56.90 | 44.12% to 68.82% |
| < 3.016 | 51.72 | 39.16% to 64.07% | 55.17 | 42.45% to 67.25% |
| < 3.043 | 53.45 | 40.80% to 65.67% | 55.17 | 42.45% to 67.25% |
| < 3.075 | 55.17 | 42.45% to 67.25% | 55.17 | 42.45% to 67.25% |
| < 3.107 | 56.90 | 44.12% to 68.82% | 55.17 | 42.45% to 67.25% |
| < 3.133 | 58.62 | 45.80% to 70.37% | 55.17 | 42.45% to 67.25% |
| < 3.144 | 60.34 | 47.49% to 71.91% | 55.17 | 42.45% to 67.25% |
| < 3.157 | 60.34 | 47.49% to 71.91% | 53.45 | 40.80% to 65.67% |
| < 3.162 | 62.07 | 49.20% to 73.44% | 53.45 | 40.80% to 65.67% |
| < 3.175 | 63.79 | 50.93% to 74.95% | 53.45 | 40.80% to 65.67% |
| < 3.187 | 65.52 | 52.67% to 76.44% | 53.45 | 40.80% to 65.67% |
| < 3.200 | 65.52 | 52.67% to 76.44% | 51.72 | 39.16% to 64.07% |
| < 3.244 | 65.52 | 52.67% to 76.44% | 50.00 | 37.54% to 62.46% |
| < 3.281 | 65.52 | 52.67% to 76.44% | 48.28 | 35.93% to 60.84% |
| < 3.296 | 67.24 | 54.42% to 77.92% | 48.28 | 35.93% to 60.84% |
| < 3.324 | 68.97 | 56.20% to 79.38% | 44.83 | 32.75% to 57.55% |
| < 3.350 | 68.97 | 56.20% to 79.38% | 43.10 | 31.18% to 55.88% |
| < 3.407 | 68.97 | 56.20% to 79.38% | 41.38 | 29.63% to 54.20% |
| < 3.459 | 68.97 | 56.20% to 79.38% | 39.66 | 28.09% to 52.51% |
| < 3.464 | 70.69 | 57.99% to 80.82% | 39.66 | 28.09% to 52.51% |
| < 3.469 | 70.69 | 57.99% to 80.82% | 37.93 | 26.56% to 50.80% |
| < 3.495 | 72.41 | 59.80% to 82.25% | 37.93 | 26.56% to 50.80% |
| < 3.521 | 74.14 | 61.62% to 83.65% | 37.93 | 26.56% to 50.80% |
| < 3.538 | 75.86 | 63.47% to 85.04% | 37.93 | 26.56% to 50.80% |
| < 3.614 | 77.59 | 65.34% to 86.41% | 37.93 | 26.56% to 50.80% |
| < 3.692 | 79.31 | 67.23% to 87.75% | 37.93 | 26.56% to 50.80% |
| < 3.721 | 81.03 | 69.15% to 89.07% | 37.93 | 26.56% to 50.80% |
| < 3.776 | 82.76 | 71.09% to 90.36% | 37.93 | 26.56% to 50.80% |
| < 3.829 | 82.76 | 71.09% to 90.36% | 36.21 | 25.05% to 49.07% |
| < 3.858 | 82.76 | 71.09% to 90.36% | 34.48 | 23.56% to 47.33% |
| < 3.904 | 82.76 | 71.09% to 90.36% | 32.76 | 22.08% to 45.58% |
| < 4.074 | 84.48 | 73.07% to 91.62% | 32.76 | 22.08% to 45.58% |
| < 4.233 | 84.48 | 73.07% to 91.62% | 31.03 | 20.62% to 43.80% |
| < 4.282 | 86.21 | 75.07% to 92.84% | 31.03 | 20.62% to 43.80% |
| < 4.470 | 86.21 | 75.07% to 92.84% | 29.31 | 19.18% to 42.01% |
| < 4.686 | 87.93 | 77.12% to 94.03% | 29.31 | 19.18% to 42.01% |
| < 4.835 | 87.93 | 77.12% to 94.03% | 27.59 | 17.75% to 40.20% |
| < 4.968 | 89.66 | 79.21% to 95.17% | 27.59 | 17.75% to 40.20% |
| < 5.123 | 89.66 | 79.21% to 95.17% | 25.86 | 16.35% to 38.38% |
| < 5.449 | 91.38 | 81.36% to 96.26% | 25.86 | 16.35% to 38.38% |
| < 5.691 | 91.38 | 81.36% to 96.26% | 24.14 | 14.96% to 36.53% |
| < 5.722 | 91.38 | 81.36% to 96.26% | 22.41 | 13.59% to 34.66% |
| < 5.732 | 91.38 | 81.36% to 96.26% | 20.69 | 12.25% to 32.77% |
| < 5.752 | 91.38 | 81.36% to 96.26% | 18.97 | 10.93% to 30.85% |
| < 5.870 | 91.38 | 81.36% to 96.26% | 17.24 | 9.644% to 28.91% |
| < 6.004 | 91.38 | 81.36% to 96.26% | 15.52 | 8.384% to 26.93% |
| < 6.109 | 93.10 | 83.57% to 97.29% | 15.52 | 8.384% to 26.93% |
| < 6.274 | 94.83 | 85.86% to 98.59% | 15.52 | 8.384% to 26.93% |
| < 6.383 | 96.55 | 88.27% to 99.39% | 15.52 | 8.384% to 26.93% |
| < 6.504 | 98.28 | 90.86% to 99.91% | 15.52 | 8.384% to 26.93% |
| < 6.688 | 100.0 | 93.79% to 100.0% | 15.52 | 8.384% to 26.93% |
| < 6.829 | 100.0 | 93.79% to 100.0% | 13.79 | 7.158% to 24.93% |
| < 6.932 | 100.0 | 93.79% to 100.0% | 12.07 | 5.971% to 22.88% |
| < 7.005 | 100.0 | 93.79% to 100.0% | 10.34 | 4.828% to 20.79% |
| < 7.099 | 100.0 | 93.79% to 100.0% | 8.621 | 3.739% to 18.64% |
| < 7.273 | 100.0 | 93.79% to 100.0% | 6.897 | 2.714% to 16.43% |

**Sensitivity and specificity analysis from the dataset of 49 vs 49 individuals**

| **Cut-off values** | **Sensitivity (%)** | **95% CI** | **Specificity (%)** | **95% CI** |
| --- | --- | --- | --- | --- |
| < 1.295 | 2.041 | 0.1047% to 10.69% | 100.0 | 92.73% to 100.0% |
| < 1.439 | 4.082 | 0.7252% to 13.71% | 100.0 | 92.73% to 100.0% |
| < 1.473 | 6.122 | 2.104% to 16.52% | 100.0 | 92.73% to 100.0% |
| < 1.502 | 8.163 | 3.220% to 19.19% | 100.0 | 92.73% to 100.0% |
| < 1.571 | 10.20 | 4.438% to 21.76% | 100.0 | 92.73% to 100.0% |
| < 1.635 | 12.24 | 5.735% to 24.24% | 100.0 | 92.73% to 100.0% |
| < 1.674 | 12.24 | 5.735% to 24.24% | 97.96 | 89.31% to 99.90% |
| < 1.791 | 14.29 | 7.096% to 26.67% | 97.96 | 89.31% to 99.90% |
| < 1.887 | 16.33 | 8.513% to 29.04% | 97.96 | 89.31% to 99.90% |
| < 1.908 | 18.37 | 9.976% to 31.36% | 97.96 | 89.31% to 99.90% |
| < 1.916 | 18.37 | 9.976% to 31.36% | 95.92 | 86.29% to 99.27% |
| < 1.921 | 20.41 | 11.48% to 33.64% | 95.92 | 86.29% to 99.27% |
| < 1.937 | 20.41 | 11.48% to 33.64% | 93.88 | 83.48% to 97.90% |
| < 1.977 | 20.41 | 11.48% to 33.64% | 91.84 | 80.81% to 96.78% |
| < 2.020 | 20.41 | 11.48% to 33.64% | 89.80 | 78.24% to 95.56% |
| < 2.069 | 22.45 | 13.02% to 35.88% | 89.80 | 78.24% to 95.56% |
| < 2.108 | 22.45 | 13.02% to 35.88% | 87.76 | 75.76% to 94.27% |
| < 2.125 | 24.49 | 14.60% to 38.09% | 87.76 | 75.76% to 94.27% |
| < 2.172 | 26.53 | 16.21% to 40.26% | 87.76 | 75.76% to 94.27% |
| < 2.221 | 26.53 | 16.21% to 40.26% | 85.71 | 73.33% to 92.90% |
| < 2.239 | 26.53 | 16.21% to 40.26% | 83.67 | 70.96% to 91.49% |
| < 2.246 | 26.53 | 16.21% to 40.26% | 81.63 | 68.64% to 90.02% |
| < 2.278 | 28.57 | 17.85% to 42.41% | 81.63 | 68.64% to 90.02% |
| < 2.309 | 28.57 | 17.85% to 42.41% | 79.59 | 66.36% to 88.52% |
| < 2.320 | 30.61 | 19.52% to 44.53% | 79.59 | 66.36% to 88.52% |
| < 2.340 | 32.65 | 21.21% to 46.62% | 79.59 | 66.36% to 88.52% |
| < 2.367 | 32.65 | 21.21% to 46.62% | 77.55 | 64.12% to 86.98% |
| < 2.393 | 34.69 | 22.92% to 48.69% | 77.55 | 64.12% to 86.98% |
| < 2.430 | 34.69 | 22.92% to 48.69% | 75.51 | 61.91% to 85.40% |
| < 2.470 | 34.69 | 22.92% to 48.69% | 73.47 | 59.74% to 83.79% |
| < 2.485 | 36.73 | 24.67% to 50.73% | 73.47 | 59.74% to 83.79% |
| < 2.490 | 36.73 | 24.67% to 50.73% | 71.43 | 57.59% to 82.15% |
| < 2.501 | 38.78 | 26.43% to 52.75% | 71.43 | 57.59% to 82.15% |
| < 2.511 | 42.86 | 30.02% to 56.73% | 71.43 | 57.59% to 82.15% |
| < 2.514 | 42.86 | 30.02% to 56.73% | 69.39 | 55.47% to 80.48% |
| < 2.538 | 44.90 | 31.85% to 58.68% | 67.35 | 53.38% to 78.79% |
| < 2.576 | 44.90 | 31.85% to 58.68% | 65.31 | 51.31% to 77.08% |
| < 2.607 | 44.90 | 31.85% to 58.68% | 63.27 | 49.27% to 75.33% |
| < 2.651 | 46.94 | 33.70% to 60.62% | 63.27 | 49.27% to 75.33% |
| < 2.688 | 48.98 | 35.58% to 62.53% | 63.27 | 49.27% to 75.33% |
| < 2.717 | 48.98 | 35.58% to 62.53% | 61.22 | 47.25% to 73.57% |
| < 2.770 | 48.98 | 35.58% to 62.53% | 59.18 | 45.25% to 71.78% |
| < 2.826 | 48.98 | 35.58% to 62.53% | 57.14 | 43.27% to 69.98% |
| < 2.906 | 51.02 | 37.47% to 64.42% | 57.14 | 43.27% to 69.98% |
| < 2.990 | 53.06 | 39.38% to 66.30% | 57.14 | 43.27% to 69.98% |
| < 3.043 | 55.10 | 41.32% to 68.15% | 57.14 | 43.27% to 69.98% |
| < 3.099 | 57.14 | 43.27% to 69.98% | 57.14 | 43.27% to 69.98% |
| < 3.142 | 59.18 | 45.25% to 71.78% | 57.14 | 43.27% to 69.98% |
| < 3.159 | 59.18 | 45.25% to 71.78% | 55.10 | 41.32% to 68.15% |
| < 3.175 | 61.22 | 47.25% to 73.57% | 55.10 | 41.32% to 68.15% |
| < 3.187 | 63.27 | 49.27% to 75.33% | 55.10 | 41.32% to 68.15% |
| < 3.231 | 63.27 | 49.27% to 75.33% | 53.06 | 39.38% to 66.30% |
| < 3.281 | 63.27 | 49.27% to 75.33% | 51.02 | 37.47% to 64.42% |
| < 3.296 | 65.31 | 51.31% to 77.08% | 51.02 | 37.47% to 64.42% |
| < 3.324 | 67.35 | 53.38% to 78.79% | 46.94 | 33.70% to 60.62% |
| < 3.350 | 67.35 | 53.38% to 78.79% | 44.90 | 31.85% to 58.68% |
| < 3.407 | 67.35 | 53.38% to 78.79% | 42.86 | 30.02% to 56.73% |
| < 3.459 | 67.35 | 53.38% to 78.79% | 40.82 | 28.22% to 54.75% |
| < 3.464 | 69.39 | 55.47% to 80.48% | 40.82 | 28.22% to 54.75% |
| < 3.469 | 69.39 | 55.47% to 80.48% | 38.78 | 26.43% to 52.75% |
| < 3.495 | 71.43 | 57.59% to 82.15% | 38.78 | 26.43% to 52.75% |
| < 3.521 | 73.47 | 59.74% to 83.79% | 38.78 | 26.43% to 52.75% |
| < 3.538 | 75.51 | 61.91% to 85.40% | 38.78 | 26.43% to 52.75% |
| < 3.630 | 77.55 | 64.12% to 86.98% | 38.78 | 26.43% to 52.75% |
| < 3.721 | 79.59 | 66.36% to 88.52% | 38.78 | 26.43% to 52.75% |
| < 3.776 | 81.63 | 68.64% to 90.02% | 38.78 | 26.43% to 52.75% |
| < 3.847 | 81.63 | 68.64% to 90.02% | 36.73 | 24.67% to 50.73% |
| < 3.904 | 81.63 | 68.64% to 90.02% | 34.69 | 22.92% to 48.69% |
| < 4.074 | 83.67 | 70.96% to 91.49% | 34.69 | 22.92% to 48.69% |
| < 4.265 | 83.67 | 70.96% to 91.49% | 32.65 | 21.21% to 46.62% |
| < 4.470 | 83.67 | 70.96% to 91.49% | 30.61 | 19.52% to 44.53% |
| < 4.686 | 85.71 | 73.33% to 92.90% | 30.61 | 19.52% to 44.53% |
| < 4.835 | 85.71 | 73.33% to 92.90% | 28.57 | 17.85% to 42.41% |
| < 5.079 | 87.76 | 75.76% to 94.27% | 28.57 | 17.85% to 42.41% |
| < 5.449 | 89.80 | 78.24% to 95.56% | 28.57 | 17.85% to 42.41% |
| < 5.691 | 89.80 | 78.24% to 95.56% | 26.53 | 16.21% to 40.26% |
| < 5.722 | 89.80 | 78.24% to 95.56% | 24.49 | 14.60% to 38.09% |
| < 5.732 | 89.80 | 78.24% to 95.56% | 22.45 | 13.02% to 35.88% |
| < 5.752 | 89.80 | 78.24% to 95.56% | 20.41 | 11.48% to 33.64% |
| < 5.870 | 89.80 | 78.24% to 95.56% | 18.37 | 9.976% to 31.36% |
| < 6.004 | 89.80 | 78.24% to 95.56% | 16.33 | 8.513% to 29.04% |
| < 6.109 | 91.84 | 80.81% to 96.78% | 16.33 | 8.513% to 29.04% |
| < 6.274 | 93.88 | 83.48% to 97.90% | 16.33 | 8.513% to 29.04% |
| < 6.383 | 95.92 | 86.29% to 99.27% | 16.33 | 8.513% to 29.04% |
| < 6.504 | 97.96 | 89.31% to 99.90% | 16.33 | 8.513% to 29.04% |
| < 6.688 | 100.0 | 92.73% to 100.0% | 16.33 | 8.513% to 29.04% |
| < 6.829 | 100.0 | 92.73% to 100.0% | 14.29 | 7.096% to 26.67% |
| < 6.932 | 100.0 | 92.73% to 100.0% | 12.24 | 5.735% to 24.24% |
| < 7.005 | 100.0 | 92.73% to 100.0% | 10.20 | 4.438% to 21.76% |
| < 7.099 | 100.0 | 92.73% to 100.0% | 8.163 | 3.220% to 19.19% |
| < 7.217 | 100.0 | 92.73% to 100.0% | 6.122 | 2.104% to 16.52% |

**Linear regression analyses of the CAG repeat length of *ATXN2* expanded alleles and SERPINB1 plasma levels on the age at disease onset (AO)**

| Regression model | Dependent variable | Predictors | β (SE) | t (p) | R | R^2^ | R^2^_adj_ |
| --- | --- | --- | --- | --- | --- | --- | --- |
| 1 | AO | *ATXN2* expanded alleles | -0.203 (0.021) | -9.505 (<0.001) | 0.805 | 0.648 | 0.641 |
| 2 | AO | *ATXN2* expanded alleles | -0.189 (0.021) | -8.821 (<0.001) | 0.825 | 0.680 | 0.667 |
|  |  | SERPINB1 plasma levels | -1.553 (0.710) | -2.187 (0.034) |  |  |  |

These results from linear regression analyses indicated that 2.6% of the total variability in the age at onset might be attributable to the variations in SERPINB1 plasma levels. Thus, beyond the CAG expansion effect some 7.24% of the unexplained variation in age at onset might be reflected by variation at SERPINB1 plasma levels.

| **Covariance analysis for SERPINB1 in the whole dataset of cases and controls (58vs58)**  Covariance analyses were based on the age, sex, group, and highSERPINB1 as covariates. The CAG expansion was not included because it only happens in the patients group.  1-Baseline ANCOVA   \| **Tests of Between-Subjects Effects** \| \| \| \| \| \| \| \| \| \| \| \| \| --- \| --- \| --- \| --- \| --- \| --- \| --- \| --- \| --- \| --- \| --- \| --- \| \| Dependent Variable: serpinb1 \| \| \| \| \| \| \| \| \| \| \| \| \| Source \| \| Type III Sum of Squares \| \| df \| \| Mean Square \| \| \| F \| Sig. \| \| \| Corrected Model \| \| 37,851^a^ \| \| 3 \| \| 12,617 \| \| \| 2,989 \| ,034 \| \| \| Intercept \| \| 24,576 \| \| 1 \| \| 24,576 \| \| \| 5,822 \| ,017 \| \| \| **Group** \| \| 32,726 \| \| 1 \| \| 32,726 \| \| \| 7,752 \| **,006** \| \| \| **Age** \| \| 4,760 \| \| 1 \| \| 4,760 \| \| \| 1,128 \| ,291 \| \| \| **Sex** \| \| ,846 \| \| 1 \| \| ,846 \| \| \| ,200 \| ,655 \| \| \| Error \| \| 472,810 \| \| 112 \| \| 4,222 \| \| \|  \|  \| \| \| Total \| \| 1886,119 \| \| 116 \| \|  \| \| \|  \|  \| \| \| Corrected Total \| \| 510,661 \| \| 115 \| \|  \| \| \|  \|  \| \| \| a. R Squared = ,074 (Adjusted R Squared = ,049) \| \| \| \| \| \| \| \| \| \| \| \| \| **Parameter Estimates** \| \| \| \| \| \| \| \| \| \| \| \| \| \| Dependent Variable: serpinb1 \| \| \| \| \| \| \| \| \| \| \| \| \| \| Parameter \| B \| \| Std. Error \| \| t \| \| Sig. \| 95% Confidence Interval \| \| \| \| \| \| Lower Bound \| \| \| Upper Bound \| \| \| Intercept \| 2,368 \| \| 1,212 \| \| 1,953 \| \| ,053 \| -,035 \| \| \| 4,770 \| \| \| [status=Ctrl] \| 1,069 \| \| ,384 \| \| 2,784 \| \| **,006** \| ,308 \| \| \| 1,830 \| \| \| [status=SCA2] \| 0^a^ \| \| . \| \| . \| \| . \| . \| \| \| . \| \| \| Age \| ,017 \| \| ,016 \| \| 1,062 \| \| ,291 \| -,015 \| \| \| ,048 \| \| \| Sex \| -,186 \| \| ,415 \| \| -,448 \| \| ,655 \| -1,009 \| \| \| ,637 \| \| \| a. This parameter is set to zero because it is redundant. \| \| \| \| \| \| \| \| \| \| \| \| \|   This analysis confirmed that significant differences appeared for SERPINB1 plasma levels between patients and controls.  2-ANCOVA including an indicator variable (highSERPINB1) for control high (> 5.5 ng/mL) SERPINB1 plasma levels   \| **Tests of Between-Subjects Effects** \| \| \| \| \| \| \| \| \| \| \| \| --- \| --- \| --- \| --- \| --- \| --- \| --- \| --- \| --- \| --- \| --- \| \| Dependent Variable: serpinb1 \| \| \| \| \| \| \| \| \| \| \| \| Source \| Type III Sum of Squares \| \| df \| \| Mean Square \| \| \| F \| \| Sig. \| \| Corrected Model \| 348,795^a^ \| \| 4 \| \| 87,199 \| \| \| 59,797 \| \| ,000 \| \| Intercept \| 52,003 \| \| 1 \| \| 52,003 \| \| \| 35,661 \| \| ,000 \| \| **Age** \| 5,908 \| \| 1 \| \| 5,908 \| \| \| 4,051 \| \| ,094 \| \| **Sex** \| ,809 \| \| 1 \| \| ,809 \| \| \| ,555 \| \| ,458 \| \| **Group** \| 2,512 \| \| 1 \| \| 2,512 \| \| \| 1,723 \| \| ,192 \| \| **highSERPINB1** \| 310,944 \| \| 1 \| \| 310,944 \| \| \| 213,230 \| \| **,000** \| \| Error \| 161,866 \| \| 111 \| \| 1,458 \| \| \|  \| \|  \| \| Total \| 1886,119 \| \| 116 \| \|  \| \| \|  \| \|  \| \| Corrected Total \| 510,661 \| \| 115 \| \|  \| \| \|  \| \|  \| \| a. R Squared = ,683 (Adjusted R Squared = ,672) \| \| \| \| \| \| \| \| \| \| \| \| **Parameter Estimates** \| \| \| \| \| \| \| \| \| \| \| \| \| \| Dependent Variable: serpinb1 \| \| \| \| \| \| \| \| \| \| \| \| \| \| Parameter \| \| B \| \| Std. Error \| \| t \| Sig. \| \| 95% Confidence Interval \| \| \| \| \| Lower Bound \| \| \| Upper Bound \| \| Intercept \| \| 6,332 \| \| ,763 \| \| 8,303 \| ,000 \| \| 4,821 \| \| \| 7,843 \| \| age \| \| ,019 \| \| ,009 \| \| 2,013 \| ,094 \| \| ,000 \| \| \| ,037 \| \| sex \| \| -,182 \| \| ,244 \| \| -,745 \| ,458 \| \| -,666 \| \| \| ,302 \| \| [group=Ctrl] \| \| ,304 \| \| ,232 \| \| 1,313 \| ,192 \| \| -,155 \| \| \| ,763 \| \| [group=SCA2] \| \| 0^a^ \| \| . \| \| . \| . \| \| . \| \| \| . \| \| [highSERPINB1=No] \| \| -4,452 \| \| ,305 \| \| -14,602 \| **,000** \| \| -5,056 \| \| \| -3,848 \| \| [highSERPINB1=Yes] \| \| 0^a^ \| \| . \| \| . \| . \| \| . \| \| \| . \| \| a. This parameter is set to zero because it is redundant. \| \| \| \| \| \| \| \| \| \| \| \| \|   This analysis shows that the significant effect of the “group” on SERPINB1 plasma levels observed in the baseline ANCOVA, is lost after the inclusion of a variable (highSERPINB1) controlling for cases with > 5.5 ng/mL SERPINB1 plasma levels. | |
| --- | --- | --- | --- | --- | --- | --- | --- | --- | --- | --- | --- | --- | --- | --- | --- | --- | --- | --- | --- | --- | --- | --- | --- | --- | --- | --- | --- | --- | --- | --- | --- | --- | --- | --- | --- | --- | --- | --- | --- | --- | --- | --- | --- | --- | --- | --- | --- | --- | --- | --- | --- | --- | --- | --- | --- | --- | --- | --- | --- | --- | --- | --- | --- | --- | --- | --- | --- | --- | --- | --- | --- | --- | --- | --- | --- | --- | --- | --- | --- | --- | --- | --- | --- | --- | --- | --- | --- | --- | --- | --- | --- | --- | --- | --- | --- | --- | --- | --- | --- | --- | --- | --- | --- | --- | --- | --- | --- | --- | --- | --- | --- | --- | --- | --- | --- | --- | --- | --- | --- | --- | --- | --- | --- | --- | --- | --- | --- | --- | --- | --- | --- | --- | --- | --- | --- | --- | --- | --- | --- | --- | --- | --- | --- | --- | --- | --- | --- | --- | --- | --- | --- | --- | --- | --- | --- | --- | --- | --- | --- | --- | --- | --- | --- | --- | --- | --- | --- | --- | --- | --- | --- | --- | --- | --- | --- | --- | --- | --- | --- | --- | --- | --- | --- | --- | --- | --- | --- | --- | --- | --- | --- | --- | --- | --- | --- | --- | --- | --- | --- | --- | --- | --- | --- | --- | --- | --- | --- | --- | --- | --- | --- | --- | --- | --- | --- | --- | --- | --- | --- | --- | --- | --- | --- | --- | --- | --- | --- | --- | --- | --- | --- | --- | --- | --- | --- | --- | --- | --- | --- | --- | --- | --- | --- | --- | --- | --- | --- | --- | --- | --- | --- | --- | --- | --- | --- | --- | --- | --- | --- | --- | --- | --- | --- | --- | --- | --- | --- | --- | --- | --- | --- | --- | --- | --- | --- | --- | --- | --- | --- | --- | --- | --- | --- | --- | --- | --- | --- | --- | --- | --- | --- | --- | --- | --- | --- | --- | --- | --- | --- | --- | --- | --- | --- | --- | --- | --- | --- | --- | --- | --- | --- | --- | --- | --- | --- | --- | --- | --- | --- | --- | --- | --- | --- | --- | --- | --- | --- | --- | --- | --- | --- | --- | --- | --- | --- | --- | --- | --- | --- | --- | --- | --- | --- | --- | --- | --- | --- | --- | --- | --- | --- | --- | --- | --- | --- | --- | --- | --- | --- | --- | --- | --- | --- | --- | --- | --- | --- | --- | --- | --- | --- | --- | --- | --- | --- | --- | --- | --- | --- | --- | --- | --- | --- | --- | --- | --- | --- | --- | --- | --- | --- | --- | --- | --- | --- | --- | --- | --- | --- | --- | --- | --- | --- | --- | --- | --- | --- | --- | --- | --- | --- | --- | --- | --- | --- | --- | --- | --- | --- | --- | --- | --- | --- | --- | --- | --- | --- | --- | --- | --- | --- | --- | --- | --- | --- | --- | --- | --- | --- | --- | --- | --- | --- | --- | --- | --- | --- | --- | --- | --- | --- | --- | --- | --- | --- | --- | --- | --- | --- | --- | --- | --- | --- | --- | --- | --- | --- | --- | --- | --- | --- | --- | --- | --- | --- | --- | --- | --- | --- | --- | --- | --- | --- | --- | --- | --- | --- | --- | --- | --- | --- | --- | --- | --- | --- | --- | --- | --- | --- | --- | --- | --- | --- | --- | --- | --- | --- | --- | --- | --- | --- | --- | --- | --- | --- | --- | --- | --- | --- | --- | --- | --- | --- | --- | --- | --- | --- | --- | --- | --- | --- | --- | --- | --- | --- | --- | --- | --- | --- | --- | --- | --- | --- | --- | --- | --- | --- | --- | --- | --- | --- | --- | --- | --- | --- | --- | --- |
| 3- ANCOVA including the interaction of an indicator variable (highSERPINB1) for control high (> 5.5 ng/mL) SERPINB1 plasma levels with the group (patients vs controls)   \| **Tests of Between-Subjects Effects** \| \| \| \| \| \| \| --- \| --- \| --- \| --- \| --- \| --- \| \| Dependent Variable: SERPINB1 \| \| \| \| \| \| \| Source \| Type III Sum of Squares \| df \| Mean Square \| F \| Sig. \| \| Corrected Model \| 351,879^a^ \| 5 \| 70,376 \| 48,755 \| ,000 \| \| Intercept \| 45,323 \| 1 \| 45,323 \| 31,399 \| ,000 \| \| **age** \| 6,192 \| 1 \| 6,192 \| 4,290 \| ,101 \| \| **sex** \| ,451 \| 1 \| ,451 \| ,312 \| ,577 \| \| **group * highSERPINB1** \| 346,754 \| 3 \| 115,585 \| 80,074 \| **,000** \| \| Error \| 158,782 \| 110 \| 1,443 \|  \|  \| \| Total \| 1886,119 \| 116 \|  \|  \|  \| \| Corrected Total \| 510,661 \| 115 \|  \|  \|  \| \| a. R Squared = ,689 (Adjusted R Squared = ,675) \| \| \| \| \| \|  \| **Parameter Estimates** \| \| \| \| \| \| \| \| --- \| --- \| --- \| --- \| --- \| --- \| --- \| \| Dependent Variable: SERPINB1 \| \| \| \| \| \| \| \| Parameter \| B \| Std. Error \| t \| Sig. \| 95% Confidence Interval \| \| \| Lower Bound \| Upper Bound \| \| Intercept \| 5,601 \| ,909 \| 6,164 \| ,000 \| 3,800 \| 7,402 \| \| Age \| ,019 \| ,009 \| 2,071 \| ,101 \| ,001 \| ,037 \| \| Sex \| -,137 \| ,245 \| -,559 \| ,577 \| -,622 \| ,348 \| \| [status=Ctrl] * [highSERPINB1=No] \| -3,589 \| ,568 \| -6,314 \| **,000** \| -4,715 \| -2,463 \| \| [status=Ctrl] * [highSERPINB1=Yes] \| 1,150 \| ,623 \| 1,846 \| ,068 \| -,084 \| 2,384 \| \| [status=SCA2] * [highSERPINB1=No] \| -3,755 \| ,565 \| -6,643 \| **,000** \| -4,875 \| -2,635 \| \| [status=SCA2] * [highSERPINB1=Yes] \| 0^a^ \| . \| . \| . \| . \| . \| \| a. This parameter is set to zero because it is redundant. \| \| \| \| \| \| \| |  |
| This analysis shows that the interaction between the “group” and highSERPINB1 has a significant impact on SERPINB1 plasma levels. |  |

ANCOVA

| **Tests of Between-Subjects Effects** | | | | | |
| --- | --- | --- | --- | --- | --- |
| Dependent Variable: SERPINB1 | | | | | |
| Source | Type III Sum of Squares | df | Mean Square | F | Sig. |
| Corrected Model | 277,788^a^ | 6 | 46,298 | 23,881 | ,000 |
| Intercept | 16,576 | 1 | 16,576 | 8,550 | ,004 |
| **Group** | ,137 | 1 | ,137 | ,071 | ,791 |
| **Age** | 2,121 | 1 | 2,121 | 1,094 | ,299 |
| **Sex** | 3,436 | 1 | 3,436 | 1,772 | ,187 |
| **CAG repeat** | 4,425 | 1 | 4,425 | 2,282 | ,135 |
| **CtrlHighSERPINB1** | 245,079 | 1 | 245,079 | 126,413 | ,000 |
| **Group * CAG repeat** | ,916 | 1 | ,916 | ,472 | ,494 |
| Error | 157,036 | 81 | 1,939 |  |  |
| Total | 1622,826 | 88 |  |  |  |
| Corrected Total | 434,824 | 87 |  |  |  |
| a. R Squared = ,639 (Adjusted R Squared = ,612) | | | | | |
