# Supplementary material for: Multiomics approach identifies SERPINB1 as candidate biomarker for spinocerebellar ataxia type 2
Source: Sci Rep. 2025 Nov 26;15:42559. doi: 10.1038/s41598-025-29070-7 (PMC12663351; doi:10.1038/s41598-025-29070-7)
Supplement: Supplementary file 26 — Supplementary Material 26 [file 41598_2025_29070_MOESM26_ESM.docx]

***Supplementary Material***

**Figure S1.** Volcano plots showing transcriptionally dysregulated translation-related factors in the cerebellum **(A, B)** and spinal cord **(C, D)** of pre-manifest (10-week-old) **(A, C)** or end-stage (14-month-old) **(B, D)** *Atxn2*-CAG100-KnockIn mice. Down- and upregulated transcripts with at least 1.2-fold expression levels changes in each direction reaching nominal significance in each dataset were colored in green and red, respectively. Among upregulated transcripts, only representative factors with more prominent dysregulations were labelled. Factors with higher than 2.0-fold dysregulations in each direction are shown in bold.

**Figure S2.** Volcano plots showing transcriptionally dysregulated stress granule and RNA processing factors in the cerebellum (A, B) and spinal cord (C, D) of pre-manifest (10-week-old) (A, C) or end-stage (14-month-old) (B, D) Atxn2-CAG100-KnockIn mice. Down- and upregulated transcripts with at least 1.2-fold expression levels changes in each direction reaching nominal significance in each dataset were colored in green and red, respectively. Only factors with more prominent dysregulations (FC 1.5 in each direction) were labelled. Factors with higher than 2.0-fold dysregulations in each direction are shown in bold.

**Figure S3.** Volcano plots showing transcriptionally dysregulated factors of the Autophagy- Lysosome pathway in the cerebellum **(A, B)** and spinal cord **(C, D)** of pre-manifest (10-week-old) **(A, C)** or end-stage (14-month-old) **(B, D)** *Atxn2*-CAG100-KnockIn mice. Down- and upregulated transcripts with at least 1.2-fold expression levels changes in each direction reaching nominal significance in each dataset were colored in green and red, respectively. Only factors with more prominent dysregulations (FC 1.5 in each direction) were labelled. Factors with higher than 2.0-fold dysregulations in each direction are shown in bold, and those with higher than 3.0-fold dysregulations were underlined.

**Figure S4.** Quantitative reverse-transcriptase real-time PCR (N = 6 vs. 6) for transcripts encoding cathepsin proteinases as the main mammalian lysosomal proteases, in end-stage cerebellum **(A)** and spinal cord **(B)** *Atxn2*-CAG100-KnockIn mice. Error bars are depicted as ±SEM. * p < 0.05; ** p < 0.01, *** p < 0.001, **** p < 0.0001, ns = not significant.

**Figure S5.** Quantitative immunoblots **(A, C)** and quantitative reverse-transcriptase real-time PCR **(B, D)** showing consistent downregulation of KLK6/*Klk6* in the cerebellum **(A, B)** and spinal cord **(C, D)** of 10-week-old (pre-manifest), six-month-old (early ataxic stage), and 14-month-old (late ataxic stage) *Atxn2*-CAG100-KIN mice. w = week, m = month. The blots were cropped and full-length raw blots are provided in Supplementary Figure S15. Error bars are depicted as ±SEM. T < 0.10; *p < 0.05; ** p < 0.01, *** p < 0.001.

**Figure S6.** Commonly dysregulated factors in pre-manifest and end-stage *Atxn2*-CAG100-KnockIn mice, based on FC ≥1.5 in each direction, and nominal significance (p<0.05). Venn diagrams of the up- **(A)** and down-regulated **(B)** transcripts in the cerebellar (T_Cbll) and spinal cord transcriptomes (T_SC), and cerebellar proteome (P_Cbll) of pre-manifest KnockIn mice. Venn diagrams of the up- **(C)** or down-regulated **(D)** transcripts/proteins in the cerebellar transcriptome (T_Cbll) and proteome (P_Cbll), and spinal cord transcriptome (T_SC), proteome (P_SC), and spinal cord phosphoproteome (PP_SC) of end-stage KnockIn mice.

**Figure S7.** Volcano plots showing transcriptionally dysregulated Serpins in the cerebellum **(A, B)** and spinal cord **(C, D)** of pre-manifest (10-week-old) **(A, C)** or end-stage (14-month-old) **(B, D)** *Atxn2*-CAG100-KnockIn mice. Down- and upregulated transcripts reaching nominal significance in each dataset were labeled and colored in green and red, respectively. Dashed lines represent the cutoff p-value for nominal significance (-log10 p-value = 1.3).

**Figure S8.** Quantitative reverse-transcriptase real-time PCR (N = 5 vs. 5) for transcripts encoding serpin protease inhibitors, in the cerebellum **(A, C, E)** and spinal cord **(B, D, F)** of *Atxn2*-CAG100-KnockIn mice. Experiments in **A** and **B** were conducted in end-stage mice (14-month-old), while experiments in **C-F** were conducted in pre-manifest (10-week-old) and end-stage mice (14-month-old) mice. Error bars are depicted as ±SEM. * p < 0.05; ** p < 0.01, *** p < 0.001, **** p < 0.0001, ns = not significant.

**Figure S9.** Distribution of the CAG repeat length at expanded *ATXN2* alleles in the extended cohort of Cuban patients with SCA2.

**Figure S10.** Distribution of the CAG repeat length at expanded *ATXN2* alleles across disease stages in the extended cohort of Cuban patients with SCA2.

**Figure S11.** Trimmed mean analyses for SERPINB1 plasma levels in SCA2 patients and control individuals. Bar charts and connected dot plots for symmetric 10% **(A, D)**, 20% **(B, E)**, and asymmetric **(C, F)** trimmed means in the data set of sex- and age-matched 58 patients with SCA2 and 58 control individuals. Bar charts and connected dot plots for patients and controls **(G, J)**, symmetric 10% **(H, K)**, and asymmetric **(I, L)** trimmed means in the data set of perfectly sex- and age-matched 49 patients with SCA2 and 49 control individuals. Excluded data points are marked in grey color.

**Figure S12.** Scatter plots and normal Q-Q plots for the association between SERPINB1 plasma levels and markers of clinical severity in the extended cohort of Cuban patients with SCA2 **(A)** and after removal of outliers **(B)**.

**Figure S13.** Distribution of the SERPINB1 plasma levels across disease stages in the extended cohort of Cuban patients with SCA2.

**Figure S14.** Full-length raw immunoblots for SERPINB1A/ACTB in the cerebellum and spinal cord of 10-week-old (pre-manifest), 6-month-old (early ataxic stage), and 14-month-old (late ataxic stage) *Atxn2*-CAG100-KIN mice.

**Figure S15.** Full-length raw immunoblots for KLK6/GAPDH in the cerebellum and spinal cord of 14-month-old (late ataxic stage) *Atxn2*-CAG100-KIN mice.

**Table S1.** Comparison of SCA2 patients with our SCA2 mouse model, regarding longitudinal course of microscopic underpinnings of motor deficits, neurochemical and weight anomalies.

**Table S2.** List of transcriptionally dysregulated translation-related factors in the cerebellum and spinal cord of pre-manifest (10-week-old) or end-stage (14-month-old) *Atxn2*-CAG100-KnockIn mice. Down- and upregulated transcripts with at least 1.2-fold expression levels changes in each direction reaching nominal significance in each dataset are shown.

**Table S3.** List of transcriptionally dysregulated translation, stress granules, RNA processing, and autophago-lysosome pathway factors progressing over *Atxn2*-CAG100-KnockIn lifespan. Candidate progression markers were selected from global transcriptome profiles upon ≥1.5-fold change at 14 months of age, preceded by also significant >1.2-fold expression change at 10 weeks of age, in *Atxn2*-CAG100-KIN cerebellar and cervicothoracic spinal cord tissue.

**Table S4.** List of transcriptionally dysregulated stress granules and RNA processing factors in the cerebellum and spinal cord of pre-manifest (10-week-old) or end-stage (14-month-old) *Atxn2*-CAG100-KnockIn mice. Down- and upregulated transcripts with at least 1.2-fold expression levels changes in each direction reaching nominal significance in each dataset are shown.

**Table S5.** List of transcriptionally dysregulated autophago-lysosome factors in the cerebellum and spinal cord of pre-manifest (10-week-old) or end-stage (14-month-old) *Atxn2*-CAG100-KnockIn mice. Down- and upregulated transcripts with at least 1.2-fold expression levels changes in each direction reaching nominal significance in each dataset are shown.

**Table S6.** List of commonly dysregulated factors across omics datasets.

**Table S7.** List of transcriptionally dysregulated serpins in the cerebellum and spinal cord of pre-manifest (10-week-old) or end-stage (14-month-old) *Atxn2*-CAG100-KnockIn mice. Down- and upregulated transcripts with at least 1.2-fold expression levels changes in each direction reaching nominal significance in each dataset are shown.

**Table S8.** Clinical and molecular characteristics of the sex- and age-matched SCA2 patients and control individuals.

**Table S9.** Clinical and molecular characteristics of the extended cohort of patients with SCA2.

**Figure S1**


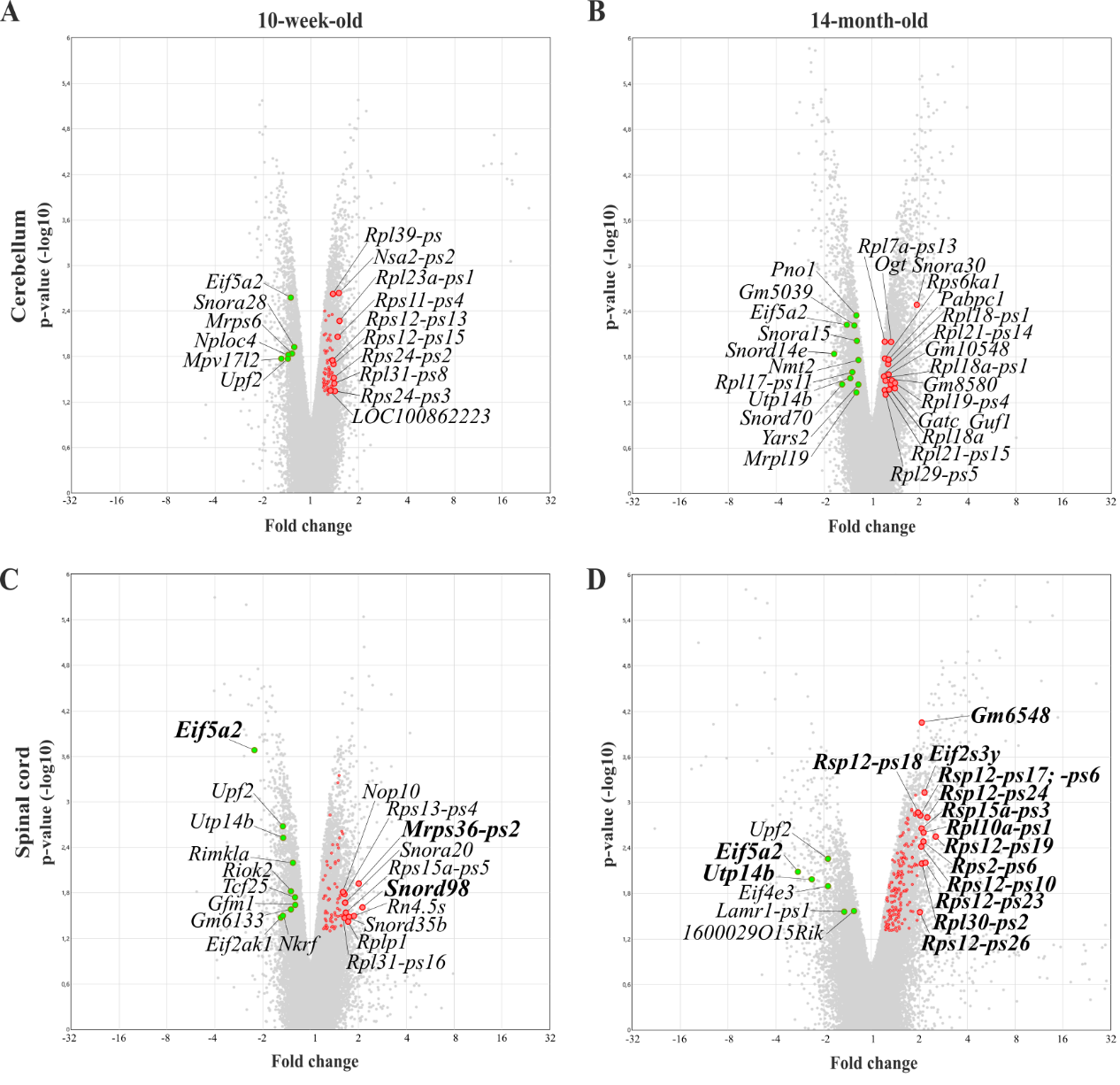


**Figure S2**


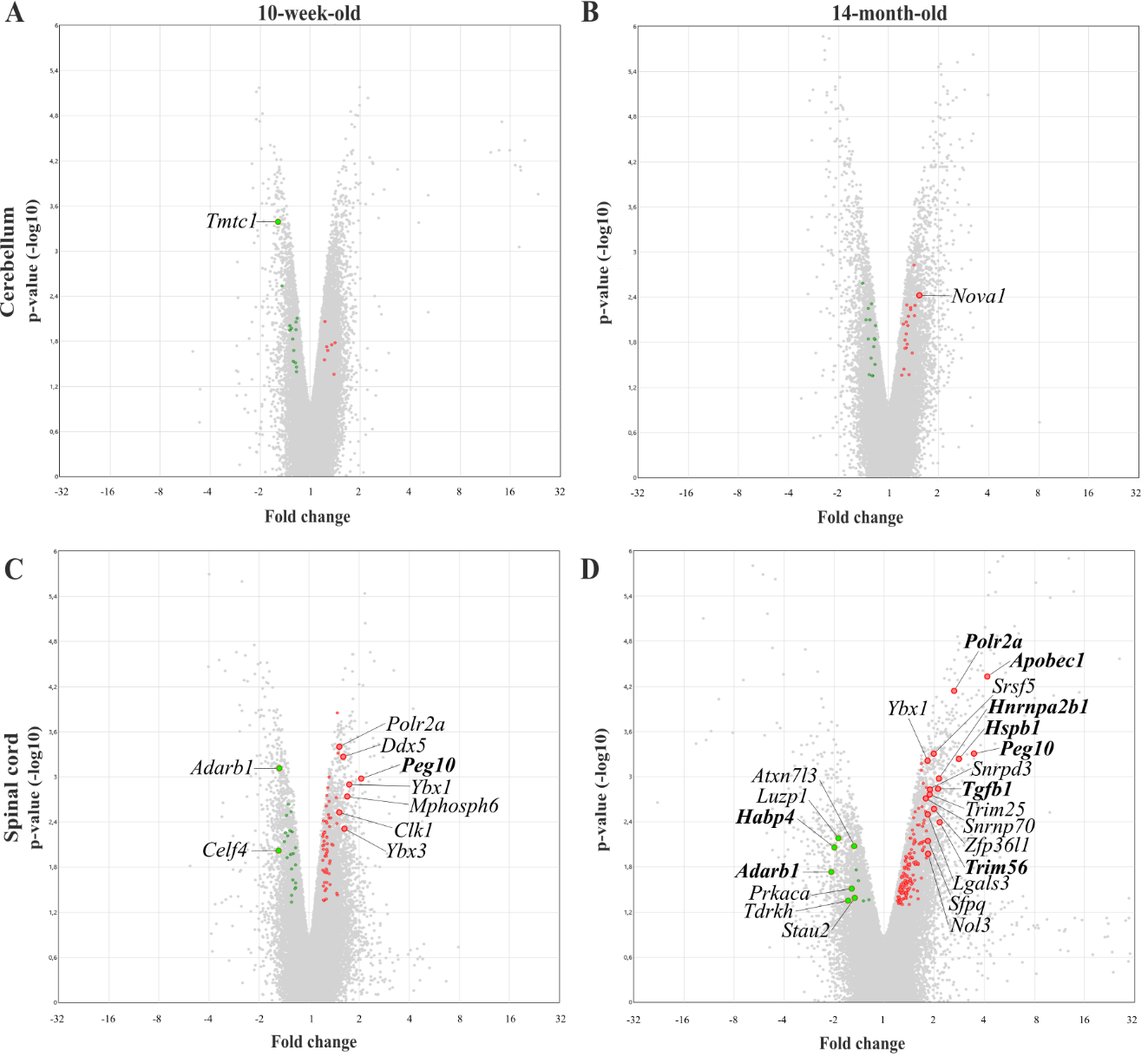


**Figure S3**


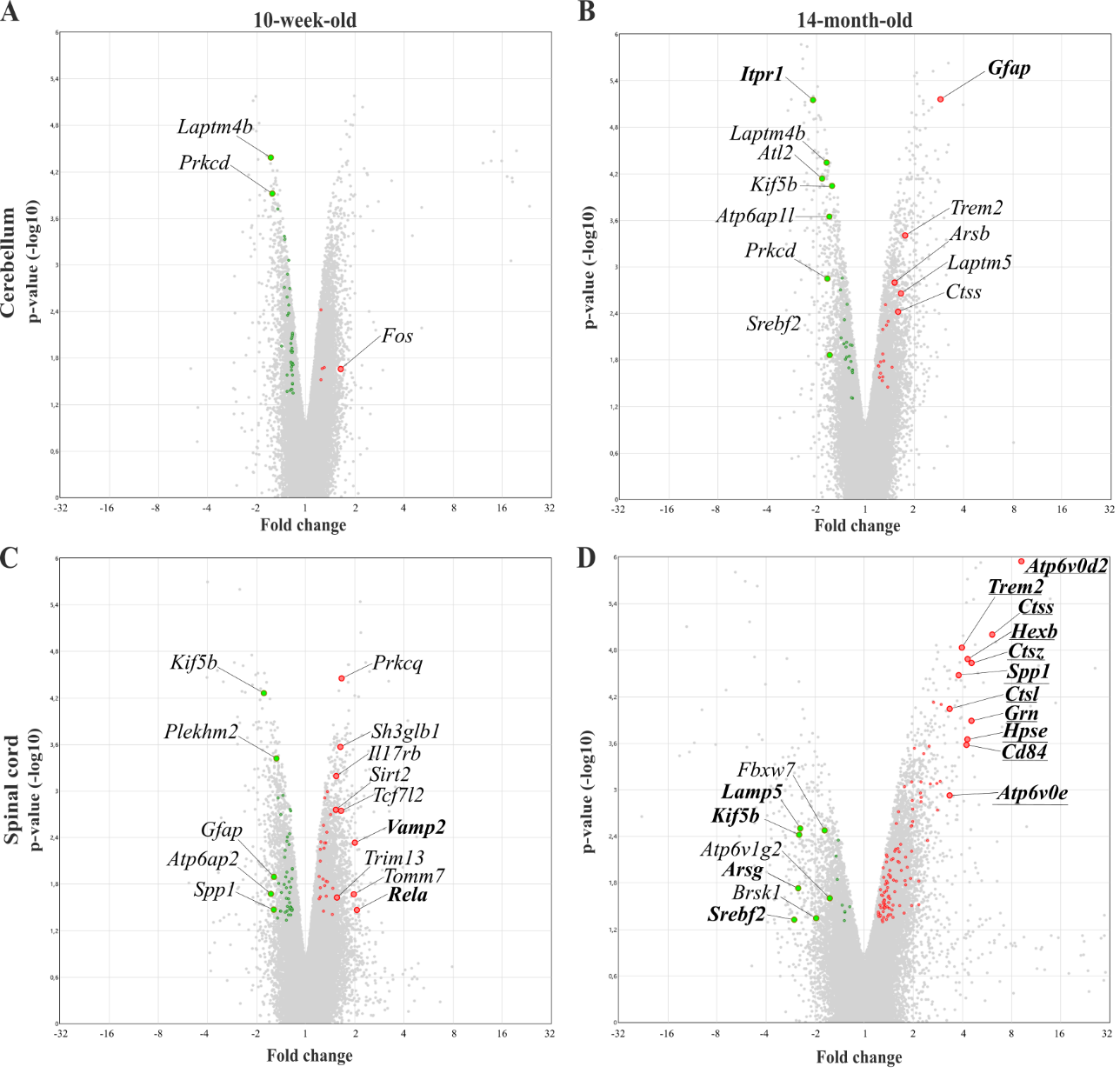


**Figure S4**


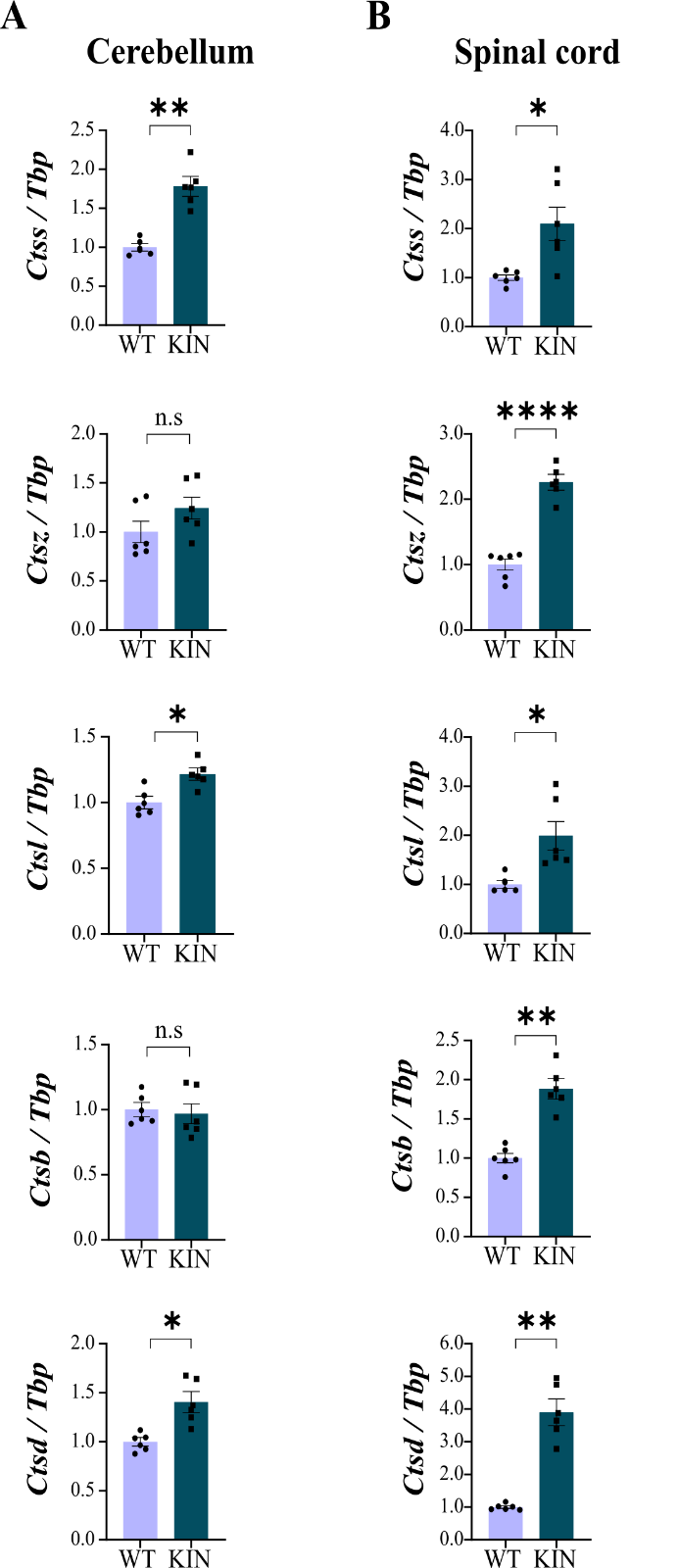


**Figure S5**


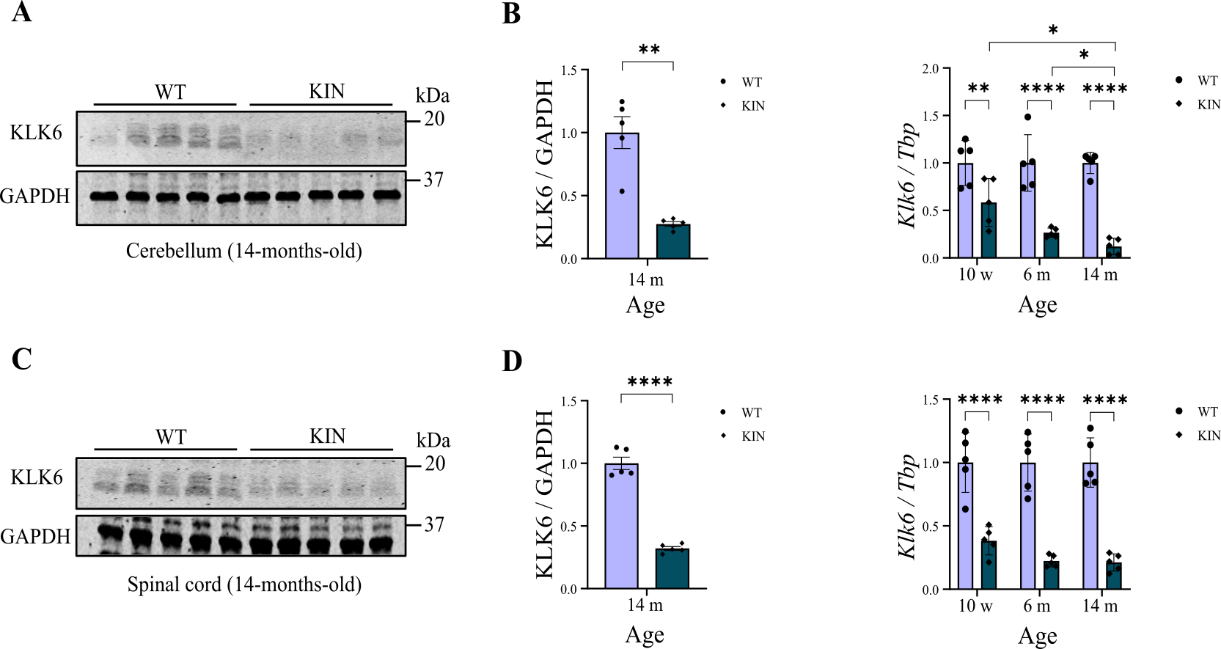


**Figure S6**


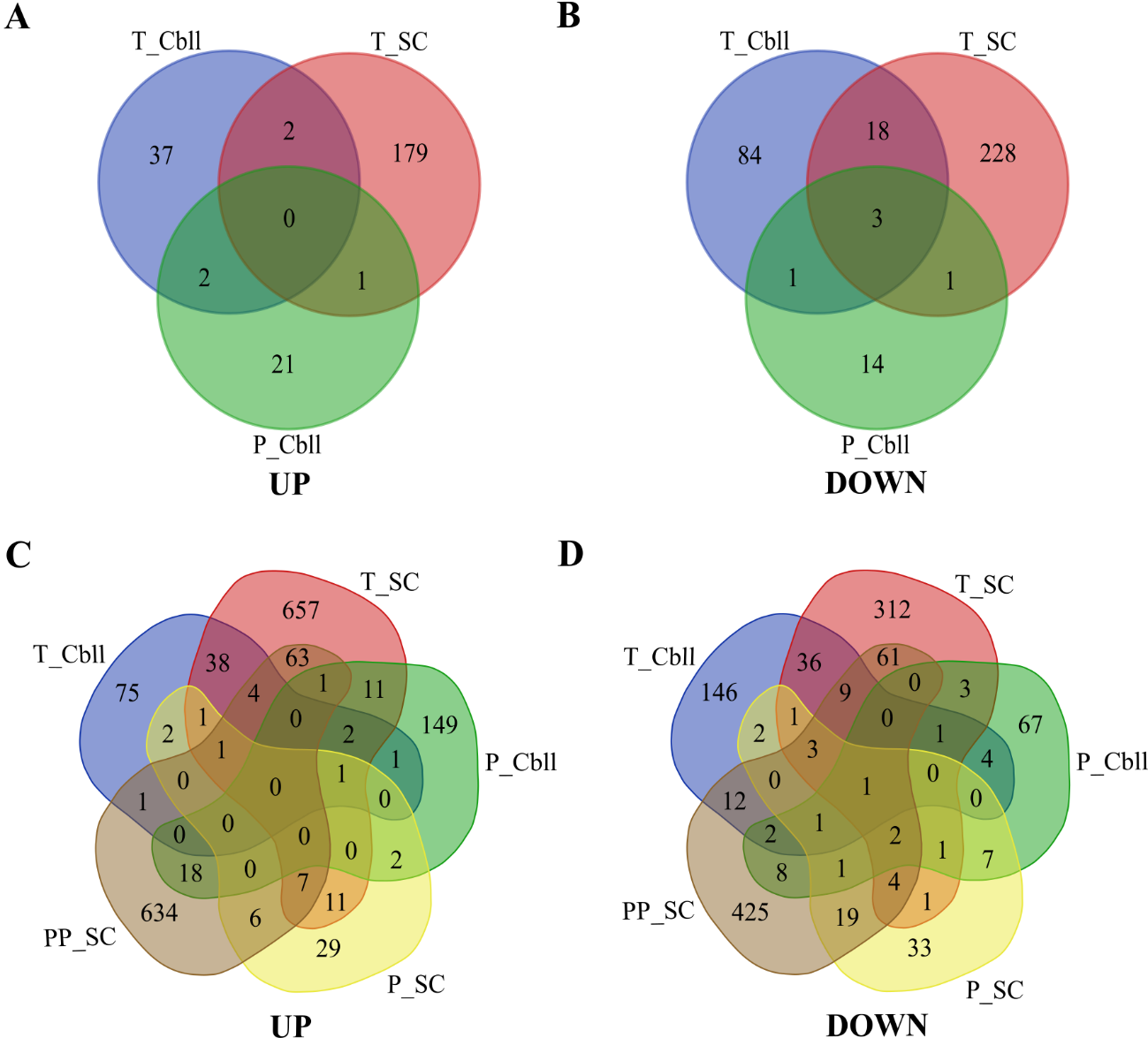


**Figure S7**


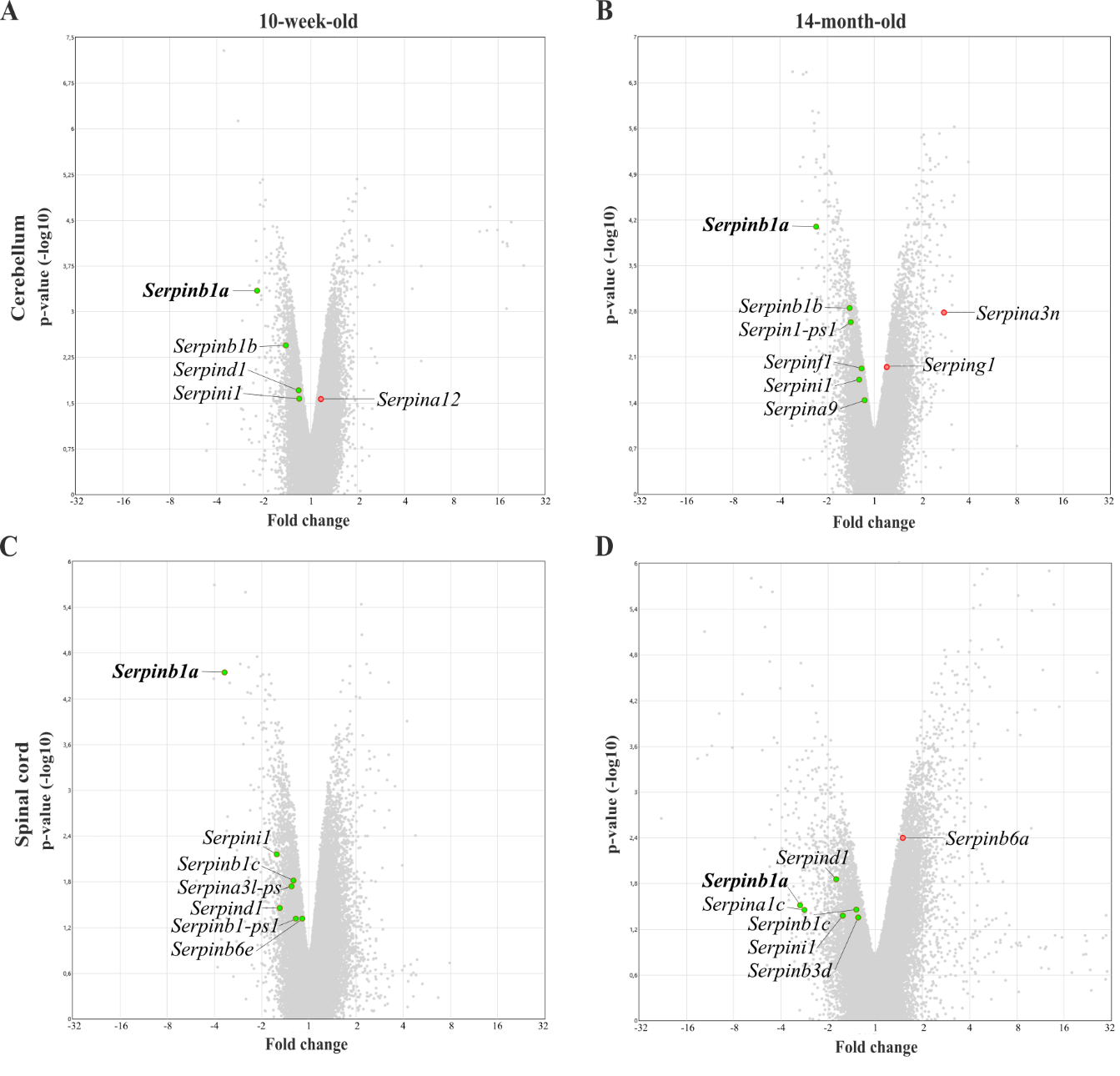


**Figure S8**


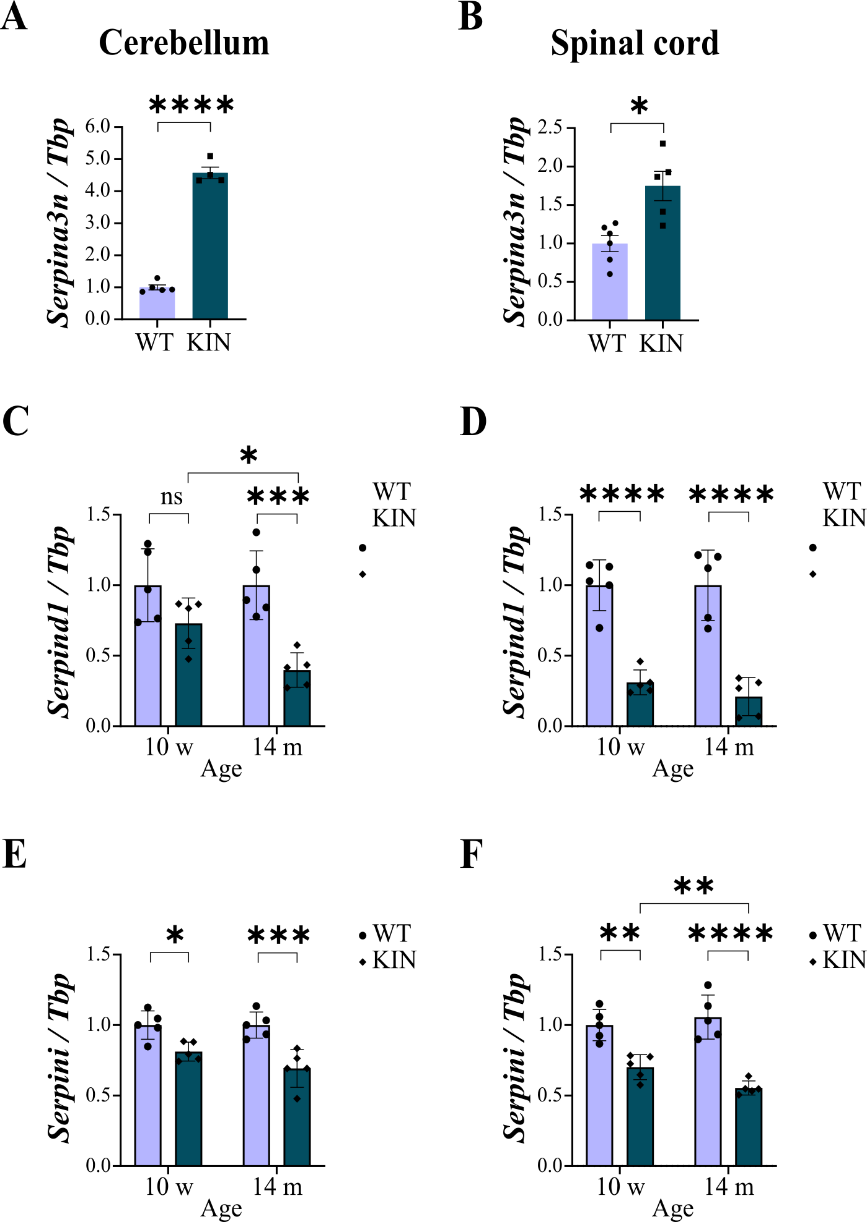


**Figure S9**


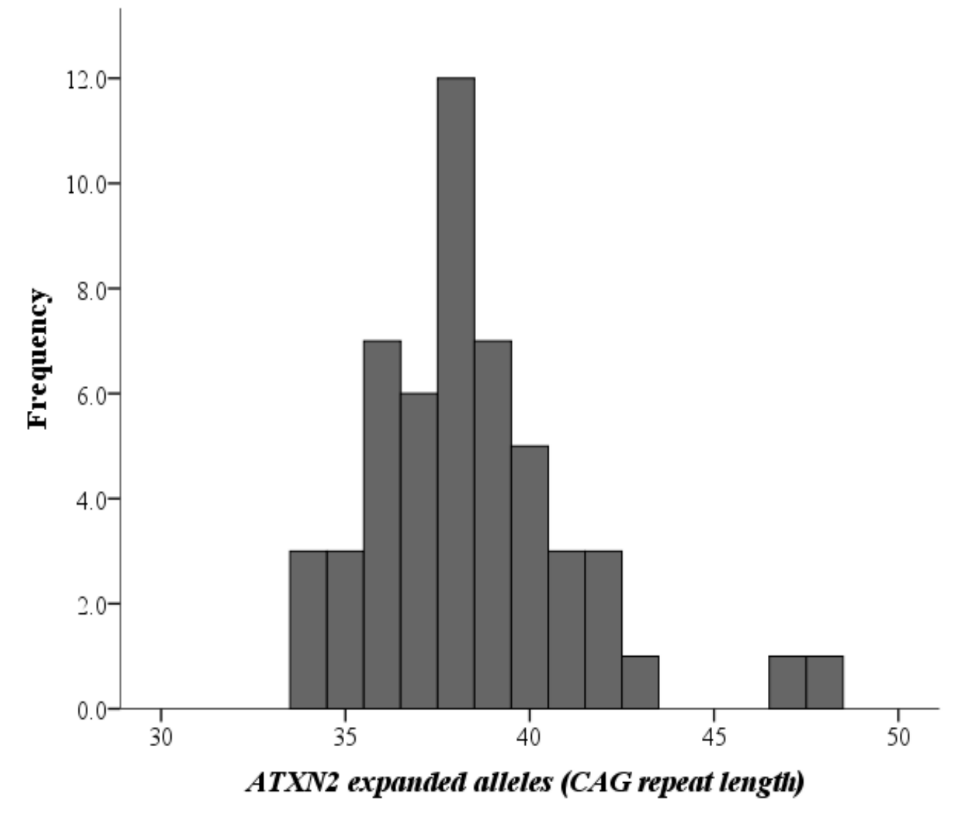


**Figure S10**


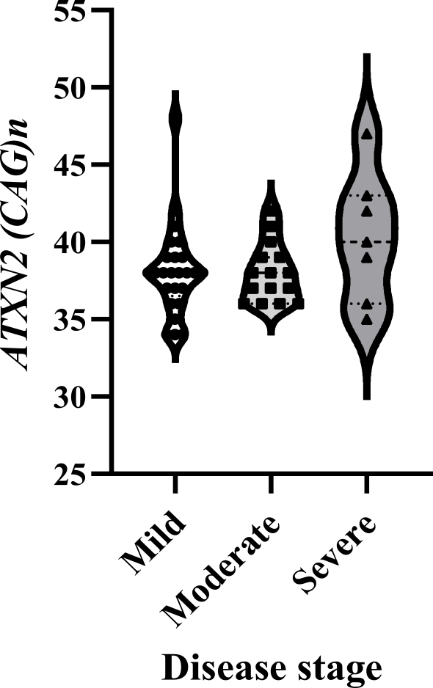


**Figure S11**


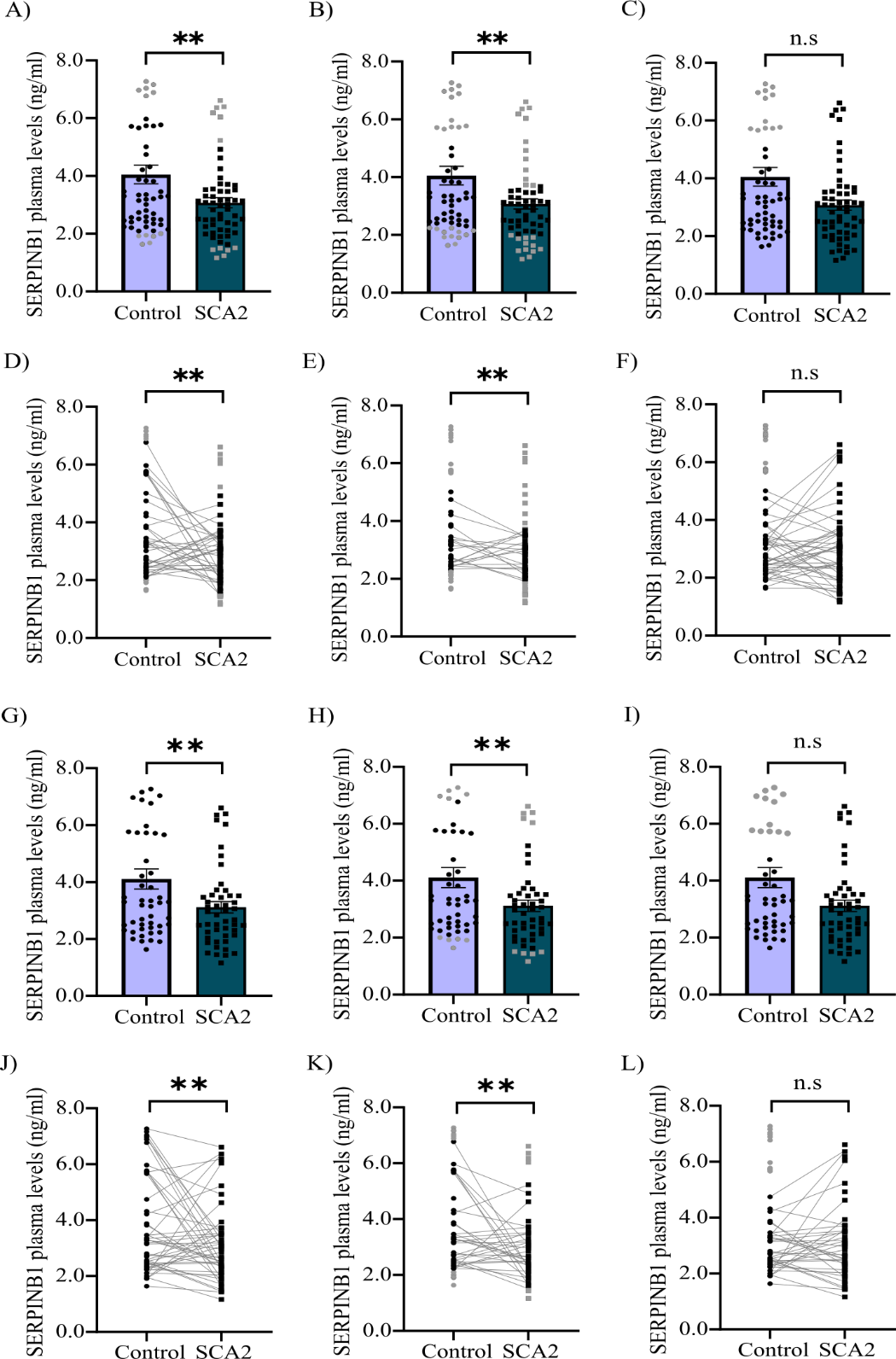


**Figure S12**


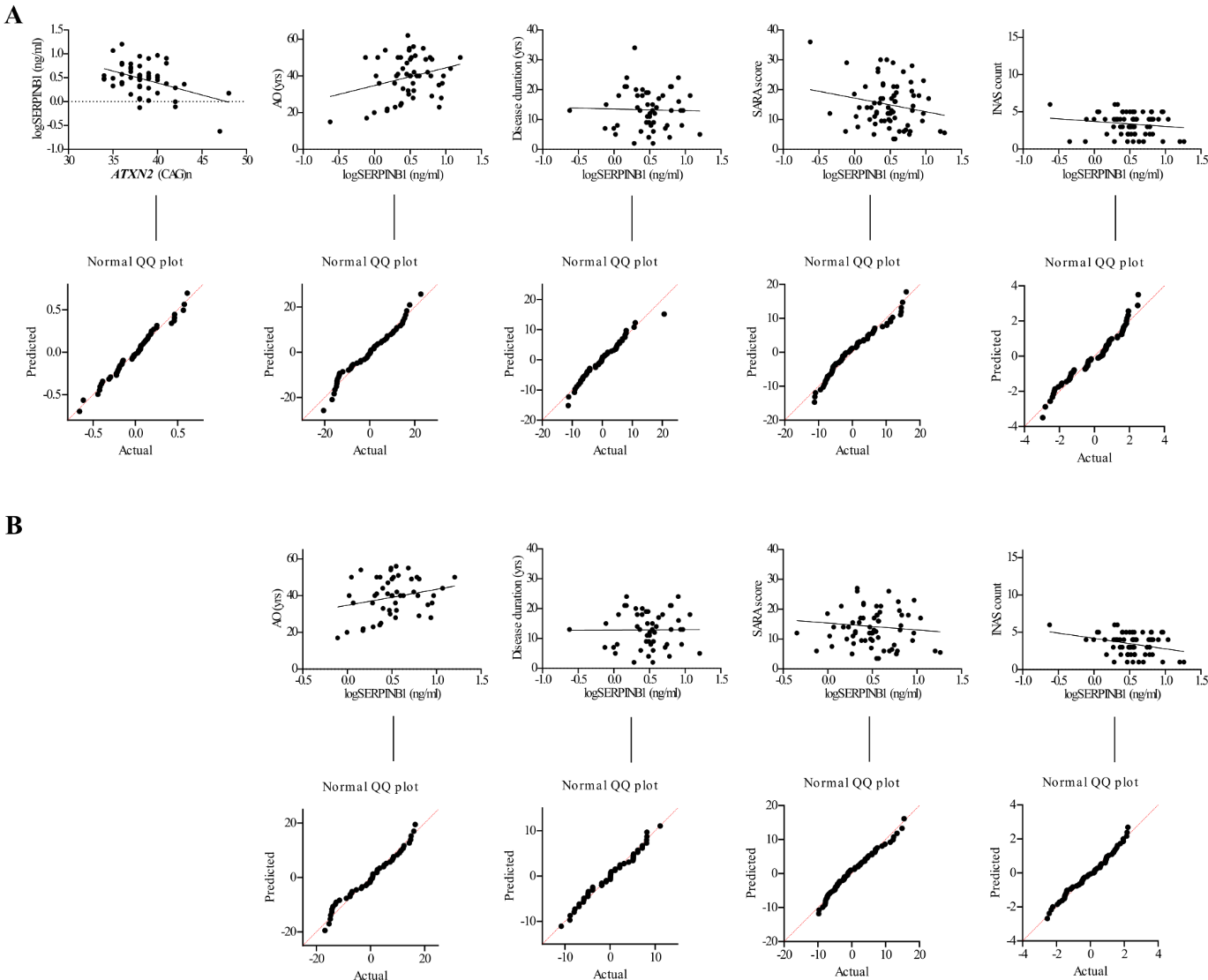


**Figure S13**


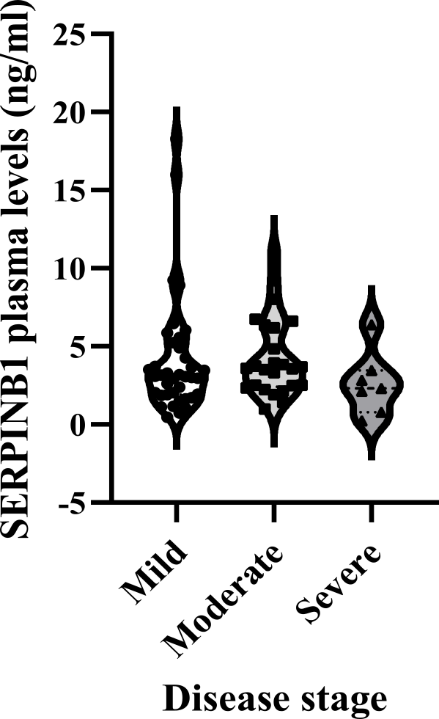


**Figure S14**

|  | **Cerebellum** |  |
| --- | --- | --- |
|  | SERPINB1A | ACTB |
| 10-week-old | 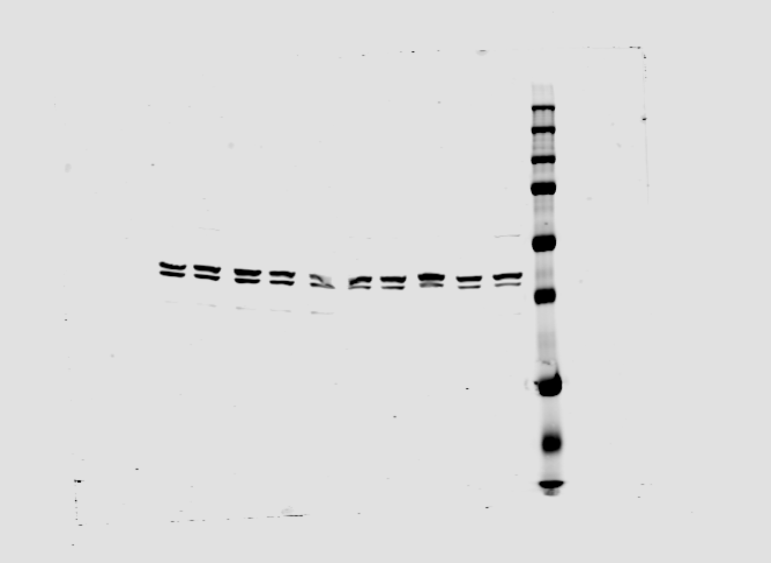 | 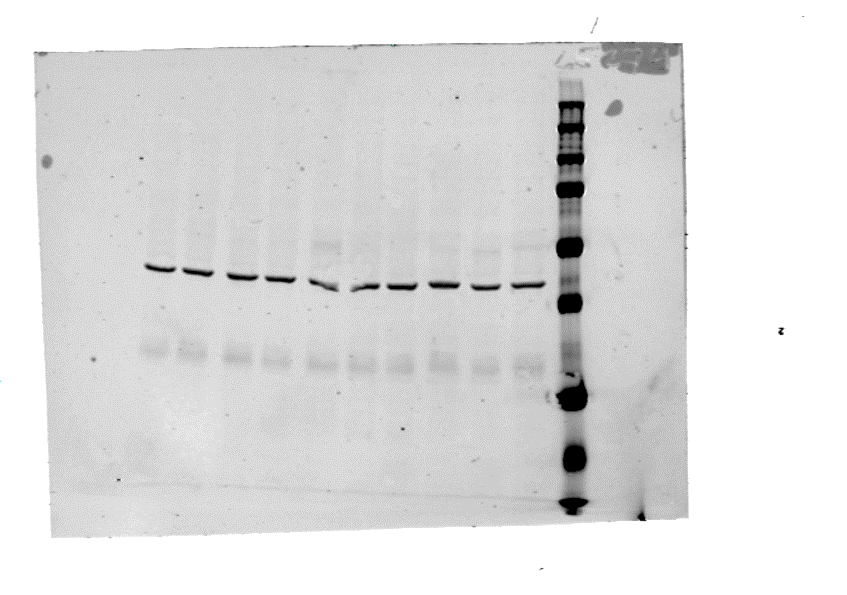 |
|  |  |  |
|  | SERPINB1A | ACTB |
| 6-month-old | 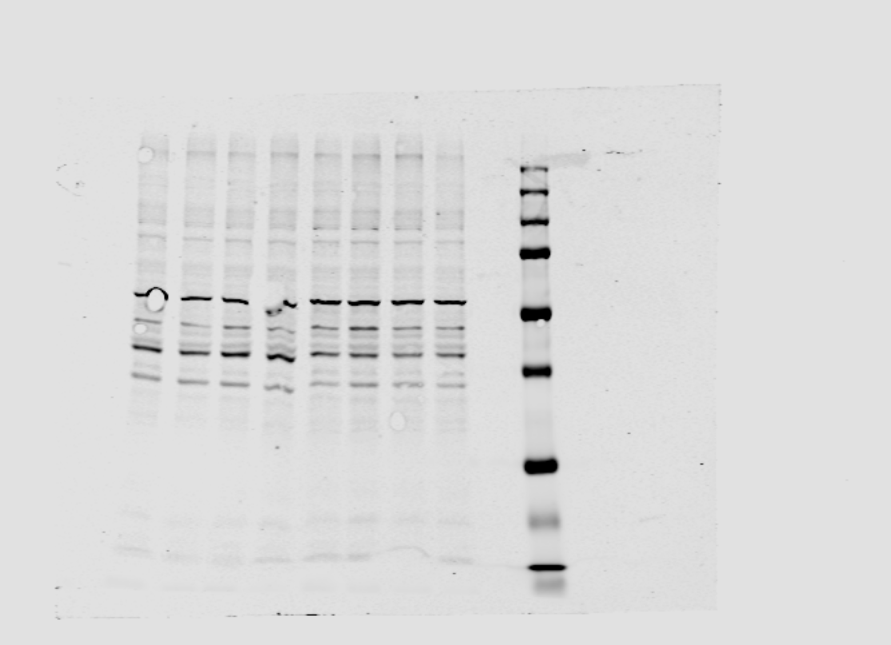 | 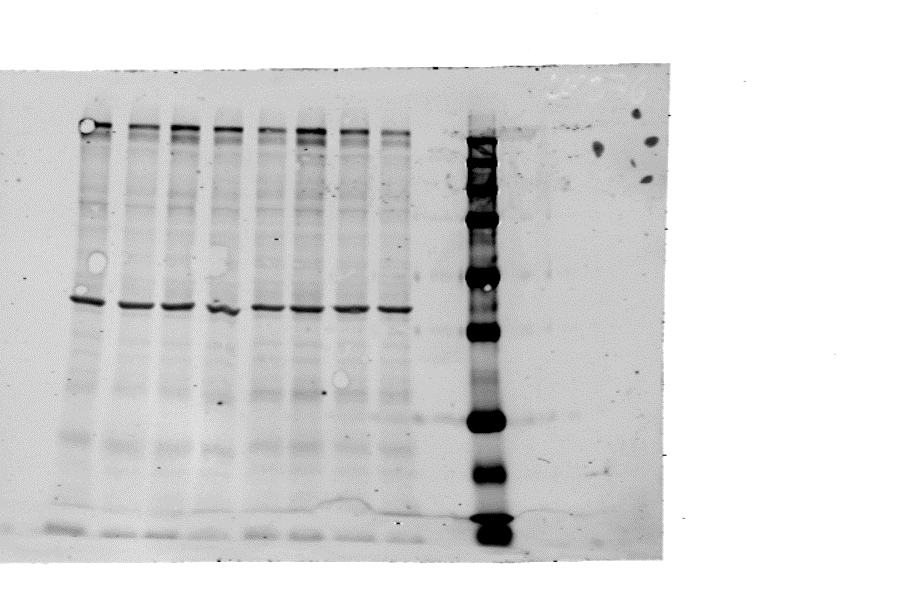 |
|  |  |  |
|  | SERPINB1A | ACTB |
| 14-month-old | 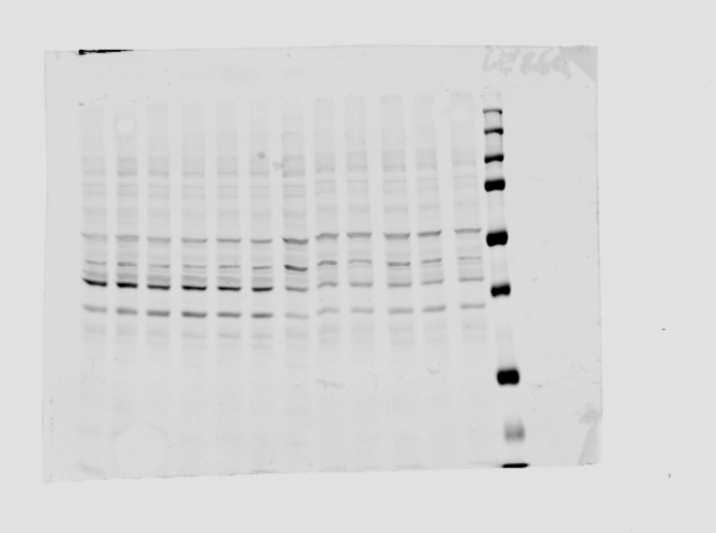 | 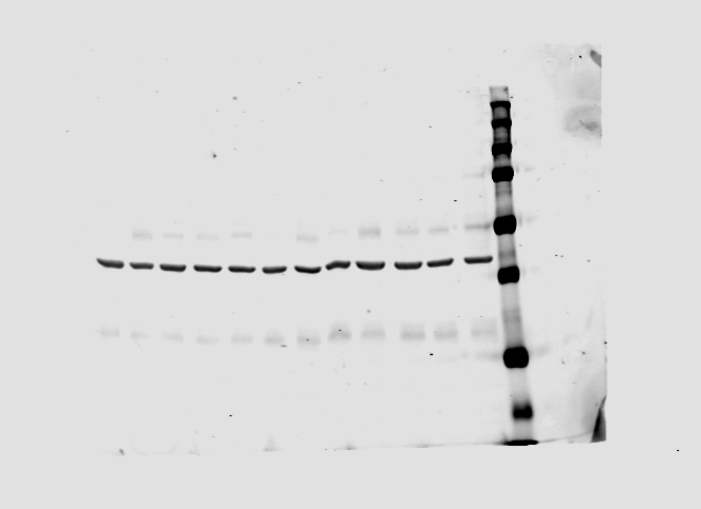 |

|  | **Spinal cord** |  |
| --- | --- | --- |
|  | SERPINB1A | ACTB |
| 10-week-old | 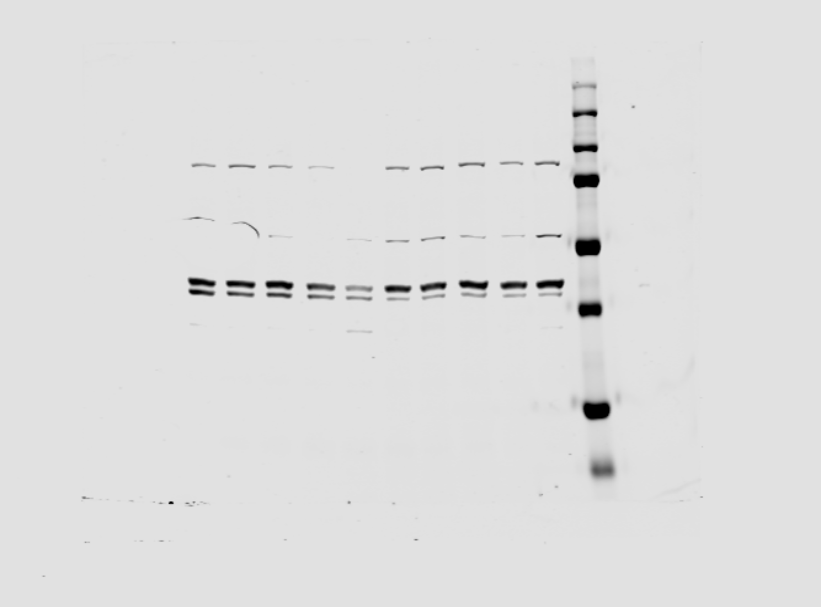 | 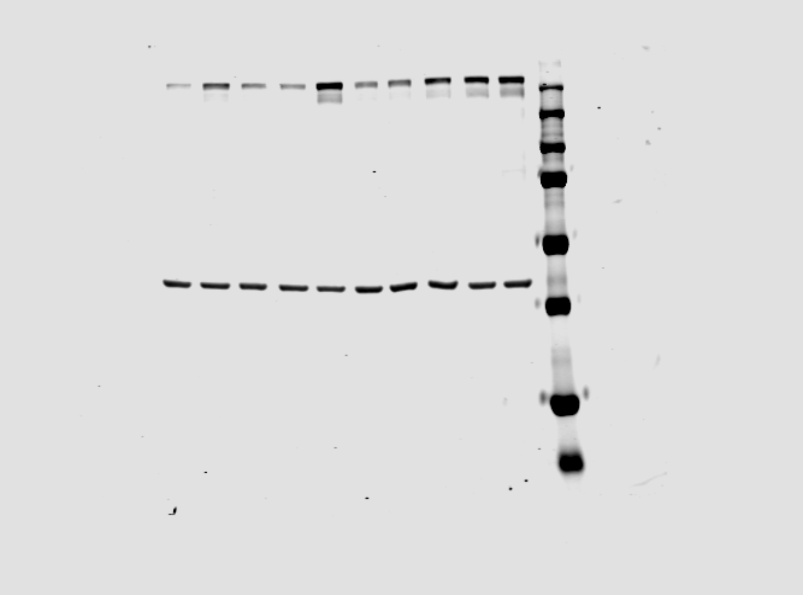 |
|  |  |  |
|  | SERPINB1A | ACTB |
| 6-month-old | 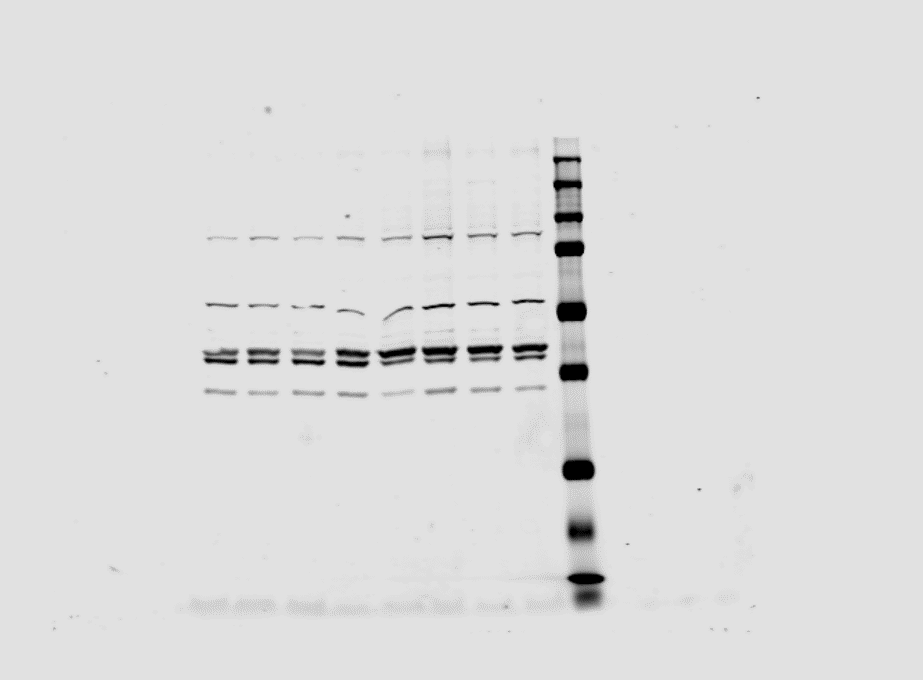 | 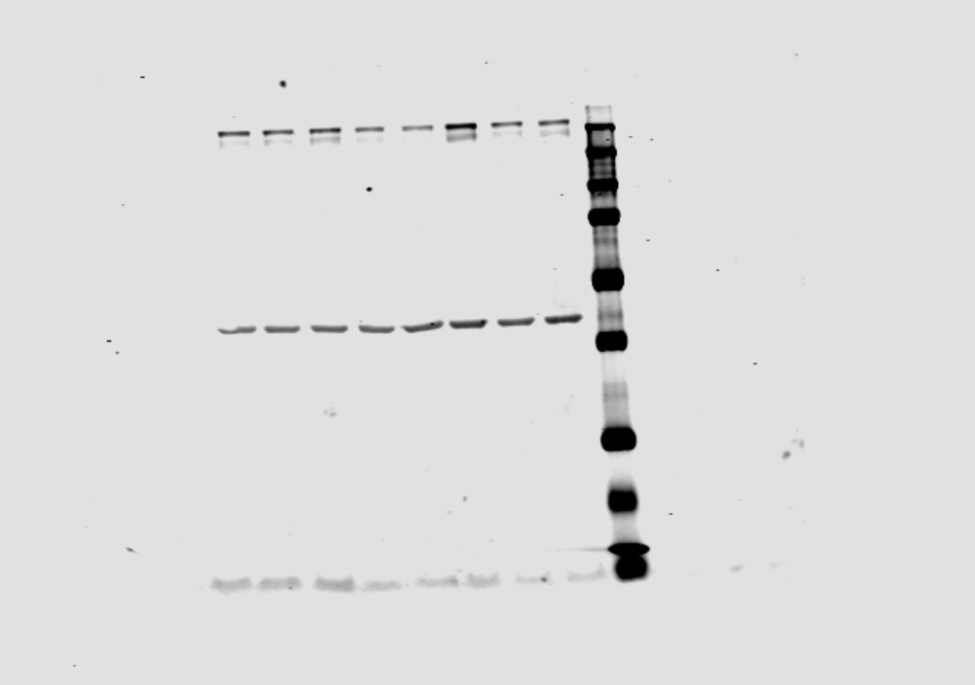 |
|  |  |  |
|  | SERPINB1A | ACTB |
| 14-month-old | 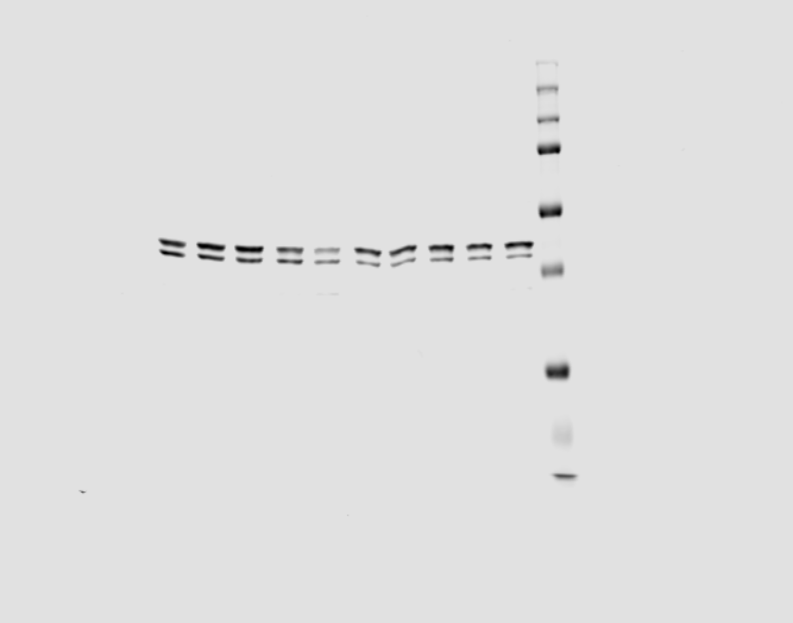 | 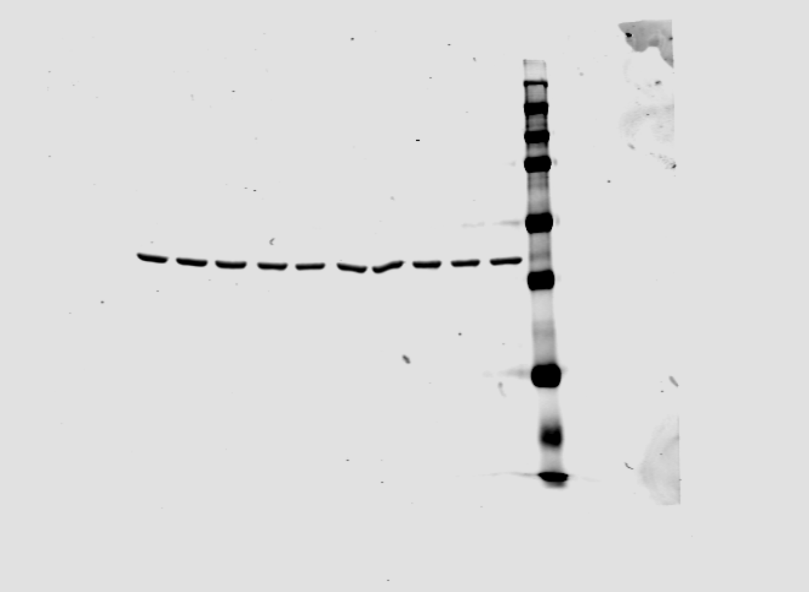 |

**Figure S15**

|  | **Cerebellum** |
| --- | --- |
|  | KLK6/GAPDH |
| 14-month-old | 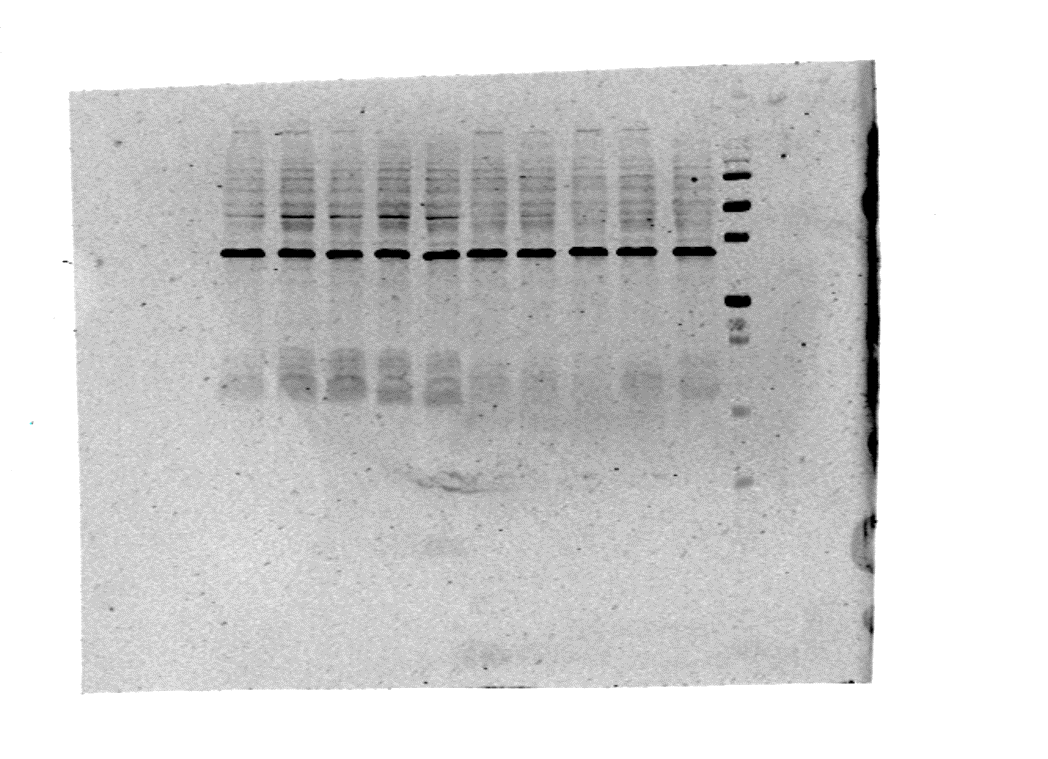 |
|  | **Spinal cord**  KLK6/GAPDH |
| 14-month-old | 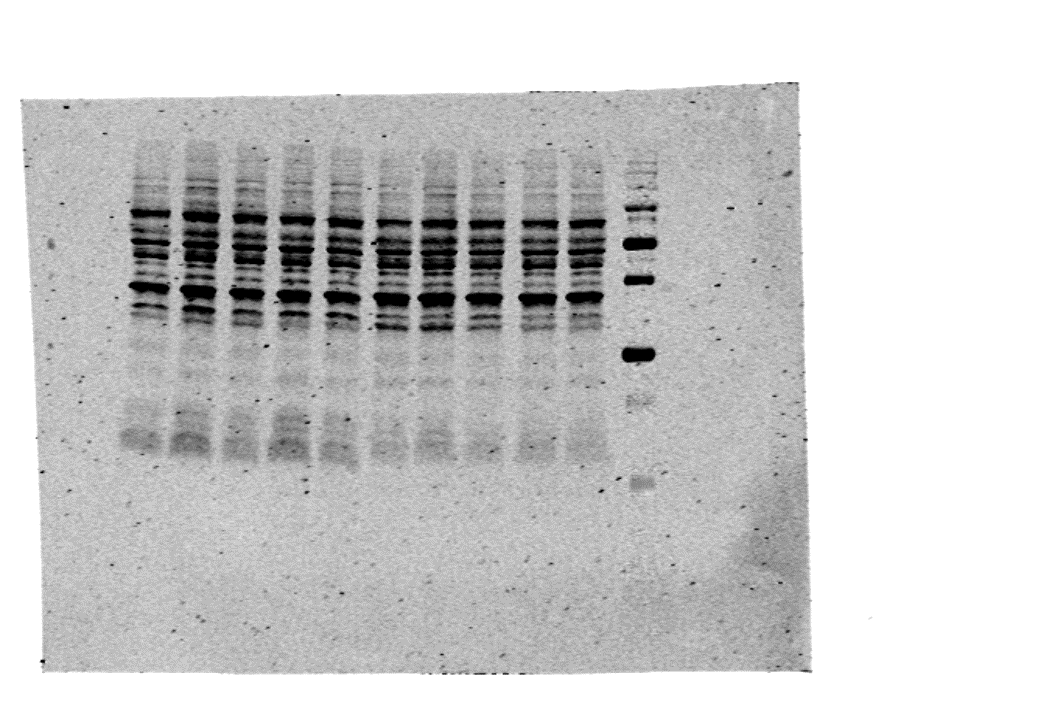 |

**Table S8**

Clinical and molecular characteristics of the
sex- and age-matched SCA2 patients and control individuals.

| Variables | SCA2 patients (N=58) | Controls (N=58) | χ^2^/t-test (p-value) |
| --- | --- | --- | --- |
|  | Mean (SD) | Mean (SD) |  |
| Sex (M/F) | 25/33 | 19/39 | 1.318 (0.251) |
| Age (years) | 51.12 (13.09) | 49.48 (12.53) | -0.688 (0.493) |
| SERPINB1 (ng/ml) | 3.07 (1.342) | 4.05 (2.459) | 2.618 (0.010) |
| Age at Onset (yrs) | 38.97 (11.31) | - | - |
| Disease Duration (yrs) | 12.89 (5.880) | - | - |
| SARA score | 16.08 (8.214) | - | - |
| INAS count | 3.34 (1.428) | - | - |
| *ATXN2* expanded alleles (CAG repeat length) | 38.6 (3.031) | - | - |

**Table S9**

**Receiver‑operator characteristic curve analysis for SERPINB1 plasma levels in patients with SCA2 versus control individuals**

| **Area under the ROC curve** | **58 vs 58** | **49 vs 49** |
| --- | --- | --- |
| Area | 0.6098 | 0.6183 |
| Std. Error | 0.05215 | 0.05639 |
| 95% confidence interval | 0.4837 to 0.6934 | 0.4892 to 0.7149 |
| P value | 0.0413 | 0.0436 |


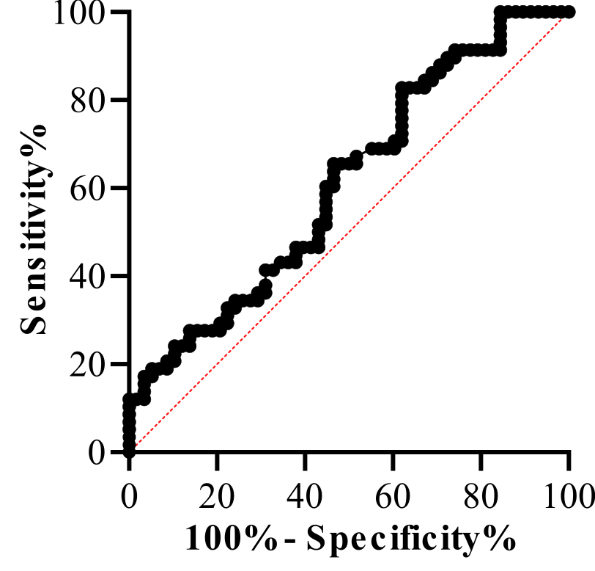


**Sensitivity and specificity analysis from the dataset of 58 vs 58 individuals**

| **Cut-off values** | **Sensitivity (%)** | **95% CI** | **Specificity (%)** | **95% CI** |
| --- | --- | --- | --- | --- |
| < 1.201 | 1.724 | 0.08844% to 9.141% | 100.0 | 93.79% to 100.0% |
| < 1.333 | 3.448 | 0.6127% to 11.73% | 100.0 | 93.79% to 100.0% |
| < 1.439 | 5.172 | 1.410% to 14.14% | 100.0 | 93.79% to 100.0% |
| < 1.473 | 6.897 | 2.714% to 16.43% | 100.0 | 93.79% to 100.0% |
| < 1.502 | 8.621 | 3.739% to 18.64% | 100.0 | 93.79% to 100.0% |
| < 1.571 | 10.34 | 4.828% to 20.79% | 100.0 | 93.79% to 100.0% |
| < 1.635 | 12.07 | 5.971% to 22.88% | 100.0 | 93.79% to 100.0% |
| < 1.661 | 12.07 | 5.971% to 22.88% | 98.28 | 90.86% to 99.91% |
| < 1.698 | 12.07 | 5.971% to 22.88% | 96.55 | 88.27% to 99.39% |
| < 1.791 | 13.79 | 7.158% to 24.93% | 96.55 | 88.27% to 99.39% |
| < 1.887 | 15.52 | 8.384% to 26.93% | 96.55 | 88.27% to 99.39% |
| < 1.908 | 17.24 | 9.644% to 28.91% | 96.55 | 88.27% to 99.39% |
| < 1.916 | 17.24 | 9.644% to 28.91% | 94.83 | 85.86% to 98.59% |
| < 1.921 | 18.97 | 10.93% to 30.85% | 94.83 | 85.86% to 98.59% |
| < 1.937 | 18.97 | 10.93% to 30.85% | 93.10 | 83.57% to 97.29% |
| < 1.970 | 18.97 | 10.93% to 30.85% | 91.38 | 81.36% to 96.26% |
| < 1.997 | 20.69 | 12.25% to 32.77% | 91.38 | 81.36% to 96.26% |
| < 2.018 | 20.69 | 12.25% to 32.77% | 89.66 | 79.21% to 95.17% |
| < 2.035 | 22.41 | 13.59% to 34.66% | 89.66 | 79.21% to 95.17% |
| < 2.066 | 24.14 | 14.96% to 36.53% | 89.66 | 79.21% to 95.17% |
| < 2.098 | 24.14 | 14.96% to 36.53% | 87.93 | 77.12% to 94.03% |
| < 2.108 | 24.14 | 14.96% to 36.53% | 86.21 | 75.07% to 92.84% |
| < 2.125 | 25.86 | 16.35% to 38.38% | 86.21 | 75.07% to 92.84% |
| < 2.142 | 27.59 | 17.75% to 40.20% | 86.21 | 75.07% to 92.84% |
| < 2.179 | 27.59 | 17.75% to 40.20% | 84.48 | 73.07% to 91.62% |
| < 2.221 | 27.59 | 17.75% to 40.20% | 82.76 | 71.09% to 90.36% |
| < 2.239 | 27.59 | 17.75% to 40.20% | 81.03 | 69.15% to 89.07% |
| < 2.246 | 27.59 | 17.75% to 40.20% | 79.31 | 67.23% to 87.75% |
| < 2.278 | 29.31 | 19.18% to 42.01% | 79.31 | 67.23% to 87.75% |
| < 2.309 | 29.31 | 19.18% to 42.01% | 77.59 | 65.34% to 86.41% |
| < 2.320 | 31.03 | 20.62% to 43.80% | 77.59 | 65.34% to 86.41% |
| < 2.340 | 32.76 | 22.08% to 45.58% | 77.59 | 65.34% to 86.41% |
| < 2.367 | 32.76 | 22.08% to 45.58% | 75.86 | 63.47% to 85.04% |
| < 2.393 | 34.48 | 23.56% to 47.33% | 75.86 | 63.47% to 85.04% |
| < 2.412 | 34.48 | 23.56% to 47.33% | 74.14 | 61.62% to 83.65% |
| < 2.439 | 34.48 | 23.56% to 47.33% | 72.41 | 59.80% to 82.25% |
| < 2.470 | 34.48 | 23.56% to 47.33% | 70.69 | 57.99% to 80.82% |
| < 2.485 | 36.21 | 25.05% to 49.07% | 70.69 | 57.99% to 80.82% |
| < 2.490 | 36.21 | 25.05% to 49.07% | 68.97 | 56.20% to 79.38% |
| < 2.501 | 37.93 | 26.56% to 50.80% | 68.97 | 56.20% to 79.38% |
| < 2.511 | 41.38 | 29.63% to 54.20% | 68.97 | 56.20% to 79.38% |
| < 2.514 | 41.38 | 29.63% to 54.20% | 67.24 | 54.42% to 77.92% |
| < 2.538 | 43.10 | 31.18% to 55.88% | 65.52 | 52.67% to 76.44% |
| < 2.576 | 43.10 | 31.18% to 55.88% | 63.79 | 50.93% to 74.95% |
| < 2.607 | 43.10 | 31.18% to 55.88% | 62.07 | 49.20% to 73.44% |
| < 2.651 | 44.83 | 32.75% to 57.55% | 62.07 | 49.20% to 73.44% |
| < 2.688 | 46.55 | 34.33% to 59.20% | 62.07 | 49.20% to 73.44% |
| < 2.717 | 46.55 | 34.33% to 59.20% | 60.34 | 47.49% to 71.91% |
| < 2.770 | 46.55 | 34.33% to 59.20% | 58.62 | 45.80% to 70.37% |
| < 2.817 | 46.55 | 34.33% to 59.20% | 56.90 | 44.12% to 68.82% |
| < 2.841 | 48.28 | 35.93% to 60.84% | 56.90 | 44.12% to 68.82% |
| < 2.906 | 50.00 | 37.54% to 62.46% | 56.90 | 44.12% to 68.82% |
| < 2.988 | 51.72 | 39.16% to 64.07% | 56.90 | 44.12% to 68.82% |
| < 3.016 | 51.72 | 39.16% to 64.07% | 55.17 | 42.45% to 67.25% |
| < 3.043 | 53.45 | 40.80% to 65.67% | 55.17 | 42.45% to 67.25% |
| < 3.075 | 55.17 | 42.45% to 67.25% | 55.17 | 42.45% to 67.25% |
| < 3.107 | 56.90 | 44.12% to 68.82% | 55.17 | 42.45% to 67.25% |
| < 3.133 | 58.62 | 45.80% to 70.37% | 55.17 | 42.45% to 67.25% |
| < 3.144 | 60.34 | 47.49% to 71.91% | 55.17 | 42.45% to 67.25% |
| < 3.157 | 60.34 | 47.49% to 71.91% | 53.45 | 40.80% to 65.67% |
| < 3.162 | 62.07 | 49.20% to 73.44% | 53.45 | 40.80% to 65.67% |
| < 3.175 | 63.79 | 50.93% to 74.95% | 53.45 | 40.80% to 65.67% |
| < 3.187 | 65.52 | 52.67% to 76.44% | 53.45 | 40.80% to 65.67% |
| < 3.200 | 65.52 | 52.67% to 76.44% | 51.72 | 39.16% to 64.07% |
| < 3.244 | 65.52 | 52.67% to 76.44% | 50.00 | 37.54% to 62.46% |
| < 3.281 | 65.52 | 52.67% to 76.44% | 48.28 | 35.93% to 60.84% |
| < 3.296 | 67.24 | 54.42% to 77.92% | 48.28 | 35.93% to 60.84% |
| < 3.324 | 68.97 | 56.20% to 79.38% | 44.83 | 32.75% to 57.55% |
| < 3.350 | 68.97 | 56.20% to 79.38% | 43.10 | 31.18% to 55.88% |
| < 3.407 | 68.97 | 56.20% to 79.38% | 41.38 | 29.63% to 54.20% |
| < 3.459 | 68.97 | 56.20% to 79.38% | 39.66 | 28.09% to 52.51% |
| < 3.464 | 70.69 | 57.99% to 80.82% | 39.66 | 28.09% to 52.51% |
| < 3.469 | 70.69 | 57.99% to 80.82% | 37.93 | 26.56% to 50.80% |
| < 3.495 | 72.41 | 59.80% to 82.25% | 37.93 | 26.56% to 50.80% |
| < 3.521 | 74.14 | 61.62% to 83.65% | 37.93 | 26.56% to 50.80% |
| < 3.538 | 75.86 | 63.47% to 85.04% | 37.93 | 26.56% to 50.80% |
| < 3.614 | 77.59 | 65.34% to 86.41% | 37.93 | 26.56% to 50.80% |
| < 3.692 | 79.31 | 67.23% to 87.75% | 37.93 | 26.56% to 50.80% |
| < 3.721 | 81.03 | 69.15% to 89.07% | 37.93 | 26.56% to 50.80% |
| < 3.776 | 82.76 | 71.09% to 90.36% | 37.93 | 26.56% to 50.80% |
| < 3.829 | 82.76 | 71.09% to 90.36% | 36.21 | 25.05% to 49.07% |
| < 3.858 | 82.76 | 71.09% to 90.36% | 34.48 | 23.56% to 47.33% |
| < 3.904 | 82.76 | 71.09% to 90.36% | 32.76 | 22.08% to 45.58% |
| < 4.074 | 84.48 | 73.07% to 91.62% | 32.76 | 22.08% to 45.58% |
| < 4.233 | 84.48 | 73.07% to 91.62% | 31.03 | 20.62% to 43.80% |
| < 4.282 | 86.21 | 75.07% to 92.84% | 31.03 | 20.62% to 43.80% |
| < 4.470 | 86.21 | 75.07% to 92.84% | 29.31 | 19.18% to 42.01% |
| < 4.686 | 87.93 | 77.12% to 94.03% | 29.31 | 19.18% to 42.01% |
| < 4.835 | 87.93 | 77.12% to 94.03% | 27.59 | 17.75% to 40.20% |
| < 4.968 | 89.66 | 79.21% to 95.17% | 27.59 | 17.75% to 40.20% |
| < 5.123 | 89.66 | 79.21% to 95.17% | 25.86 | 16.35% to 38.38% |
| < 5.449 | 91.38 | 81.36% to 96.26% | 25.86 | 16.35% to 38.38% |
| < 5.691 | 91.38 | 81.36% to 96.26% | 24.14 | 14.96% to 36.53% |
| < 5.722 | 91.38 | 81.36% to 96.26% | 22.41 | 13.59% to 34.66% |
| < 5.732 | 91.38 | 81.36% to 96.26% | 20.69 | 12.25% to 32.77% |
| < 5.752 | 91.38 | 81.36% to 96.26% | 18.97 | 10.93% to 30.85% |
| < 5.870 | 91.38 | 81.36% to 96.26% | 17.24 | 9.644% to 28.91% |
| < 6.004 | 91.38 | 81.36% to 96.26% | 15.52 | 8.384% to 26.93% |
| < 6.109 | 93.10 | 83.57% to 97.29% | 15.52 | 8.384% to 26.93% |
| < 6.274 | 94.83 | 85.86% to 98.59% | 15.52 | 8.384% to 26.93% |
| < 6.383 | 96.55 | 88.27% to 99.39% | 15.52 | 8.384% to 26.93% |
| < 6.504 | 98.28 | 90.86% to 99.91% | 15.52 | 8.384% to 26.93% |
| < 6.688 | 100.0 | 93.79% to 100.0% | 15.52 | 8.384% to 26.93% |
| < 6.829 | 100.0 | 93.79% to 100.0% | 13.79 | 7.158% to 24.93% |
| < 6.932 | 100.0 | 93.79% to 100.0% | 12.07 | 5.971% to 22.88% |
| < 7.005 | 100.0 | 93.79% to 100.0% | 10.34 | 4.828% to 20.79% |
| < 7.099 | 100.0 | 93.79% to 100.0% | 8.621 | 3.739% to 18.64% |
| < 7.273 | 100.0 | 93.79% to 100.0% | 6.897 | 2.714% to 16.43% |

**Sensitivity and specificity analysis from the dataset of 49 vs 49 individuals**

| **Cut-off values** | **Sensitivity (%)** | **95% CI** | **Specificity (%)** | **95% CI** |
| --- | --- | --- | --- | --- |
| < 1.295 | 2.041 | 0.1047% to 10.69% | 100.0 | 92.73% to 100.0% |
| < 1.439 | 4.082 | 0.7252% to 13.71% | 100.0 | 92.73% to 100.0% |
| < 1.473 | 6.122 | 2.104% to 16.52% | 100.0 | 92.73% to 100.0% |
| < 1.502 | 8.163 | 3.220% to 19.19% | 100.0 | 92.73% to 100.0% |
| < 1.571 | 10.20 | 4.438% to 21.76% | 100.0 | 92.73% to 100.0% |
| < 1.635 | 12.24 | 5.735% to 24.24% | 100.0 | 92.73% to 100.0% |
| < 1.674 | 12.24 | 5.735% to 24.24% | 97.96 | 89.31% to 99.90% |
| < 1.791 | 14.29 | 7.096% to 26.67% | 97.96 | 89.31% to 99.90% |
| < 1.887 | 16.33 | 8.513% to 29.04% | 97.96 | 89.31% to 99.90% |
| < 1.908 | 18.37 | 9.976% to 31.36% | 97.96 | 89.31% to 99.90% |
| < 1.916 | 18.37 | 9.976% to 31.36% | 95.92 | 86.29% to 99.27% |
| < 1.921 | 20.41 | 11.48% to 33.64% | 95.92 | 86.29% to 99.27% |
| < 1.937 | 20.41 | 11.48% to 33.64% | 93.88 | 83.48% to 97.90% |
| < 1.977 | 20.41 | 11.48% to 33.64% | 91.84 | 80.81% to 96.78% |
| < 2.020 | 20.41 | 11.48% to 33.64% | 89.80 | 78.24% to 95.56% |
| < 2.069 | 22.45 | 13.02% to 35.88% | 89.80 | 78.24% to 95.56% |
| < 2.108 | 22.45 | 13.02% to 35.88% | 87.76 | 75.76% to 94.27% |
| < 2.125 | 24.49 | 14.60% to 38.09% | 87.76 | 75.76% to 94.27% |
| < 2.172 | 26.53 | 16.21% to 40.26% | 87.76 | 75.76% to 94.27% |
| < 2.221 | 26.53 | 16.21% to 40.26% | 85.71 | 73.33% to 92.90% |
| < 2.239 | 26.53 | 16.21% to 40.26% | 83.67 | 70.96% to 91.49% |
| < 2.246 | 26.53 | 16.21% to 40.26% | 81.63 | 68.64% to 90.02% |
| < 2.278 | 28.57 | 17.85% to 42.41% | 81.63 | 68.64% to 90.02% |
| < 2.309 | 28.57 | 17.85% to 42.41% | 79.59 | 66.36% to 88.52% |
| < 2.320 | 30.61 | 19.52% to 44.53% | 79.59 | 66.36% to 88.52% |
| < 2.340 | 32.65 | 21.21% to 46.62% | 79.59 | 66.36% to 88.52% |
| < 2.367 | 32.65 | 21.21% to 46.62% | 77.55 | 64.12% to 86.98% |
| < 2.393 | 34.69 | 22.92% to 48.69% | 77.55 | 64.12% to 86.98% |
| < 2.430 | 34.69 | 22.92% to 48.69% | 75.51 | 61.91% to 85.40% |
| < 2.470 | 34.69 | 22.92% to 48.69% | 73.47 | 59.74% to 83.79% |
| < 2.485 | 36.73 | 24.67% to 50.73% | 73.47 | 59.74% to 83.79% |
| < 2.490 | 36.73 | 24.67% to 50.73% | 71.43 | 57.59% to 82.15% |
| < 2.501 | 38.78 | 26.43% to 52.75% | 71.43 | 57.59% to 82.15% |
| < 2.511 | 42.86 | 30.02% to 56.73% | 71.43 | 57.59% to 82.15% |
| < 2.514 | 42.86 | 30.02% to 56.73% | 69.39 | 55.47% to 80.48% |
| < 2.538 | 44.90 | 31.85% to 58.68% | 67.35 | 53.38% to 78.79% |
| < 2.576 | 44.90 | 31.85% to 58.68% | 65.31 | 51.31% to 77.08% |
| < 2.607 | 44.90 | 31.85% to 58.68% | 63.27 | 49.27% to 75.33% |
| < 2.651 | 46.94 | 33.70% to 60.62% | 63.27 | 49.27% to 75.33% |
| < 2.688 | 48.98 | 35.58% to 62.53% | 63.27 | 49.27% to 75.33% |
| < 2.717 | 48.98 | 35.58% to 62.53% | 61.22 | 47.25% to 73.57% |
| < 2.770 | 48.98 | 35.58% to 62.53% | 59.18 | 45.25% to 71.78% |
| < 2.826 | 48.98 | 35.58% to 62.53% | 57.14 | 43.27% to 69.98% |
| < 2.906 | 51.02 | 37.47% to 64.42% | 57.14 | 43.27% to 69.98% |
| < 2.990 | 53.06 | 39.38% to 66.30% | 57.14 | 43.27% to 69.98% |
| < 3.043 | 55.10 | 41.32% to 68.15% | 57.14 | 43.27% to 69.98% |
| < 3.099 | 57.14 | 43.27% to 69.98% | 57.14 | 43.27% to 69.98% |
| < 3.142 | 59.18 | 45.25% to 71.78% | 57.14 | 43.27% to 69.98% |
| < 3.159 | 59.18 | 45.25% to 71.78% | 55.10 | 41.32% to 68.15% |
| < 3.175 | 61.22 | 47.25% to 73.57% | 55.10 | 41.32% to 68.15% |
| < 3.187 | 63.27 | 49.27% to 75.33% | 55.10 | 41.32% to 68.15% |
| < 3.231 | 63.27 | 49.27% to 75.33% | 53.06 | 39.38% to 66.30% |
| < 3.281 | 63.27 | 49.27% to 75.33% | 51.02 | 37.47% to 64.42% |
| < 3.296 | 65.31 | 51.31% to 77.08% | 51.02 | 37.47% to 64.42% |
| < 3.324 | 67.35 | 53.38% to 78.79% | 46.94 | 33.70% to 60.62% |
| < 3.350 | 67.35 | 53.38% to 78.79% | 44.90 | 31.85% to 58.68% |
| < 3.407 | 67.35 | 53.38% to 78.79% | 42.86 | 30.02% to 56.73% |
| < 3.459 | 67.35 | 53.38% to 78.79% | 40.82 | 28.22% to 54.75% |
| < 3.464 | 69.39 | 55.47% to 80.48% | 40.82 | 28.22% to 54.75% |
| < 3.469 | 69.39 | 55.47% to 80.48% | 38.78 | 26.43% to 52.75% |
| < 3.495 | 71.43 | 57.59% to 82.15% | 38.78 | 26.43% to 52.75% |
| < 3.521 | 73.47 | 59.74% to 83.79% | 38.78 | 26.43% to 52.75% |
| < 3.538 | 75.51 | 61.91% to 85.40% | 38.78 | 26.43% to 52.75% |
| < 3.630 | 77.55 | 64.12% to 86.98% | 38.78 | 26.43% to 52.75% |
| < 3.721 | 79.59 | 66.36% to 88.52% | 38.78 | 26.43% to 52.75% |
| < 3.776 | 81.63 | 68.64% to 90.02% | 38.78 | 26.43% to 52.75% |
| < 3.847 | 81.63 | 68.64% to 90.02% | 36.73 | 24.67% to 50.73% |
| < 3.904 | 81.63 | 68.64% to 90.02% | 34.69 | 22.92% to 48.69% |
| < 4.074 | 83.67 | 70.96% to 91.49% | 34.69 | 22.92% to 48.69% |
| < 4.265 | 83.67 | 70.96% to 91.49% | 32.65 | 21.21% to 46.62% |
| < 4.470 | 83.67 | 70.96% to 91.49% | 30.61 | 19.52% to 44.53% |
| < 4.686 | 85.71 | 73.33% to 92.90% | 30.61 | 19.52% to 44.53% |
| < 4.835 | 85.71 | 73.33% to 92.90% | 28.57 | 17.85% to 42.41% |
| < 5.079 | 87.76 | 75.76% to 94.27% | 28.57 | 17.85% to 42.41% |
| < 5.449 | 89.80 | 78.24% to 95.56% | 28.57 | 17.85% to 42.41% |
| < 5.691 | 89.80 | 78.24% to 95.56% | 26.53 | 16.21% to 40.26% |
| < 5.722 | 89.80 | 78.24% to 95.56% | 24.49 | 14.60% to 38.09% |
| < 5.732 | 89.80 | 78.24% to 95.56% | 22.45 | 13.02% to 35.88% |
| < 5.752 | 89.80 | 78.24% to 95.56% | 20.41 | 11.48% to 33.64% |
| < 5.870 | 89.80 | 78.24% to 95.56% | 18.37 | 9.976% to 31.36% |
| < 6.004 | 89.80 | 78.24% to 95.56% | 16.33 | 8.513% to 29.04% |
| < 6.109 | 91.84 | 80.81% to 96.78% | 16.33 | 8.513% to 29.04% |
| < 6.274 | 93.88 | 83.48% to 97.90% | 16.33 | 8.513% to 29.04% |
| < 6.383 | 95.92 | 86.29% to 99.27% | 16.33 | 8.513% to 29.04% |
| < 6.504 | 97.96 | 89.31% to 99.90% | 16.33 | 8.513% to 29.04% |
| < 6.688 | 100.0 | 92.73% to 100.0% | 16.33 | 8.513% to 29.04% |
| < 6.829 | 100.0 | 92.73% to 100.0% | 14.29 | 7.096% to 26.67% |
| < 6.932 | 100.0 | 92.73% to 100.0% | 12.24 | 5.735% to 24.24% |
| < 7.005 | 100.0 | 92.73% to 100.0% | 10.20 | 4.438% to 21.76% |
| < 7.099 | 100.0 | 92.73% to 100.0% | 8.163 | 3.220% to 19.19% |
| < 7.217 | 100.0 | 92.73% to 100.0% | 6.122 | 2.104% to 16.52% |

**Linear regression analyses of the CAG repeat length of *ATXN2* expanded alleles and SERPINB1 plasma levels on the age at disease onset (AO)**

| Regression model | Dependent variable | Predictors | β (SE) | t (p) | R | R^2^ | R^2^_adj_ |
| --- | --- | --- | --- | --- | --- | --- | --- |
| 1 | AO | *ATXN2* expanded alleles | -0.203 (0.021) | -9.505 (<0.001) | 0.805 | 0.648 | 0.641 |
| 2 | AO | *ATXN2* expanded alleles | -0.189 (0.021) | -8.821 (<0.001) | 0.825 | 0.680 | 0.667 |
|  |  | SERPINB1 plasma levels | -1.553 (0.710) | -2.187 (0.034) |  |  |  |

These results from linear regression analyses indicated that 2.6% of the total variability in the age at onset might be attributable to the variations in SERPINB1 plasma levels. Thus, beyond the CAG expansion effect some 7.24% of the unexplained variation in age at onset might be reflected by variation at SERPINB1 plasma levels.

| **Covariance analysis for SERPINB1 in the whole dataset of cases and controls (58vs58)**  Covariance analyses were based on the age, sex, group, and highSERPINB1 as covariates. The CAG expansion was not included because it only happens in the patients group.  1-Baseline ANCOVA   \| **Tests of Between-Subjects Effects** \| \| \| \| \| \| \| \| \| \| \| \| \| --- \| --- \| --- \| --- \| --- \| --- \| --- \| --- \| --- \| --- \| --- \| --- \| \| Dependent Variable: serpinb1 \| \| \| \| \| \| \| \| \| \| \| \| \| Source \| \| Type III Sum of Squares \| \| df \| \| Mean Square \| \| \| F \| Sig. \| \| \| Corrected Model \| \| 37,851^a^ \| \| 3 \| \| 12,617 \| \| \| 2,989 \| ,034 \| \| \| Intercept \| \| 24,576 \| \| 1 \| \| 24,576 \| \| \| 5,822 \| ,017 \| \| \| **Group** \| \| 32,726 \| \| 1 \| \| 32,726 \| \| \| 7,752 \| **,006** \| \| \| **Age** \| \| 4,760 \| \| 1 \| \| 4,760 \| \| \| 1,128 \| ,291 \| \| \| **Sex** \| \| ,846 \| \| 1 \| \| ,846 \| \| \| ,200 \| ,655 \| \| \| Error \| \| 472,810 \| \| 112 \| \| 4,222 \| \| \|  \|  \| \| \| Total \| \| 1886,119 \| \| 116 \| \|  \| \| \|  \|  \| \| \| Corrected Total \| \| 510,661 \| \| 115 \| \|  \| \| \|  \|  \| \| \| a. R Squared = ,074 (Adjusted R Squared = ,049) \| \| \| \| \| \| \| \| \| \| \| \| \| **Parameter Estimates** \| \| \| \| \| \| \| \| \| \| \| \| \| \| Dependent Variable: serpinb1 \| \| \| \| \| \| \| \| \| \| \| \| \| \| Parameter \| B \| \| Std. Error \| \| t \| \| Sig. \| 95% Confidence Interval \| \| \| \| \| \| Lower Bound \| \| \| Upper Bound \| \| \| Intercept \| 2,368 \| \| 1,212 \| \| 1,953 \| \| ,053 \| -,035 \| \| \| 4,770 \| \| \| [status=Ctrl] \| 1,069 \| \| ,384 \| \| 2,784 \| \| **,006** \| ,308 \| \| \| 1,830 \| \| \| [status=SCA2] \| 0^a^ \| \| . \| \| . \| \| . \| . \| \| \| . \| \| \| Age \| ,017 \| \| ,016 \| \| 1,062 \| \| ,291 \| -,015 \| \| \| ,048 \| \| \| Sex \| -,186 \| \| ,415 \| \| -,448 \| \| ,655 \| -1,009 \| \| \| ,637 \| \| \| a. This parameter is set to zero because it is redundant. \| \| \| \| \| \| \| \| \| \| \| \| \|   This analysis confirmed that significant differences appeared for SERPINB1 plasma levels between patients and controls.  2-ANCOVA including an indicator variable (highSERPINB1) for control high (> 5.5 ng/mL) SERPINB1 plasma levels   \| **Tests of Between-Subjects Effects** \| \| \| \| \| \| \| \| \| \| \| \| --- \| --- \| --- \| --- \| --- \| --- \| --- \| --- \| --- \| --- \| --- \| \| Dependent Variable: serpinb1 \| \| \| \| \| \| \| \| \| \| \| \| Source \| Type III Sum of Squares \| \| df \| \| Mean Square \| \| \| F \| \| Sig. \| \| Corrected Model \| 348,795^a^ \| \| 4 \| \| 87,199 \| \| \| 59,797 \| \| ,000 \| \| Intercept \| 52,003 \| \| 1 \| \| 52,003 \| \| \| 35,661 \| \| ,000 \| \| **Age** \| 5,908 \| \| 1 \| \| 5,908 \| \| \| 4,051 \| \| ,094 \| \| **Sex** \| ,809 \| \| 1 \| \| ,809 \| \| \| ,555 \| \| ,458 \| \| **Group** \| 2,512 \| \| 1 \| \| 2,512 \| \| \| 1,723 \| \| ,192 \| \| **highSERPINB1** \| 310,944 \| \| 1 \| \| 310,944 \| \| \| 213,230 \| \| **,000** \| \| Error \| 161,866 \| \| 111 \| \| 1,458 \| \| \|  \| \|  \| \| Total \| 1886,119 \| \| 116 \| \|  \| \| \|  \| \|  \| \| Corrected Total \| 510,661 \| \| 115 \| \|  \| \| \|  \| \|  \| \| a. R Squared = ,683 (Adjusted R Squared = ,672) \| \| \| \| \| \| \| \| \| \| \| \| **Parameter Estimates** \| \| \| \| \| \| \| \| \| \| \| \| \| \| Dependent Variable: serpinb1 \| \| \| \| \| \| \| \| \| \| \| \| \| \| Parameter \| \| B \| \| Std. Error \| \| t \| Sig. \| \| 95% Confidence Interval \| \| \| \| \| Lower Bound \| \| \| Upper Bound \| \| Intercept \| \| 6,332 \| \| ,763 \| \| 8,303 \| ,000 \| \| 4,821 \| \| \| 7,843 \| \| age \| \| ,019 \| \| ,009 \| \| 2,013 \| ,094 \| \| ,000 \| \| \| ,037 \| \| sex \| \| -,182 \| \| ,244 \| \| -,745 \| ,458 \| \| -,666 \| \| \| ,302 \| \| [group=Ctrl] \| \| ,304 \| \| ,232 \| \| 1,313 \| ,192 \| \| -,155 \| \| \| ,763 \| \| [group=SCA2] \| \| 0^a^ \| \| . \| \| . \| . \| \| . \| \| \| . \| \| [highSERPINB1=No] \| \| -4,452 \| \| ,305 \| \| -14,602 \| **,000** \| \| -5,056 \| \| \| -3,848 \| \| [highSERPINB1=Yes] \| \| 0^a^ \| \| . \| \| . \| . \| \| . \| \| \| . \| \| a. This parameter is set to zero because it is redundant. \| \| \| \| \| \| \| \| \| \| \| \| \|   This analysis shows that the significant effect of the “group” on SERPINB1 plasma levels observed in the baseline ANCOVA, is lost after the inclusion of a variable (highSERPINB1) controlling for cases with > 5.5 ng/mL SERPINB1 plasma levels. | |
| --- | --- | --- | --- | --- | --- | --- | --- | --- | --- | --- | --- | --- | --- | --- | --- | --- | --- | --- | --- | --- | --- | --- | --- | --- | --- | --- | --- | --- | --- | --- | --- | --- | --- | --- | --- | --- | --- | --- | --- | --- | --- | --- | --- | --- | --- | --- | --- | --- | --- | --- | --- | --- | --- | --- | --- | --- | --- | --- | --- | --- | --- | --- | --- | --- | --- | --- | --- | --- | --- | --- | --- | --- | --- | --- | --- | --- | --- | --- | --- | --- | --- | --- | --- | --- | --- | --- | --- | --- | --- | --- | --- | --- | --- | --- | --- | --- | --- | --- | --- | --- | --- | --- | --- | --- | --- | --- | --- | --- | --- | --- | --- | --- | --- | --- | --- | --- | --- | --- | --- | --- | --- | --- | --- | --- | --- | --- | --- | --- | --- | --- | --- | --- | --- | --- | --- | --- | --- | --- | --- | --- | --- | --- | --- | --- | --- | --- | --- | --- | --- | --- | --- | --- | --- | --- | --- | --- | --- | --- | --- | --- | --- | --- | --- | --- | --- | --- | --- | --- | --- | --- | --- | --- | --- | --- | --- | --- | --- | --- | --- | --- | --- | --- | --- | --- | --- | --- | --- | --- | --- | --- | --- | --- | --- | --- | --- | --- | --- | --- | --- | --- | --- | --- | --- | --- | --- | --- | --- | --- | --- | --- | --- | --- | --- | --- | --- | --- | --- | --- | --- | --- | --- | --- | --- | --- | --- | --- | --- | --- | --- | --- | --- | --- | --- | --- | --- | --- | --- | --- | --- | --- | --- | --- | --- | --- | --- | --- | --- | --- | --- | --- | --- | --- | --- | --- | --- | --- | --- | --- | --- | --- | --- | --- | --- | --- | --- | --- | --- | --- | --- | --- | --- | --- | --- | --- | --- | --- | --- | --- | --- | --- | --- | --- | --- | --- | --- | --- | --- | --- | --- | --- | --- | --- | --- | --- | --- | --- | --- | --- | --- | --- | --- | --- | --- | --- | --- | --- | --- | --- | --- | --- | --- | --- | --- | --- | --- | --- | --- | --- | --- | --- | --- | --- | --- | --- | --- | --- | --- | --- | --- | --- | --- | --- | --- | --- | --- | --- | --- | --- | --- | --- | --- | --- | --- | --- | --- | --- | --- | --- | --- | --- | --- | --- | --- | --- | --- | --- | --- | --- | --- | --- | --- | --- | --- | --- | --- | --- | --- | --- | --- | --- | --- | --- | --- | --- | --- | --- | --- | --- | --- | --- | --- | --- | --- | --- | --- | --- | --- | --- | --- | --- | --- | --- | --- | --- | --- | --- | --- | --- | --- | --- | --- | --- | --- | --- | --- | --- | --- | --- | --- | --- | --- | --- | --- | --- | --- | --- | --- | --- | --- | --- | --- | --- | --- | --- | --- | --- | --- | --- | --- | --- | --- | --- | --- | --- | --- | --- | --- | --- | --- | --- | --- | --- | --- | --- | --- | --- | --- | --- | --- | --- | --- | --- | --- | --- | --- | --- | --- | --- | --- | --- | --- | --- | --- | --- | --- | --- | --- | --- | --- | --- | --- | --- | --- | --- | --- | --- | --- | --- | --- | --- | --- | --- | --- | --- | --- | --- | --- | --- | --- | --- | --- | --- | --- | --- | --- | --- | --- | --- | --- | --- | --- | --- | --- | --- | --- | --- | --- | --- | --- | --- | --- | --- | --- | --- | --- | --- | --- | --- | --- | --- | --- | --- | --- | --- | --- | --- | --- | --- | --- | --- | --- | --- | --- | --- | --- | --- | --- | --- | --- | --- | --- | --- | --- | --- | --- | --- | --- | --- | --- | --- | --- | --- | --- | --- | --- | --- | --- |
| 3- ANCOVA including the interaction of an indicator variable (highSERPINB1) for control high (> 5.5 ng/mL) SERPINB1 plasma levels with the group (patients vs controls)   \| **Tests of Between-Subjects Effects** \| \| \| \| \| \| \| --- \| --- \| --- \| --- \| --- \| --- \| \| Dependent Variable: SERPINB1 \| \| \| \| \| \| \| Source \| Type III Sum of Squares \| df \| Mean Square \| F \| Sig. \| \| Corrected Model \| 351,879^a^ \| 5 \| 70,376 \| 48,755 \| ,000 \| \| Intercept \| 45,323 \| 1 \| 45,323 \| 31,399 \| ,000 \| \| **age** \| 6,192 \| 1 \| 6,192 \| 4,290 \| ,101 \| \| **sex** \| ,451 \| 1 \| ,451 \| ,312 \| ,577 \| \| **group * highSERPINB1** \| 346,754 \| 3 \| 115,585 \| 80,074 \| **,000** \| \| Error \| 158,782 \| 110 \| 1,443 \|  \|  \| \| Total \| 1886,119 \| 116 \|  \|  \|  \| \| Corrected Total \| 510,661 \| 115 \|  \|  \|  \| \| a. R Squared = ,689 (Adjusted R Squared = ,675) \| \| \| \| \| \|  \| **Parameter Estimates** \| \| \| \| \| \| \| \| --- \| --- \| --- \| --- \| --- \| --- \| --- \| \| Dependent Variable: SERPINB1 \| \| \| \| \| \| \| \| Parameter \| B \| Std. Error \| t \| Sig. \| 95% Confidence Interval \| \| \| Lower Bound \| Upper Bound \| \| Intercept \| 5,601 \| ,909 \| 6,164 \| ,000 \| 3,800 \| 7,402 \| \| Age \| ,019 \| ,009 \| 2,071 \| ,101 \| ,001 \| ,037 \| \| Sex \| -,137 \| ,245 \| -,559 \| ,577 \| -,622 \| ,348 \| \| [status=Ctrl] * [highSERPINB1=No] \| -3,589 \| ,568 \| -6,314 \| **,000** \| -4,715 \| -2,463 \| \| [status=Ctrl] * [highSERPINB1=Yes] \| 1,150 \| ,623 \| 1,846 \| ,068 \| -,084 \| 2,384 \| \| [status=SCA2] * [highSERPINB1=No] \| -3,755 \| ,565 \| -6,643 \| **,000** \| -4,875 \| -2,635 \| \| [status=SCA2] * [highSERPINB1=Yes] \| 0^a^ \| . \| . \| . \| . \| . \| \| a. This parameter is set to zero because it is redundant. \| \| \| \| \| \| \| |  |
| This analysis shows that the interaction between the “group” and highSERPINB1 has a significant impact on SERPINB1 plasma levels. |  |

ANCOVA

| **Tests of Between-Subjects Effects** | | | | | |
| --- | --- | --- | --- | --- | --- |
| Dependent Variable: SERPINB1 | | | | | |
| Source | Type III Sum of Squares | df | Mean Square | F | Sig. |
| Corrected Model | 277,788^a^ | 6 | 46,298 | 23,881 | ,000 |
| Intercept | 16,576 | 1 | 16,576 | 8,550 | ,004 |
| **Group** | ,137 | 1 | ,137 | ,071 | ,791 |
| **Age** | 2,121 | 1 | 2,121 | 1,094 | ,299 |
| **Sex** | 3,436 | 1 | 3,436 | 1,772 | ,187 |
| **CAG repeat** | 4,425 | 1 | 4,425 | 2,282 | ,135 |
| **CtrlHighSERPINB1** | 245,079 | 1 | 245,079 | 126,413 | ,000 |
| **Group * CAG repeat** | ,916 | 1 | ,916 | ,472 | ,494 |
| Error | 157,036 | 81 | 1,939 |  |  |
| Total | 1622,826 | 88 |  |  |  |
| Corrected Total | 434,824 | 87 |  |  |  |
| a. R Squared = ,639 (Adjusted R Squared = ,612) | | | | | |

**Table S10**

Clinical and molecular characteristics of the extended cohort of patients with SCA2.

| Variables | SCA2 patients  (N=82) | |
| --- | --- | --- |
|  | Range | Mean (SD) |
| AO (yrs) | 15−62 | 39.36 (11.43) |
| DD (yrs) | 2−34 | 13.25 (6.46) |
| SARA score | 3.5−36 | 14.96 (7.43) |
| INAS count | 1−6 | 3.37 (1.45) |
| *ATXN2* expanded alleles (CAG repeat length) | 34−48 | 38.40 (2.83) |
| SERPINB1 (ng/ml) | 0.24−6.74 | 3.15 (1.64) |

AO− age at onset; DD− disease duration; SD− standard deviation
